# The gut microbe *Bacteroides fragilis* ameliorates renal fibrosis in mice

**DOI:** 10.1038/s41467-022-33824-6

**Published:** 2022-10-14

**Authors:** Wei Zhou, Wen-hui Wu, Zi-lin Si, Hui-ling Liu, Hanyu Wang, Hong Jiang, Ya-fang Liu, Raphael N. Alolga, Cheng Chen, Shi-jia Liu, Xue-yan Bian, Jin-jun Shan, Jing Li, Ning-hua Tan, Zhi-hao Zhang

**Affiliations:** 1 State Key Laboratory of Natural Medicines, Department of TCMs Pharmaceuticals, School of Traditional Chinese Pharmacy, China Pharmaceutical University, Nanjing, China; 2 Department of Nephrology, Renmin Hospital of Wuhan University, Wuhan, China; 3 Affiliated Hospital of Nanjing University of Chinese Medicine, Nanjing, China; 4 Ningbo Hospital of Zhejiang University, Ningbo, China; 5 Medical Metabolomics Center, Nanjing University of Chinese Medicine, Nanjing, China; 6 School of Life Science and Technology, China Pharmaceutical University, Nanjing, China

**Keywords:** Microbiome, Renal fibrosis

## Abstract

Renal fibrosis is an inevitable outcome of various manifestations of progressive chronic kidney diseases (CKD). The need for efficacious treatment regimen against renal fibrosis can therefore not be overemphasized. Here we show a novel protective role of *Bacteroides fragilis* (*B. fragilis*) in renal fibrosis in mice. We demonstrate decreased abundance of *B. fragilis* in the feces of CKD patients and unilateral ureteral obstruction (UUO) mice. Oral administration of live *B. fragilis* attenuates renal fibrosis in UUO and adenine mice models. Increased lipopolysaccharide (LPS) levels are decreased after *B. fragilis* administration. Results of metabolomics and proteomics studies show decreased level of 1,5-anhydroglucitol (1,5-AG), a substrate of SGLT2, which increases after *B. fragilis* administration via enhancement of renal SGLT2 expression. 1,5-AG is an agonist of TGR5 that attenuates renal fibrosis by inhibiting oxidative stress and inflammation. Madecassoside, a natural product found via in vitro screening promotes *B. fragilis* growth and remarkably ameliorates renal fibrosis. Our findings reveal the ameliorative role of B. *fragilis* in renal fibrosis via decreasing LPS and increasing 1,5-AG levels.

## Abstract (web summary)

Renal fibrosis is the main pathological feature of chronic kidney disease (CKD). Here, the authors show that live B. fragilis can attenuate renal fibrosis via the upregulation of SGLT2, which contributes to renal reabsorption of 1,5-AG, and that 1,5-AG improves renal fibrosis via inhibition of oxidative stress and inflammation.

## Introduction

It is estimated that approximately 10% of the general population suffers from chronic kidney disease (CKD)^1^. Renal fibrosis that inevitably results in CKD progression is characterized by the proliferation of fibroblasts and myofibroblasts. Myofibroblasts in turn are characterized by the production of alpha-smooth muscle actin (α-SMA) fibers, collagen and extracellular matrix (ECM) proteins. Incessant ECM production leads to decreased glomerular filtration rate and renal injury^2,3^. A total halt in disease progression or perhaps induction of renal fibrosis regression can alleviate CKD. Currently, the management of CKD involves the use of antihypertensive drugs, such as angiotensin-converting–enzyme inhibitors and angiotensin-receptor blockers, and the adoption of other blood pressure (BP)-controlling measures, including reductions in protein and salt intake. Keeping the BP and even blood glucose levels in check could avoid the incidence of acute kidney injury^4^. Aside from dialysis and surgery (i.e., kidney transplantation), there seems to be no available effective treatment for renal fibrosis and end-stage kidney disease^5^. This situation therefore calls for better and effective alternatives in the management and treatment of CKD.

The association of the gut microbiota with renal diseases has recently gained traction^6^. The gut microbiota composition comprises nearly 1 trillion microbes with diverse genetic make-up^7^. A healthy gut microbiota regulates physiological homeostasis;^8^ dysbiosis therefore could be an avenue for the promotion of chronic diseases such as kidney disease. There is a crosstalk between the kidneys and the gut in terms of their relationship with the gastrointestinal (GI) environment, gut epithelial barrier permeability and diseased state (such as CKD)^9^. There is a unique but reciprocal relationship between CKD and the gut microbiota. CKD incidence alters gut microbiota makeup and functions, eventually leading to dysbiosis^10^. On the other hand, the gut microbiota regulates various processes that result in CKD onset and progression. These include the accumulation of uremic toxins, such as indoxyl sulfate (IS), p-cresyl sulfate (p-CS) and trimethylamine n-oxidase (TMAO). The uremic toxins are produced by the increased abundance of *Enterobacteriaceae*, *Clostridiaceae*, *Pseudomonadaceae*, and *Bacteroidiaceae*, and the decreased levels of *Lactobacillaceae*, *Bifidobacteriaceae*, and *Prevotellaceae*^11,12^. Chronic inflammation and interruption of intestinal barrier function via increased ammonium production also enhanced the production of uremic toxins^13,14^. Besides, the reductions in CKD-protective metabolites derived from microbiota-like indolepropionic acid (IPA), short-chain fatty acids (SCFAs) produced by *Clostridium* and *Eubacterium* and the neurotransmitters (γ-aminobutyric acid and acetylcholine) produced by *Lactobacillaceae*, *Prevotellaceae* and *Bifidobacteriaceae* could accelerate the progression of CKD^12–15^. Thus, prevention of dysbiosis and restoration of homeostasis could be a potential strategy for the prevention and management of CKD.

In this study, we found that the abundance of *Bacteroides fragilis* (*B. fragilis*) in the fecal samples of CKD patients and UUO mice was significantly decreased. *B. fragilis* is an anaerobic obligate gram-negative commensal that resides in the lower gut of mammals and is known to influence their susceptibility to inflammatory diseases. *B. fragilis* strains have been shown to inhibit inflammation in different organs, including the peritoneum (such as intra-abdominal abscess)^16^, intestinal tract (intestinal bowel diseases)^17,18^, brain (autism spectrum disorder)^19^ and lungs (asthma)^20^, by producing polysaccharide A (PSA)^18^ and SCFA to stimulate interleukin-10-producing CD4^+^Foxp3^+^ T-regulatory cells^21^. However, there have been no reports on the role of *B. fragilis* in CKD. Therefore, both unilateral ureteral obstruction (UUO) and adenine mice models were first used to evaluate the effects of oral administration of *B. fragilis* on CKD. Then, untargeted and targeted metabolomics, together with label-free quantitative proteomics, were used to elucidate the mechanisms that underlie its renoprotective function. We have found that *B. fragilis* alleviates renal fibrosis by decreasing LPS levels. It also inhibits the Nrf2/Keap1 and TGF-β/Smad signaling pathways owing to increased levels of 1,5-anhydroglucitol (1,5-AG) in the blood. 1,5-AG was found to be a substrate of sodium-glucose cotransporter 2 (SGLT2). *B. fragilis* restored the reduced expression of SGLT2 in the kidneys of UUO and adenine models. We found 1,5-AG to be a TGR5 agonist, and knockdown of TGR5 abolished the anti-fibrotic effect of 1,5-AG and Nrf2/Keap1 activation in vitro. Finally, a natural product, madecassoside (Mad), was found to significantly promote *B. fragilis* growth and remarkably relieve renal fibrosis. Thus, regulating *B. fragilis* abundance in the gut might be a strategy to treat CKD.

## Results

### Gut microbial profiling in patients with CKD

**Fig. 1:**
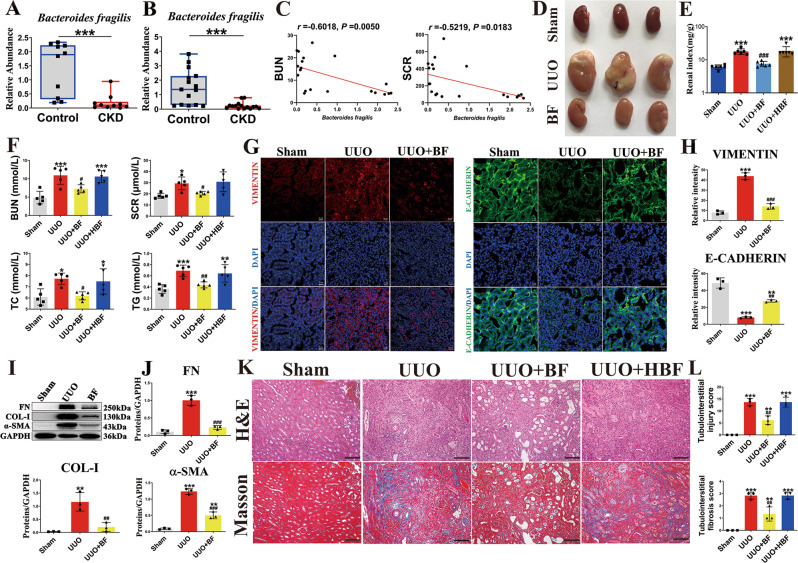
The anti-fibrotic effects of *B fragilis* in UUO model. **A** The relative abundance of *B. fragilis* in the CKD and control groups from Renmin Hospital of Wuhan University measured by qPCR (*n* = 10). ***p = 0.0005. **B** The relative abundance of *B. fragilis* in the CKD and control groups from the Putuo People’s Hospital measured by qPCR (*n* = 15). ***p < 0.0001. Box plots show center line as median, whiskers show maxima and minima, and box limits show upper and lower quartiles. **C** Pearson’s analysis of the correlations between *B. fragilis* level and the blood urea nitrogen (BUN) and serum creatinine (Scr). **D** Picture of left kidneys of mice with different treatments (*n* = 3). **E** The renal index (mg/g) was calculated by dividing the wet renal weight by the body weight (*n* = 6). ***p = 0.0001 for E: Sham vs. UUO, *p* = 0.9682 for E: Sham vs. UUO + BF, ***p  <  0.0001 for E: Sham vs. UUO + HBF, ^###^*p* = 0.0006 for E: UUO vs. UUO + BF, *p* = 0.9992 for E: UUO vs. UUO + HBF. **F** Biochemical parameters including blood urea nitrogen (BUN), serum creatinine (Scr), serum total cholesterol (TC), triglyceride (TG) in each of mice (*n* = 5). ***p = 0.0003 for BUN: Sham vs. UUO, *p* = 0.2261 for BUN: Sham vs. UUO + BF, ***p = 0.0006 for BUN: Sham vs. UUO + HBF, ^#^*p* = 0.0246 for BUN: UUO vs. UUO + BF, *p* = 0.9992 for BUN: UUO vs. UUO + HBF; *p = 0.0105 for SCR: Sham vs. UUO, ^#^*p* = 0.0436 for SCR: UUO vs. UUO + BF; *p = 0.0152 for TC: Sham vs. UUO, *p* = 0.9999 for TC: Sham vs. UUO + BF, *p = 0.0443 for TC: Sham vs. UUO + HBF, ^#^*p* = 0.0240 for TC: UUO vs. UUO + BF, *p* = 0.9915 for TC: UUO vs. UUO + HBF; ***p = 0.0007 for TG: Sham vs. UUO, *p* = 0.8042 for TG: Sham vs. UUO + BF, **p = 0.0031 for TG: Sham vs. UUO + HBF, ^##^*p* = 0.0070 for TG: UUO vs. UUO + BF, *p* = 0.9609 for TG: UUO vs. UUO + HBF. **G** Representative immunofluorescence staining of Vimentin and E-Cadherin in kidneys of mice as indicated (scale bar, 20 μm. magnification, ×200). **H** Quantitative analysis of Fig. 1G (*n* = 3). ***p  <  0.0001 for VIMENTIN: Sham vs. UUO, *p* = 0.0896 for VIMENTIN: Sham vs. UUO + BF, ^###^*p*  < 0.0001 for VIMENTIN: UUO vs. UUO + BF; ***p  <  0.0001 for E-CADHERIN: Sham vs. UUO, **p = 0.0011 for E-CADHERIN: Sham vs. UUO + BF, ^##^*p* = 0.0017 for E-CADHERIN: UUO vs. UUO + BF. **I** Kidney ex*p*ression of FN, Col I and α-SMA from Sham, UUO and *B. fragilis* -treated UUO mice, assayed by Western blot. **J** Quantitative analysis of Fig. 1I (*n* = 3). ***p  <  0.0001 for FN: Sham vs. UUO, *p* = 0.3994 for FN: Sham vs. UUO + BF, ^###^*p* = 0.0002 for FN: UUO vs. UUO + BF; **p = 0.0026 for COL-1: Sham vs. UUO, *p* = 0.7546 for COL-1: Sham vs. UUO + BF, ^##^*p* = 0.0062 for COL-1: UUO vs. UUO + BF; ***p  <  0.0001 for α-SMA: Sham vs. UUO, **p = 0.0024 for α-SMA: Sham vs. UUO + BF, ^###^*p*  < 0.0001 for α-SMA: UUO vs. UUO + BF. **K** Representative photomicrographs of the H&E staining and Masson’s trichrome staining from left kidneys of Sham, UUO, and *B. fragilis* -treated UUO mice (H&E and Masson’s staining; scale bar, 100 μm, magnification, ×200). **L** Bar graph depicts renal injury scores and renal interstitial fibrosis scores based on H&E staining or Masson’s trichrome staining (*n* = 3). ***p  < 0.0001 for injury scores: Sham vs. UUO, **p = 0.0064 for injury scores: Sham vs. UUO + BF, ***p  < 0.0001 for injury scores: Sham vs. UUO + HBF, ^##^*p* = 0.0018 for injury scores: UUO vs. UUO + BF, *p*  > 0.9999 for injury scores: UUO vs. UUO + HBF; ***p  < 0.0001 for fibrosis scores: Sham vs. UUO, **p = 0.0085 for fibrosis scores: Sham vs. UUO + BF, ***p  < 0.0001 for fibrosis scores: Sham vs. UUO + HBF, ^##^*p* = 0.0041 for fibrosis scores: UUO vs. UUO + BF, *p*  > 0.9999 for fibrosis scores: UUO vs. UUO + HBF. Data are presented as mean ± SD. Comparison in **A**, **B** were performed with a two-tailed Mann-Whitney U test. Comparisons in **E**, **F**, **H**, **J**, and **L** were compared using One-Way ANOVA followed by Sidak’s multiple comparisons test. ^*^P < 0.05, ^**^P < 0.01, ^***^P < 0.001 (compared with sham group), ^#^P < 0.05, ^##^P < 0.01, ^###^*P* < 0.001 (compared with UUO group). Individual data points are independent biological replicates unless otherwise stated.

We recruited 10 CKD patients and 10 age- and sex-matched healthy controls from Renmin Hospital of Wuhan University as microbiota discovery set. We performed a detailed and comparative gut microbial profiling using 16 S rDNA bacteria gene sequencing of their fecal samples. We found that the relative abundance of the phyla (*Bacteroidetes*, *Firmicutes* and *Bacteroidetes*/*Firmicutes* ratio) and genera (*Bacteroides*, *Streptococcus*, *Akkermansia*, *Bifidobacterium*, *Fecalibacterium* etc.) did not differ significantly (by false discovery rate adjustment) between the 2 groups (Supplementary Fig. 1A–1F). However, we found significantly decreased relative abundance of *B. fragilis* in patients with CKD by qPCR (Fig. 1A). We recruited another set of CKD patients (15 in total) and 15 age- and sex-matched healthy controls from the Putuo People’s Hospital as microbiota validation set. The abundance of *B. fragilis* in patients with CKD was also significantly decreased (Fig. 1B). Moreover, the abundance of *B. fragilis* had a significant negative correlation with BUN and Scr (Fig. 1C). Therefore, we focused on the *B. fragilis* to investigate the relationship between this specie and CKD and to clarify any underlying mechanisms.

### Oral administration of live *B. fragilis* attenuated renal fibrosis in UUO model

To determine the effect of *B. fragilis* on the progression of CKD, UUO mice were treated with live *B. fragilis* by daily oral gavage for 2 weeks. We examined the abundance of *B. fragilis* in the sham, UUO-, HF-, and BF-treated mice by qPCR (Supplementary Fig. 2). The results indicated that treatment with *B. fragilis* could restore the decreased *B. fragilis* level in the fecal sample from the UUO mice. Oral administration of live, but not heat-killed, *B. fragilis* significantly improved renal morphology and reduced renal index of the UUO mice (Fig. 1D, E). Treatment with B. *fragilis* significantly attenuated the increase in BUN, Scr, triglyceride and total cholesterol levels, suggesting an improved renal function in the UUO mice (Fig. 1F). Immunofluorescence showed a visibly decreased expression of vimentin protein and a visibly increased expression of E-Cadherin in the UUO mice treated with *B. fragilis* (Fig. 1G, H). The administration of *B. fragilis* greatly reduced the levels of several pro‑fibrotic markers, including pro-fibrotic proteins collagen I, fibronectin and alpha-smooth muscle actin (α-SMA) (Fig. 1I, J). Haematoxylin and eosin (H&E) and Masson staining showed that tubular dilatation, tubular atrophy, and widening of the interstitial space with severe inflammatory cell infiltration were attenuated by administration of *B. fragilis* (Fig. 1K, L). Used together, these findings indicate that the replenishment of live B. *fragilis* by oral gavage exerts strong anti-fibrotic effects in UUO mice.

### *B. fragilis* decreases LPS level and inhibits inflammation

The fecal and serum levels of LPS were assessed in UUO mice and CKD patients. The results indicated that fecal and serum LPS levels were higher in CKD patients and UUO-mice compared to the controls (Supplementary Fig. 3A–D). Moreover, we found that oral administration of live *B. fragilis* dramatically decreased the fecal and serum LPS concentrations in UUO mice (Supplementary Fig. 3C, D). *B. fragilis* treated mice had significantly lower fecal and serum levels of pro-inflammatory cytokines including interleukin (IL)−1β, IL-6 and tumor necrosis factor-α (TNF-α) (Supplementary Fig. 3E, F). The mRNA expressions of these pro-inflammatory cytokines were also decreased in mice treated with live *B. fragilis* (Supplementary Fig. 3G). These results showed that *B. fragilis* attenuated inflammation in UUO mice.

### *B. fragilis* attenuates renal fibrosis by inhibiting oxidative stress and the TGF-β/Smad signaling pathway in UUO model

**Fig. 2:**
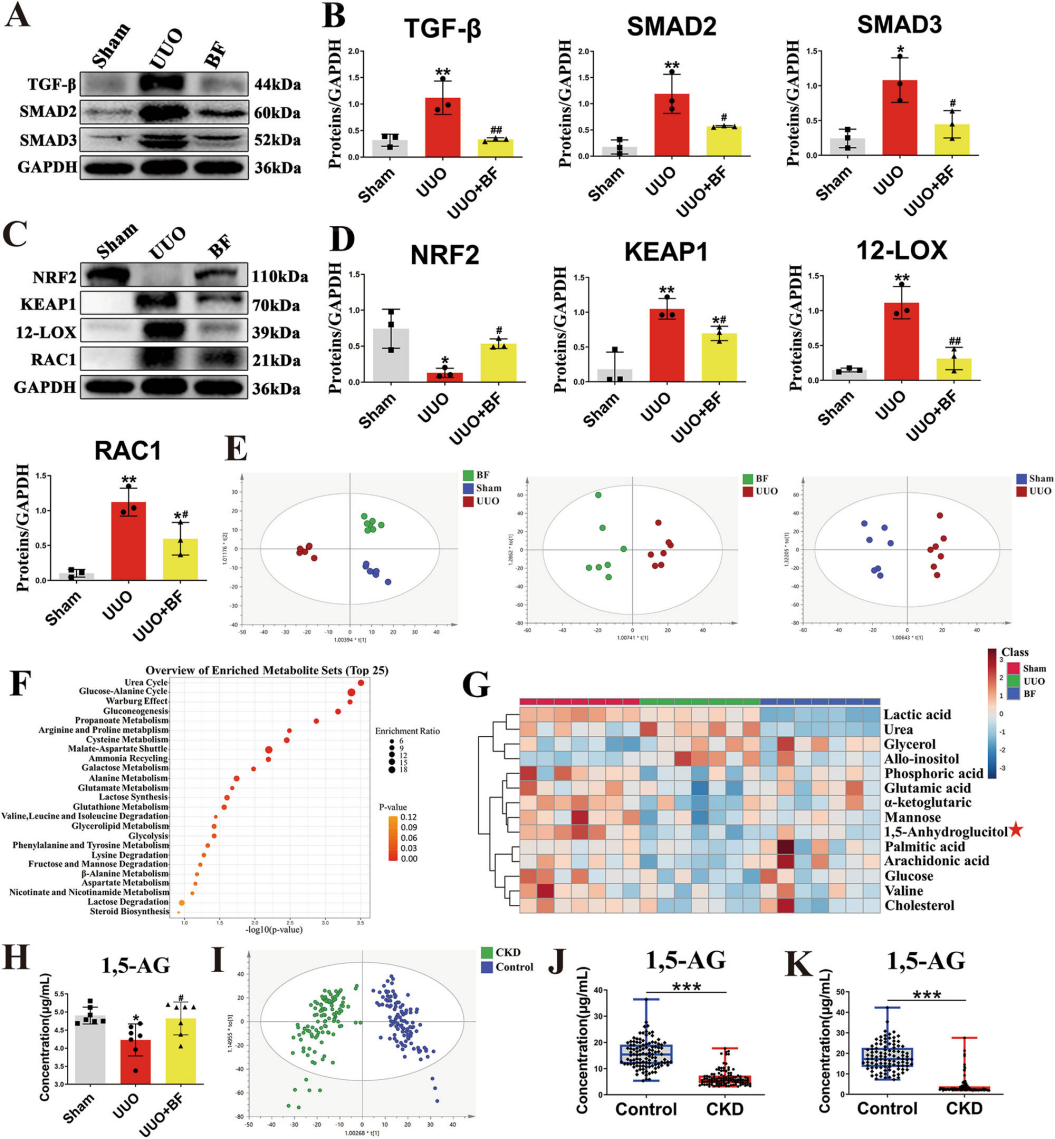
Inhibition of oxidative stress and the TGF-β/Smad signaling pathway by *B. fragilis* and the discovery of 1,5-AG by untargeted and targeted metabolomics. **A** Kidney expression of TGF-β/Smad signaling pathway from Sham, UUO and *B fragilis*-treated UUO mice, assayed by Western blot. **B** Quantitative analysis of panel **A** (*n* = 3). **p = 0.0070 for TGF-β: Sham vs. UUO, *p* = 0.9998 for TGF-β: Sham vs. UUO + BF, ^##^*p* = 0.0076 for TGF-β: UUO vs. UUO + BF; **p = 0.0049 for SMAD2: Sham vs. UUO, *p* = 0.2267 for SMAD2: Sham vs. UUO + BF, ^#^*p* = 0.0470 for SMAD2: UUO vs. UUO + BF; *p = 0.0128 for SMAD3: Sham vs. UUO, *p* = 0.6852 for SMAD3: Sham vs. UUO + BF, ^#^*p* = 0.0444 for SMAD3: UUO vs. UUO + BF. **C** Representative Western blot of Nrf2, Keap1, 12-LOX, Rac-1. **D** Quantitative analysis of panel **C** (*n* = 3). *p = 0.0118 for NRF2: Sham vs. UUO, *p* = 0.4377 for NRF2: Sham vs. UUO + BF, ^#^*p* = 0.0241 for NRF2: UUO vs. UUO + BF; **p = 0.0065 for KEAP1: Sham vs. UUO, *p = 0.0290 for KEAP1: Sham vs. UUO + BF, ^#^*p* = 0.0276 for KEAP1: UUO vs. UUO + BF, Comparison in KEAP1 were performed with a two-tailed Student’s t test; **p = 0.0010 for 12-LOX: Sham vs. UUO, *p* = 0.5931 for 12-LOX: Sham vs. UUO + BF, ^##^*p* = 0.0029 for 12-LOX: UUO vs. UUO + BF; **p = 0.0014 for RAC1: Sham vs. UUO, *p = 0.0455 for RAC1: Sham vs. UUO + BF, ^#^*p* = 0.0351 for RAC1: UUO vs. UUO + BF. **E** OPLS-DA in the indicated groups (*n* = 7). **F** Disturbed metabolic pathways for differential metabolites from sham vs UUO groups. **G** Heatmap of the differential metabolites. The asterisk indicates that the metabolite can be regulated by *B. fragilis*. **H** The concentration of 1,5-AG in serum from Sham, UUO and *B. fragilis*-treated UUO groups using GC-MS based targeted metabolomics (*n* = 7). *p = 0.0138 for H: Sham vs. UUO, *p* = 0.9746 for H: Sham vs. UUO + BF, ^#^*p* = 0.0317 for H: UUO vs. UUO + BF. **I** OPLS-DA plot of CKD patients (*n* = 115) and healthy controls (*n* = 113) of serum from the Affiliated Hospital of Nanjing University of Chinese Medicine through GC-MS based untargeted metabolomics. **J** GC-MS based targeted metabolomics of 1,5-AG in the sera of CKD and healthy controls (*n* = 110) from Ningbo Hospital of Zhejiang University. ***p  < 0.0001. **K** LC-MS based targeted metabolomics of 1,5-AG in the sera of CKD and controls (*n* = 100) from Putuo People’s Hospital. ***p  < 0.0001. Box plots show center line as median, whiskers show maxima and minima, and box limits show upper and lower quartiles. Data are presented as mean ± SD. Comparison in **J**, **K** were performed with a two-tailed Mann-Whitney U test. Comparisons in **B**, **D** and **H** were compared using One-Way ANOVA followed by Sidak’s multiple comparisons test. ^*^P < 0.05, ^**^P < 0.01, ^***^P < 0.001 (compared with sham or healthy control group), ^#^*P* < 0.05, ^##^*P* < 0.01, ^###^*P* < 0.001 (compared with UUO group)^.^ Individual data points are independent biological replicates unless otherwise stated.

The TGF-β/Smad pathway plays a key role in renal fibrosis^22^. The progression of CKD has been shown to result in inflammation and oxidative stress^23^. Thus, we evaluated the effect of *B. fragilis* on oxidative stress and TGF-β/Smad signaling pathway in UUO mice treated with *B. fragilis*. The expressions of TGF-β, Smad2, Smad3 in the UUO mice were all significantly increased compared to controls. Treatment with *B. fragilis* mediated significant attenuation of UUO-induced increase in TGF-β, Smad2 and Smad3 expressions (Fig. 2A, B). The mRNA levels of TGF-β, Smad2 and Smad3 exhibited similar patterns (Supplementary Fig. 4). Furthermore, we found that UUO caused significant increases in Keap1 and ROS-generating molecules including 12-Lox and Rac1. However, the expression of antioxidant protein (Nrf2) was significantly downregulated in the UUO mice (Fig. 2C, D). After treatment with *B. fragilis*, the upregulation of Keap1, 12-Lox and Rac1 was significantly reduced, while the downregulation of Nrf2 was significantly enhanced (Fig. 2C, D). In addition, the mRNA levels of Keap1, Rac1, p67 (Ncf2) and p47 (Nsfl1c) exhibited similar patterns (Supplementary Fig. 5A). These results indicate that *B. fragilis* can inhibit oxidative stress and the TGF-β/Smad signaling pathway in the UUO mice.

### *B. fragilis* alters CKD-associated 1,5-AG levels in UUO model

The profound influence of gut microbiota on the host is strongly associated with complex interactions comprising a series of host-microbe metabolic axes^24^. To assess metabolic alterations in response to the treatment with *B. fragilis*, metabolome profiles were generated from serum samples by untargeted metabolomics using gas chromatography–mass spectrometry (GC-MS). A supervised OPLS-DA was performed using data from sham, UUO and UUO + BF group. OPLS-DA score plots could readily be divided into several clusters (Fig. 2E), indicating that the metabolic states of all UUO mice were significantly changed in relation to the sham mice. Additionally, a clear distinction in the metabolomes between UUO group and UUO + BF group was observed (Fig. 2E), suggesting the metabolic states of UUO + BF group were significantly different from the UUO group. An unsupervised PCA was also performed using the aforementioned data and the PCA plots exhibited patterns similar to the OPLS-DA plots (Supplementary Fig. 6A). Fourteen differentially expressed metabolites between the sham and UUO groups were identified by variable importance in the projection (VIP) ≥ 1.0 and adjusted *p* values ≤ 0.05 (Supplementary Table 1). A systematic pathway analysis based on these 14 differential metabolites is shown in Fig. 2F. Then, a heatmap was created to visualize the relative levels of the 14 differential metabolites in each group (Fig. 2G). Notably, of these 14 identified metabolites, 1,5-AG has been reported as a reliable glycaemic marker in type 2 diabetics with CKD^25^. Moreover, low 1,5-AG levels are associated with higher risk of incident ESRD independent of baseline kidney function^26^. In this study, the serum levels of 1,5-AG were significantly decreased in the UUO mice, while treatment with *B. fragilis* significantly upregulated the decreased level of 1,5-AG using GC-MS based targeted metabolomics (Fig. 2H). The results indicate that treatment with *B. fragilis* can significantly upregulate the serum level of 1,5-AG in UUO mice.

### 1,5-AG levels in healthy controls and CKD patients

We conducted GC-MS based untargeted metabolomics to evaluate the inherent differential serum metabolic profiles between healthy controls and CKD patients (115 patients and 113 healthy subjects from the Affiliated Hospital of Nanjing University of Chinese Medicine). The OPLS-DA results indicated significant alteration in metabolic profiles between the healthy controls and CKD patients (Fig. 2I). An unsupervised PCA was also performed on the same data and the PCA plots exhibited patterns similar to the OPLS-DA plots (Supplementary Fig. 6B). Differential metabolites were identified and summarized in Supplementary Table 2. A systematic pathway analysis based on 23 differential metabolites is shown in Supplementary Fig. 7A. Then, a heatmap was created to visualize the relative levels of the 23 differential metabolites in each group, and the level of 1,5-AG was significantly reduced in the CKD patients relative to the healthy controls (Supplementary Fig. 7B). We also confirmed the significantly decreased serum level of 1,5-AG in CKD patients by external validation 1 using GC-MS based targeted metabolomics (110 patients and 110 healthy subjects from Ningbo Hospital of Zhejiang University) (Fig. 2J). The accurate quantification of 1,5-AG showed that the concentration of 1,5-AG in serum was 15.15 ± 5.21 μg/mL in the healthy control group, and 5.56 ± 3.11 μg/mL in the CKD group. In addition, we further confirmed the significantly decreased serum level of 1,5-AG in CKD patients by external validation 2 using LC-MS based targeted metabolomics (100 patients and 100 healthy subjects from the Putuo People’s Hospital) (Fig. 2K). The concentration of 1,5-AG in serum was 18.24 ± 6.64 μg/mL in the control group, and 4.24 ± 4.43 μg/mL in the CKD group.

### 1,5-AG attenuates renal fibrosis in UUO model

**Fig. 3:**
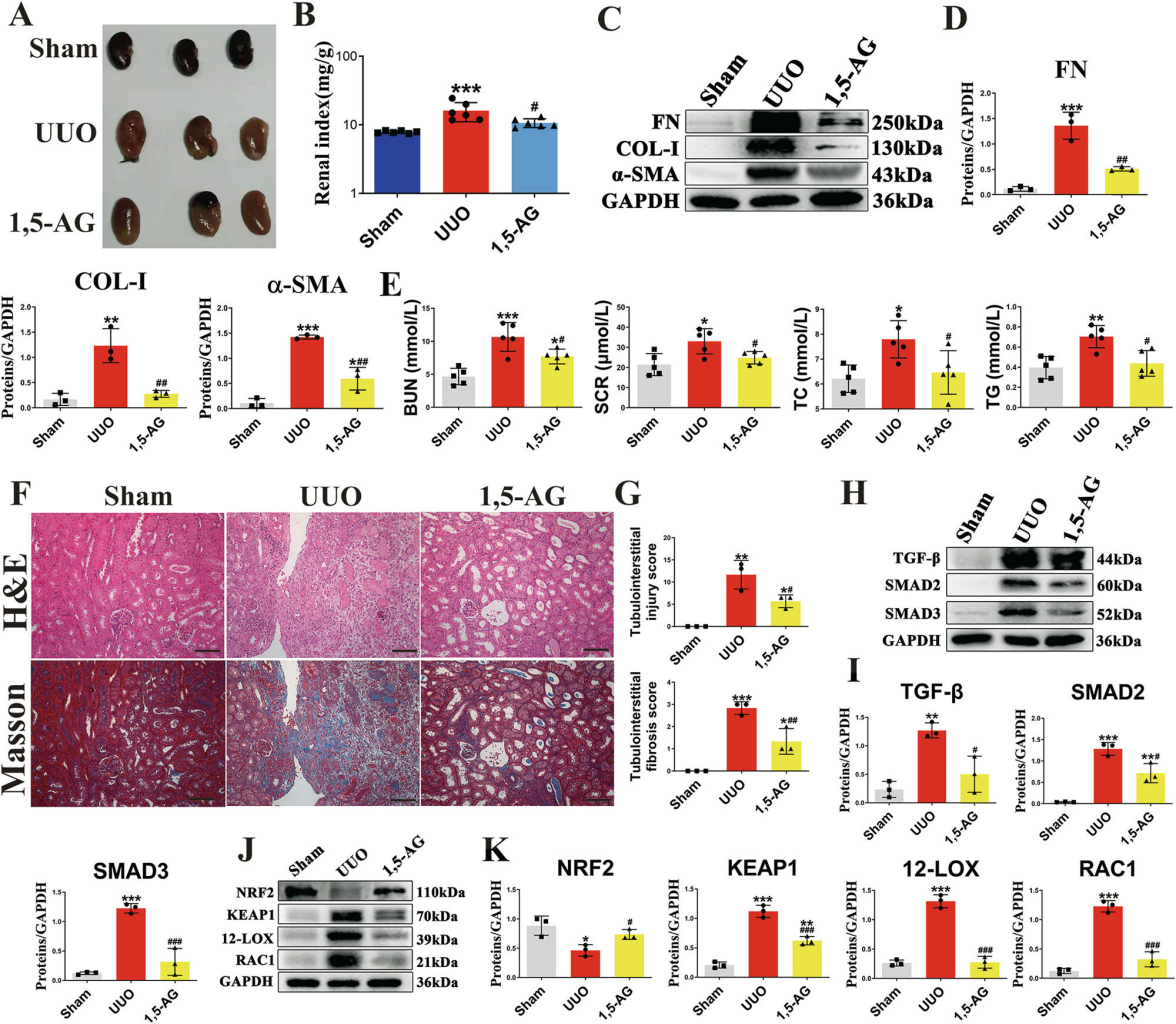
The anti-fibrotic effects of 1, 5-AG in UUO model. **A** Picture of left kidneys of mice with different treatments (*n* = 3). **B** The renal index (mg/g) (*n* = 6). ***p = 0.0008 for B: Sham vs. UUO, *p* = 0.3372 for B: Sham vs. 1, 5-AG, ^#^*p* = 0.0221 for B: UUO vs. 1, 5-AG. **C** Western blots show fibrotic protein expression in kidneys from all groups. **D** Quantitative analysis of fibrotic protein (n=3). ***p = 0.0002 for FN: Sham vs. UUO, *p* = 0.0637 for FN: Sham vs. 1, 5-AG, ^##^*p* = 0.0017 for FN: UUO vs. 1, 5-AG; **p = 0.0024 for COL-1: Sham vs. UUO, *p* = 0.9068 for COL-1: Sham vs. 1, 5-AG, ^##^*p* = 0.0043 for COL-1: UUO vs. 1, 5-AG; ***p < 0.0001 for α-SMA: Sham vs. UUO, *p = 0.0170 for α-SMA: Sham vs. 1, 5-AG, ^##^*p* = 0.0011 for α-SMA: UUO vs. 1, 5-AG. **E** Biochemical parameters including BUN, Scr, TC, TG in each of mice (*n* = 5). ***p = 0.0002 for BUN: Sham vs. UUO, *p = 0.0319 for BUN: Sham vs. 1, 5-AG, ^#^*p* = 0.0361 for BUN: UUO vs. 1, 5-AG; *p = 0.0146 for SCR: Sham vs. UUO, *p* = 0.2637 for SCR: Sham vs. 1, 5-AG, ^#^*p* = 0.0309 for SCR: UUO vs. 1, 5-AG, Comparison in SCR were performed with a two-tailed Student’s t test; *p = 0.0159 for TC: Sham vs. UUO, *p* = 0.9351 for TC: Sham vs. 1, 5-AG, ^#^*p* = 0.0429 for TC: UUO vs. 1, 5-AG; **p = 0.0038 for TG: Sham vs. UUO, *p* = 0.9255 for TG: Sham vs. 1, 5-AG, ^#^*p* = 0.0107 for TG: UUO vs. 1, 5-AG. **F** Representative photomicrographs of the H&E staining and Masson’s trichrome staining from left kidneys of Sham, UUO, and 1, 5-AG-treated UUO mice (H&E and Masson’s staining; scale bar, 100 μm; magnification, ×200). **G** Bar graphs depict renal injury scores and renal interstitial fibrosis scores based on H&E staining or Masson’s trichrome staining (*n* = 3). **p = 0.0012 for injury scores: Sham vs. UUO, *p = 0.0423 for injury scores: Sham vs. 1, 5-AG, ^#^*p* = 0.0332 for injury scores: UUO vs. 1, 5-AG; ***p = 0.0003 for fibrosis scores: Sham vs. UUO, *p = 0.0139 for fibrosis scores: Sham vs. 1, 5-AG, ^##^*p* = 0.0079 for fibrosis scores: UUO vs. 1, 5-AG. **H** Kidney expression of TGF-β/Smad signaling pathway from Sham, UUO and 1, 5-AG-treated UUO mice, assayed by Western blot. (I) Quantitative analysis of Fig. 3H (*n* = 3). **p = 0.0031 for TGF-β: Sham vs. UUO, *p* = 0.4396 for TGF-β: Sham vs. 1, 5-AG, ^#^*p* = 0.0138 for TGF-β: UUO vs. 1, 5-AG; ***p = 0.0002 for SMAD2: Sham vs. UUO, **p = 0.0053 for SMAD2: Sham vs. 1, 5-AG, ^#^*p* = 0.0126 for SMAD2: UUO vs. 1, 5-AG; ***p = 0.0002 for SMAD3: Sham vs. UUO, *p* = 0.3792 for SMAD3: Sham vs. 1, 5-AG, ^###^*p* = 0.0006 for SMAD3: UUO vs. 1, 5-AG. **J** Representative Western blot of Nrf2, Keap1, 12-LOX, Rac-1. (K) Quantitative analysis of Fig. 3J (*n* = 3). *p = 0.0156 for NRF2: Sham vs. UUO, *p* = 0.4461 for NRF2: Sham vs. 1, 5-AG, ^#^*p* = 0.0331 for NRF2: UUO vs. 1, 5-AG; ***p  < 0.0001 for KEAP1: Sham vs. UUO, **p = 0.0019 for KEAP1: Sham vs. 1, 5-AG, ^###^*p* = 0.0007 for KEAP1: UUO vs. 1, 5-AG; ***p  < 0.0001 for 12-LOX: Sham vs. UUO, *p* = 0.9980 for 12-LOX: Sham vs. 1, 5-AG, ^###^*p*  < 0.0001 for 12-LOX: UUO vs. 1, 5-AG; ***p  < 0.0001 for RAC1: Sham vs. UUO, *p* = 0.1273 for RAC1: Sham vs. 1, 5-AG, ^###^*p*  < 0.0001 for RAC1: UUO vs. 1, 5-AG. Data are presented as mean ± SD. Comparisons in **B**, **D**, **E**, **G**, **I** and **K** were compared using One-Way ANOVA followed by Sidak’s multiple comparisons test. ^*^P < 0.05, ^**^P < 0.01, ^***^P < 0.001 (compared with sham group), ^#^*P* < 0.05, ^##^*P* < 0.01, ^###^*P* < 0.001 ^(^compared with UUO group). Individual data points are independent biological replicates unless otherwise stated.

To assess whether *B. fragilis* attenuates renal fibrosis by upregulation of the level of 1,5-AG, we examined the renoprotective effect of 1,5-AG in UUO-mice. Administration of 1,5-AG significantly improved renal morphology of the UUO-mice (Fig. 3A), reduced renal index of the UUO-mice (Fig. 3B) and reduced the levels of several pro‑fibrotic markers, including pro-fibrotic proteins collagen I, fibronectin and alpha-smooth muscle actin (α-SMA) (Fig. 3C, D). Treatment with 1,5-AG significantly attenuated the increase in BUN, Scr, triglyceride and total cholesterol levels, suggesting that 1,5-AG improved renal function (Fig. 3E). H&E and Masson staining showed that tubular dilatation, tubular atrophy, and widening of the interstitial space with severe inflammatory cell infiltration were attenuated by 1,5-AG (Fig. 3F, G). In addition, 1,5-AG decreased LPS level and inhibited inflammation in UUO mice (Supplementary Fig. 8). Moreover, 1,5-AG activated the Nrf2/Keap1 and inhibited TGF-β/Smad signaling pathways in UUO mice (Fig. 3H–K, Supplementary Fig. 5B). Taken together these results indicate that 1,5-AG significantly attenuated renal fibrosis in vivo.

### 1,5-AG upregulates renal TGR5 expression in vitro and in vivo

**Fig. 4:**
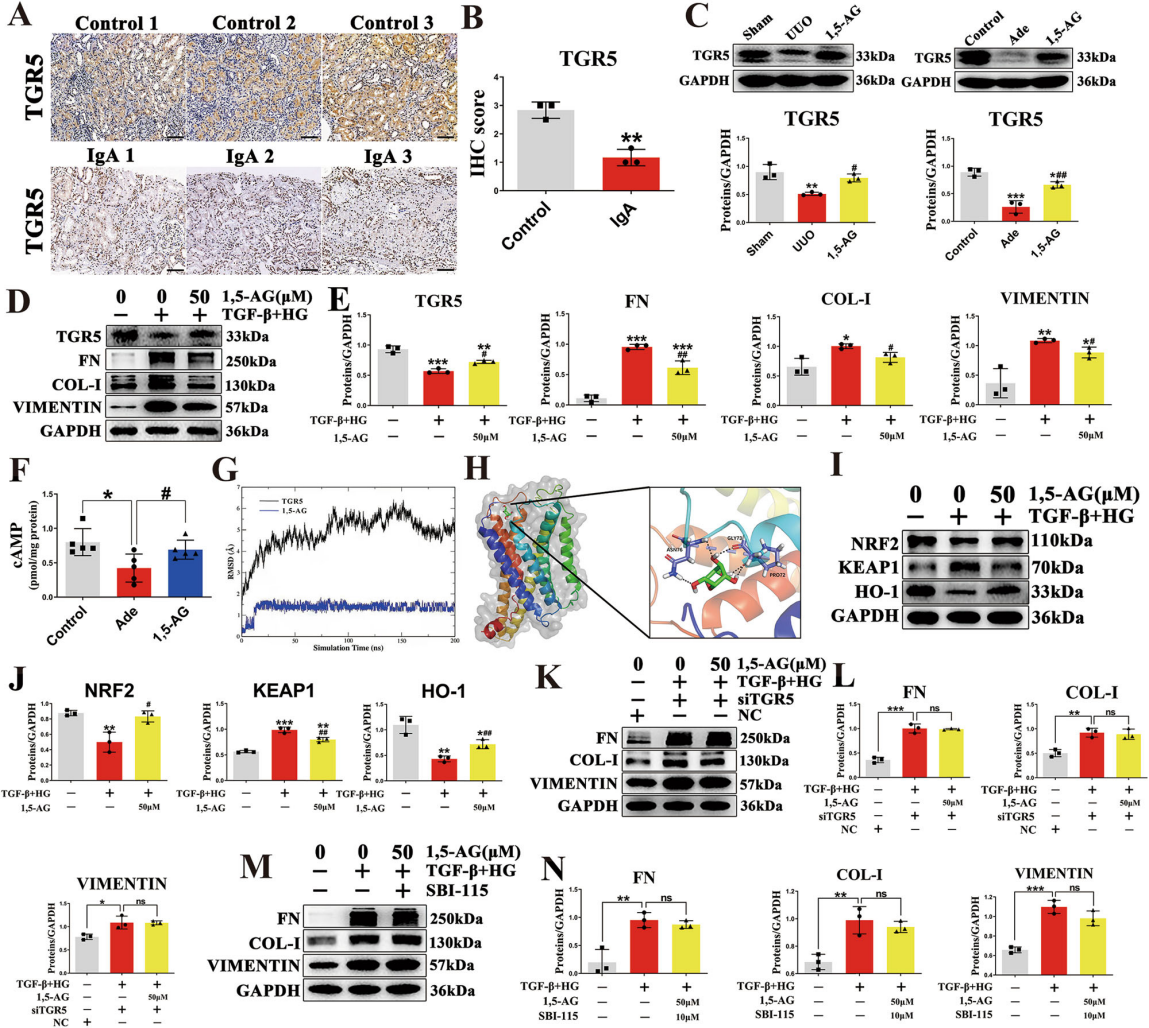
1,5-AG as an agonist of TGR5 and knockdown of TGR5 abolishing the anti-fibrotic effect of 1,5-AG. **A** Immunohistochemical micrographs of TGR5 in the kidney tissues of IgA patients (scale bar, 100 μm). **B** IHC scores of Fig. 4A (*n* = 3) **p =0.0021. **C** TGR5 protein expressions of kidney in UUO and adenine models. **p = 0.0054 for UUO model: Sham vs. UUO, *p* = 0.5034 for UUO model: Sham vs. 1, 5-AG, ^#^*p* = 0.0237 for UUO model: UUO vs. 1, 5-AG; ***p = 0.0003 for adenine model: Control vs. Ade, *p = 0.0446 for adenine model: Control vs. 1, 5-AG, ^##^*p* = 0.0034 for adenine model: Ade vs. 1, 5-AG. **D** TGR5, FN, Col-I and Vimentin expressions in primary mouse renal tubular cells (PRTC) after treatment with TGF-β (10 ng/mL) + high glucose (30 mM) and 1,5-AG (50 μM). **E** Quantitative analysis of Fig. 4D (*n* = 3). ***p = 0.0001 for TGR5: Control vs. Model, **p = 0.0028 for TGR5: Control vs. 1, 5-AG, ^#^*p* = 0.0137 for TGR5: Model vs. 1, 5-AG; ***p  < 0.0001 for FN: Control vs. Model, ***p = 0.0006 for FN: Control vs. 1, 5-AG, ^##^*p* = 0.0043 for FN: Model vs. 1, 5-AG; *p = 0.0149 for COL-1: Control vs. Model, *p* = 0.1713 for COL-1: Control vs. 1, 5-AG, ^#^*p* = 0.0247 for COL-1: Model vs. 1, 5-AG, Comparison in COL-1 were performed with a two-tailed Student’s t test; **p = 0.0075 for VIMENTIN: Control vs. Model, *p = 0.0270 for VIMENTIN: Control vs. 1, 5-AG, ^#^*p* = 0.0231 for VIMENTIN: Model vs. 1, 5-AG, Comparison in VIMENTIN were performed with a two-tailed Student’s t test. **F** The cAMP levels in adenine model. *p = 0.0130 for cAMP: Control vs. Ade, ^#^*p* = 0.0376 for cAMP: Ade vs. 1, 5-AG. **G** The root mean square deviation (RMSD) of protein backbone atoms and 1,5-AG during the molecular dynamics (MD) simulation. **H** Molecular docking and the binding mode of 1,5-AG to TGR5 through the MD simulation. **I** Representative Western blot of Nrf2, Keap1, and HO-1. **J** Quantitative analysis of Fig. 4I (*n* = 3). **p = 0.0058 for NRF2: Control vs. Model, *p* = 0.9139 for NRF2: Control vs. 1, 5-AG, ^#^*p* = 0.0107 for NRF2: Model vs. 1, 5-AG; ***p  < 0.0001 for KEAP1: Control vs. Model, **p = 0.0015 for KEAP1: Control vs. 1, 5-AG, ^##^*p* = 0.0048 for KEAP1: Model vs. 1, 5-AG; **p = 0.0029 for HO-1: Control vs. Model, *p = 0.0261 for HO-1: Control vs. 1, 5-AG, ^##^*p* = 0.0097 for HO-1: Model vs. 1, 5-AG, Comparison in HO-1 were performed with a two-tailed Student’s t test. **K** FN, Col-I and Vimentin expressions in PRTC after treatment with siRNA against TGR5 or negative control. **L** Quantitative analysis of panel **K** (*n* = 3). ***p < 0.0001 for FN; **p = 0.0027 for COL-1; *p = 0.0117 for VIMENTIN. **M** The effect of SBI-115 on anti-fibrotic effect of 1,5-AG in PRTC. **N** Quantitative analysis of panel **M** (*n* = 3). **p = 0.0023 for FN; **p = 0.0036 for COL-1; ***p = 0.0002 for VIMENTIN. Data are presented as mean ± SD. Comparison in **B** were performed with a two-tailed Student’s t test. Comparisons in **C**, **E**, **F**, **J**, **L** and **N** were compared using One-Way ANOVA followed by Sidak’s multiple comparisons test. ^*^P < 0.05, ^**^P < 0.01, ^***^P < 0.001 (compared with sham or control group), ^#^*P* < 0.05, ^##^*P* < 0.01, ^###^*P* < 0.001 (compared with model group). Individual data points are independent biological replicates unless otherwise stated.

The transmembrane G protein-coupled bile acid receptor (TGR5) is a cell membrane bile acid receptor, which is closely associated with fibrosis and inflammation^27,28^. In this study, immunohistochemical staining showed that the TGR5 levels of the IgA nephropathic patients were markedly decreased (Fig. 4A, B). We then examined the expression of TGR5 in UUO and adenine models. Consistent with the results in human subjects, the results indicated that the levels of TGR5 were significantly decreased in the UUO and adenine mice (Fig. 4C). Treatment with 1,5-AG significantly upregulated the levels of TGR5 (Fig. 4C).

We further examined the protective effects of 1,5-AG against fibrosis and inflammation in vitro. Treatment with 1,5-AG significantly inhibited the expression of the pro-fibrotic mediators (collagen I, vimentin, fibronectin) in HK-2 cells and inflammatory markers (TNF-α, IL-1β, IL-6) in HMC cells (Supplementary Fig. 9A–D). The mRNA expressions of these pro-inflammatory cytokines (IL-1β, IL-6, P67) were also decreased in HMC cells treated with 1,5-AG (Supplementary Fig. 9E). In TGF-β1 + high glucose-induced primary mouse renal tubular cells (PRTC), 1,5-AG significantly inhibited the expression of the pro-fibrotic mediators (collagen I, vimentin, fibronectin) (Fig. 4D, E). In addition, the levels of TGR5 were significantly restored after treatment with 1,5-AG in PRTC (Fig. 4D, E). TGR5 activation increases cAMP production. Therefore, we examined the effects of 1,5-AG on cAMP levels. The results indicated that 1,5-AG increased cAMP levels in adenine mice (Fig. 4F). To explore the binding of 1,5-AG to TGR5, in silico molecular docking and then molecular dynamics (MD) simulation were performed. The root mean square deviation (RMSD) of protein backbone atoms and 1,5-AG during the MD simulations is shown in Fig. 4G. The RMSD of TGR5 increased to 6 Å in 100 ns and then equilibrated at approximately 5.5 Å. 1,5-AG was stably bound at the pocket during all of the MD simulation. The binding mode of 1,5-AG to TGR5 was determined through the MD simulation. As shown in Fig. 4H, 1,5-AG formed five hydrogen bonds with TGR5 by interacting with ASN76, GLY73 and PRO72. The binding energy calculated through the Molecular Mechanics-Poisson Bolzmann Surface Area (MM-PBSA) method was −4.48 kcal mol^−1^. These results indicated that 1,5-AG might bind to TGR5. Taken together, these results suggested that 1,5-AG is an agonist for the TGR5 receptor. It has been reported that TGR5 attenuated liver ischemia–reperfusion injury by activating the Nrf2/Keap1 signaling pathway in mice^29^. In this study, we have demonstrated that 1,5-AG inhibited oxidative stress by activating the Nrf2/Keap1 signaling pathway in PRTC (Fig. 4I, J).

### Knockdown of TGR5 abolishes the anti-fibrotic effect of 1,5-AG in vitro

To investigate whether the anti-fibrotic effect of 1,5-AG relies on the TGR5 receptor, we knocked down TGR5 expression in PRTC. TGR5 expression in the knockdown group was visibly reduced compared to the control group (Supplementary Fig. 10A, B). Treatment with 1,5-AG could significantly decrease the pro-fibrotic proteins including collagen I, vimentin, and fibronectin in PRTC (Fig. 4D, E). However, the knockdown of TGR5 abolished the protective effect of 1,5-AG in PRTC (Fig. 4K, L). Likewise, the attenuation of the pro-fibrotic proteins observed after 1,5-AG treatment was reversed by the administration of SBI-115, a potent selective antagonist of TGR5 (Fig. 4M, N).

### *B. fragilis* upregulates renal SGLT2 expression in UUO model

**Fig. 5:**
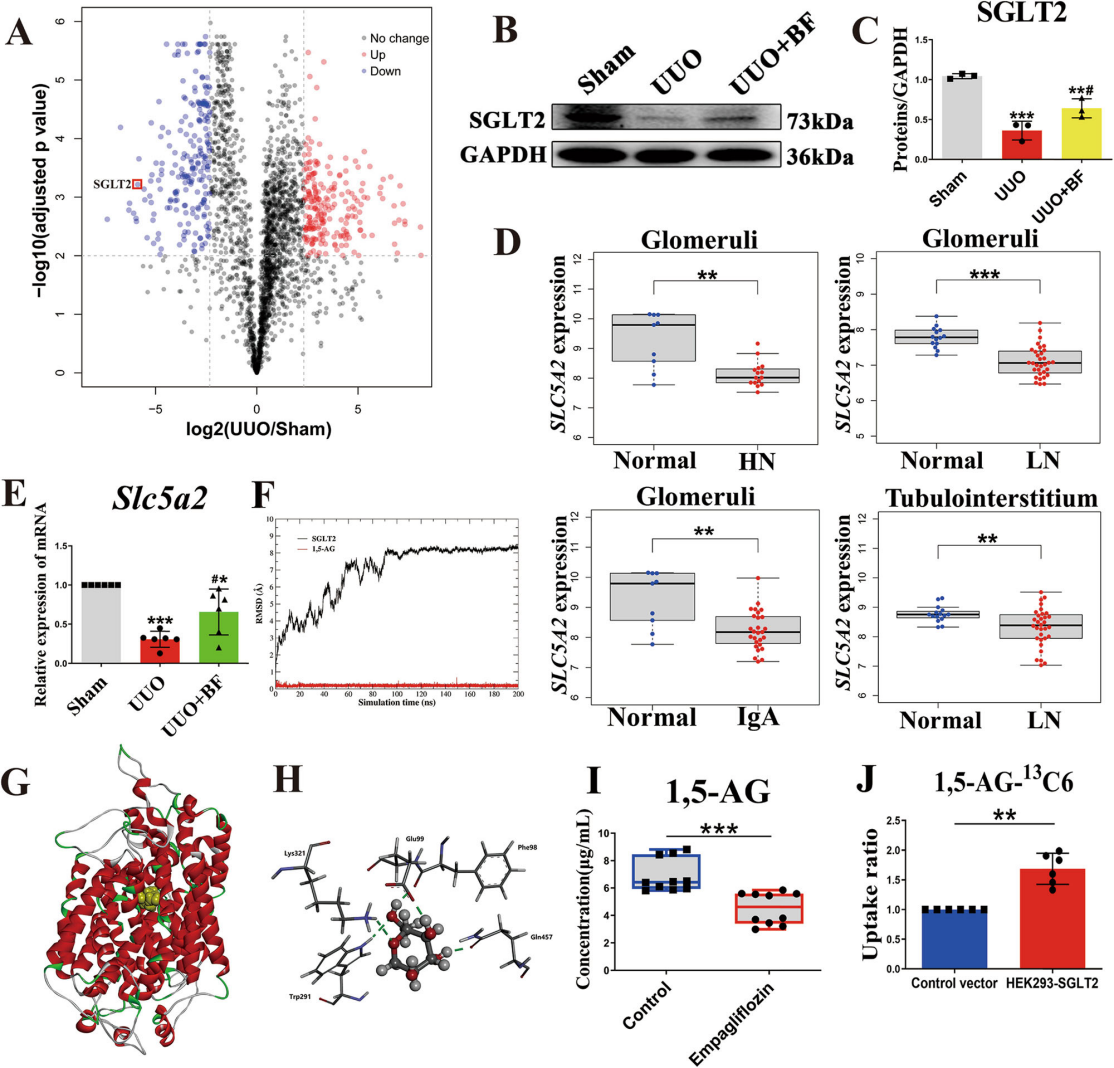
SGLT2 as a 1,5-AG transporter in the kidney. **A** Differentially expressed proteins of kidney tissues in UUO *vs* Sham are highlighted with volcano plot. The blue dots indicate the downregulated proteins, and the red dots indicate the upregulated proteins. **B** Western blot shows SGLT2 protein expression in kidneys from sham, UUO and UUO + BF (*B. fragilis*). **C** Quantitative analysis of panel **B** (*n* = 3). ***p = 0.0004 for SGLT2: Sham vs. UUO, **p = 0.0072 for SGLT2: Sham vs. UUO + BF, ^#^*p* = 0.0394 for SGLT2: UUO vs. UUO + BF. **D** mRNA level of Slc5a2 was investigated in the GEO database using 153 subjects. Log-transformed SLC5A2 mRNA level was compared not only between glomeruli from healthy people (Normal) (*n* = 14) and lupus nephritic patients (LN) (*n* = 32), but also between tubulointerstitium from healthy people (*n* = 15) and lupus nephritic patients (*n* = 32) using Mann-Whitney U test; Log-transformed SLC5A2 mRNA level was compared not only between glomeruli from healthy people (*n* = 9) and hypertensive nephropathic patients (HN) (*n* = 9), but also between glomeruli from healthy people (*n* = 15) and IgA nephropathic patients (*n* = 27) using Mann-Whitney U test. **p = 0.0083 for Glomeruli HN; ***p < 0.0001 for Glomeruli LN; **p = 0.0096 for Glomeruli IgA; **p = 0.0091 for Tubulointerstitium LN. **E** mRNA level of SLC5A2 in the indicated groups (*n* = 6). ***p  < 0.0001 for Slc5a2: Sham vs. UUO, *p = 0.0140 for SGLT2: Sham vs. UUO + BF, ^#^*p* = 0.0124 for SGLT^2^: UUO vs. UUO + BF. **F** The root mean square deviation (RMSD) of protein backbone atoms and 1,5-AG during the molecular dynamics (MD) simulation. **G**, **H** Molecular docking and the binding mode of 1,5-AG to SGLT2 through the MD simulation. **I** Empagliflozin significantly decreased the serum concentration of 1,5-AG in control mice (*n* = 10). ***p = 0.0003. Box plots show center line as median, whiskers show maxima and minima, and box limits show upper and lower quartiles. **J** Cellular uptake experiments of 1,5-AG-^13^C_6_ were performed in stably SGLT2 transfected HEK293 *vs* wide-type (WT) cells (*n* = 6). ***p = 0.0022. Data are *p*resented as mean ± SD. Comparison in D was performed with a two-tailed Mann-Whitney U test. Comparison in **I** and **J** were performed with a two-tailed Student’s t test. Comparisons in **C**, **E** were compared using One-Way ANOVA followed by Sidak’s multiple comparisons test. ^*^P < 0.05, ^**^P < 0.01, ^***^P < 0.001 (compared with sham group or normal subjects), ^#^*P* < 0.05, ^##^*P* < 0.01, ^###^*P* < 0.001 (compared with UUO group)^.^ Individual data points are independent biological replicates unless otherwise stated.

The label-free quantitative proteomics was performed to profile the proteome differences between the sham and the UUO mice. Compared to the sham mice, we found 216 upregulated proteins and 215 downregulated proteins in the UUO mice based on the criterion of fold change ≥ 5, and adjusted-p value ≤ 0.01 (Supplementary data file, Supplementary Fig. 11B). Among them, SGLT2 expression was significantly downregulated in the UUO mice (Fig. 5A). GO and KEGG pathway analyses are presented in Supplementary Fig. 11A, C. Western blot analysis also verified this result using a specific anti-SGLT2 antibody (Fig. 5B, C). We also analyzed the mRNA level of SGLT2 in the kidneys of patients with lupus nephritis, hypertensive nephropathy and IgA nephropathy using the GEO database. The results indicated that SGLT2 mRNA level was significantly decreased in these nephropathic patients compared to healthy subjects (Fig. 5D). The SGLT2 is responsible for the tubular reabsorption of filtered glucose from the kidney into the bloodstream. The chemical structure of 1,5-AG is similar to that of glucose. Importantly, administration of *B. fragilis* significantly upregulated the UUO-induced decrease in mRNA and protein levels of the SGLT2 (Fig. 5B, C, E). *B. fragilis* transports more 1,5-AG from the kidney into the bloodstream possibly owing to upregulation of SGLT2 expression.

### SGLT2 is responsible for renal reabsorption of 1,5-AG

To explore the binding of 1,5-AG to SGLT2, in silico molecular docking and molecular dynamics (MD) simulation were performed. The root mean square deviation (RMSD) of protein backbone atoms and 1,5-AG during the MD simulation is shown in Fig. 5F. The RMSD of SGLT2 increased to 8 Å in 100 ns and then equilibrated at approximately 8 Å. 1,5-AG was stably bound at the pocket during all of the MD simulations. The binding mode of 1,5-AG to SGLT2 was determined through MD simulation. As shown in Fig. 5G, H, 1,5-AG formed five hydrogen bonds with SGLT2 by interacting with Lys321, Glu99, Phe98, Gln457 and Trp291. The binding energy calculated through the MM-PBSA method was −19.73 kcal mol^−1^. These results indicated that 1,5-AG might bind to SGLT2. Empagliflozin is an inhibitor of SGLT2 and works by increasing sugar loss in urine. We found that administration of empagliflozin significantly decreased the serum concentration of 1,5-AG compared to the control mice (Fig. 5I). Next, cellular uptake experiments of 1,5-AG were performed in stably SGLT2-transfected HEK293 versus wild-type (WT) cells. We found that the uptake of 1,5-AG in HEK293 cells transfected with SGLT2 was 1.7-fold higher than that in WT cells (Fig. 5J), suggesting that 1,5-AG was the substrate of SGLT2. Taken together, these results demonstrate that SGLT2 is responsible for renal reabsorption of 1,5-AG.

### Madecassoside ameliorates renal fibrosis in a gut microbiota–dependent manner

**Fig. 6:**
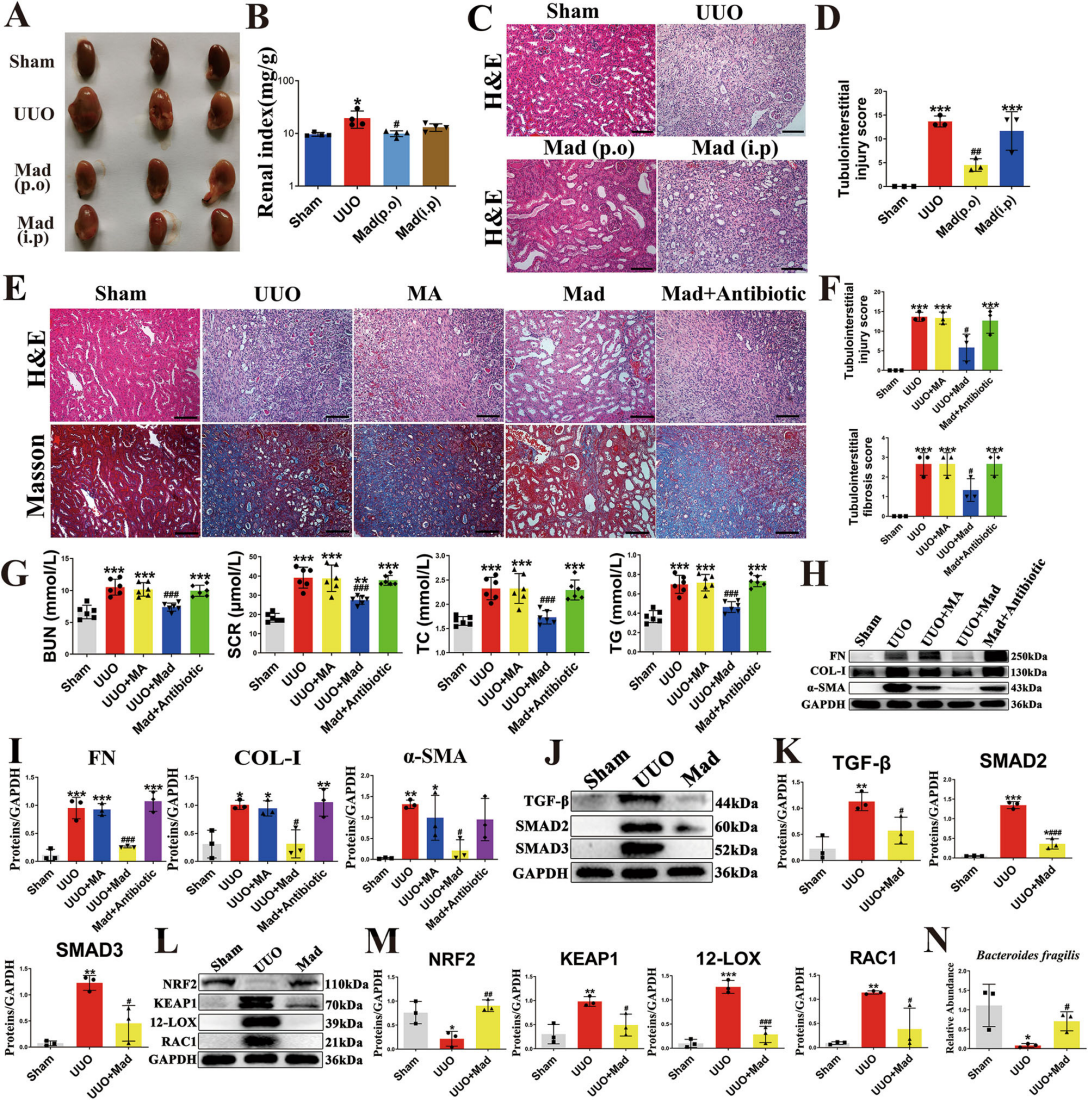
The anti-fibrotic effects of Mad in UUO model. **A** Picture of left kidneys of mice with different treatments (*n* = 3). **B** The renal index (mg/g) (*n* = 3). *p = 0.0140 for B: Sham vs. UUO, *p*  > 0.9999 for B: Sham vs. Mad (p.o), *p* = 0.7027 for B: Sham vs. Mad (i.p), ^#^*p* = 0.0175 for B: UUO vs. Mad (p.o), *p* = 0.1476 for B: UUO vs. Mad (i.p). **C** Representative photomicrographs of the H&E staining from left kidneys of Sham, UUO, Mad (p.o.) or Mad (i.p.)-treated UUO mice. **D** Bar graphs depict renal injury scores based on H&E staining (*n* = 3). ***p = 0.0003 for injury score: Sham vs. UUO, *p* = 0.1712 for injury score: Sham vs. Mad (p.o), ***p = 0.0010 for injury score: Sham vs. Mad (i.p), ^##^*p* = 0.0047 for injury score: UUO vs. Mad (p.o), *p* = 0.8302 for injury score: UUO vs. Mad (i.p). **E** Representative photomicrographs of the H&E staining and Masson’s trichrome staining from left kidneys of Sham, UUO, UUO + MA, UUO + Mad (p.o.), UUO + Mad + Antibiotic mice (H&E and Masson’s staining; scale bar, 100 μm; magnification, ×200). **F** Bar graphs depict renal injury scores and renal interstitial fibrosis scores based on H&E staining or Masson’s trichrome staining (*n* = 3). ***p = 0.0002 for injury score: Sham vs. UUO, ***p = 0.0002 for injury score: Sham vs. UUO + MA, *p* = 0.0692 for injury score: Sham vs. UUO + Mad, ***p = 0.0003 for injury score: Sham vs. Mad+Antibiotic, *p*  > 0.9999 for injury score: UUO vs. UUO + MA, ^#^*p* = 0.0119 for injury score: UUO vs. UUO + Mad, *p* = 0.9984 for injury score: UUO vs. Mad+Antibiotic; ***p = 0.0006 for fibrosis score: Sham vs. UUO, ***p = 0.0006 for fibrosis score: Sham vs. UUO + MA, *p* = 0.0687 for fibrosis score: Sham vs. UUO + Mad, ***p = 0.0006 for fibrosis score: Sham vs. Mad+Antibiotic, *p*  > 0.9999 for fibrosis score: UUO vs. UUO + MA, ^#^*p* = 0.0101 for fibrosis score: UUO vs. UUO + Mad, *p*  > 0.9999 for fibrosis score: UUO vs. Mad+Antibiotic. **G** Biochemical parameters including BUN, Scr, TC, TG in each of mice (*n* = 6). ***p  < 0.0001 for BUN: Sham vs. UUO, ***p < 0.0001 for BUN: Sham vs. UUO + MA, *p* = 0.7803 for BUN: Sham vs. UUO + Mad, ***p < 0.0001 for BUN: Sham vs. Mad+Antibiotic, *p* = 0.9949 for BUN: UUO vs. UUO + MA, ^###^*p*  < 0.0001 for BUN: UUO vs. UUO + Mad, *p* = 0.9366 for BUN: UUO vs. Mad+Antibiotic; ***p  < 0.0001 for SCR: Sham vs. UUO, ***p < 0.0001 for SCR: Sham vs. UUO + MA, **p = 0.0074 for SCR: Sham vs. UUO + Mad, ***p < 0.0001 for SCR: Sham vs. Mad+Antibiotic, *p*  > 0.9999 for SCR: UUO vs. UUO + MA, ^###^*p* = 0.0005 for SCR: UUO vs. UUO + Mad, *p* = 0.9982 for SCR: UUO vs. Mad+Antibiotic; ***p  < 0.0001 for TC: Sham vs. UUO, ***p < 0.0001 for TC: Sham vs. UUO + MA, *p* = 0.9957 for TC: Sham vs. UUO + Mad, ***p = 0.0001 for TC: Sham vs. Mad+Antibiotic, *p*  > 0.9999 for TC: UUO vs. UUO + MA, ^###^*p* = 0.0003 for TC: UUO vs. UUO + Mad, *p* > 0.9999 for TC: UUO vs. Mad+Antibiotic; ***p  < 0.0001 for TG: Sham vs. UUO, ***p < 0.0001 for TG: Sham vs. UUO + MA, *p* = 0.1726 for TG: Sham vs. UUO + Mad, ***p < 0.0001 for TG: Sham vs. Mad+Antibiotic, *p* = 0.9997 for TG: UUO vs. UUO + MA, ^###^*p*  < 0.0001 for TG: UUO vs. UUO + Mad, *p* = 0.9810 for TG: UUO vs. Mad+Antibiotic. **H** Kidney expression of FN, Col I and α-SMA from all groups, assayed by Western blot. **I** Quantitative analysis of Fig. 6H (*n* = 3). ***p = 0.0001 for FN: Sham vs. UUO, ***p = 0.0002 for FN: Sham vs. UUO + MA, *p* = 0.8340 for FN: Sham vs. UUO + Mad, ***p < 0.0001 for FN: Sham vs. Mad+Antibiotic, *p*  > 0.9999 for FN: UUO vs. UUO + MA, ^###^*p* = 0.0006 for FN: UUO vs. UUO + Mad, *p* = 0.9149 for FN: UUO vs. Mad+Antibiotic; *p = 0.0133 for COL-1: Sham vs. UUO, *p = 0.0240 for COL-1: Sham vs. UUO + MA, *p*  > 0.9999 for COL-1: Sham vs. UUO + Mad, **p = 0.0086 for COL-1: Sham vs. Mad+Antibiotic, *p*  > 0.9999 for COL-1: UUO vs. UUO + MA, ^#^*p* = 0.0139 for COL-1: UUO vs. UUO + Mad, *p* > 0.9999 for COL-1: UUO vs. Mad+Antibiotic; **p = 0.0079 for α-SMA: Sham vs. UUO, *p = 0.0480 for α-SMA: Sham vs. UUO + MA, *p* = 0.9946 for α-SMA: Sham vs. UUO + Mad, *p* = 0.0607 for α-SMA: Sham vs. Mad+Antibiotic, *p* = 0.9054 for α-SMA: UUO vs. UUO + MA, ^#^*p* = 0.0223 for α-SMA: UUO vs. UUO + Mad, *p* = 0.8440 for α-SMA: UUO vs. Mad+Antibiotic. **J** Kidney expression of TGF-β/Smad signaling pathway from Sham, UUO, Mad-treated UUO mice, assayed by Western blot. **K** Quantitative analysis of Fig. 6J (*n* = 3). **p = 0.0050 for TGF-β: Sham vs. UUO, ^#^*p* = 0.0428 for TGF-β: UUO vs. UUO + Mad; ***p  < 0.0001 for SMAD2: Sham vs. UUO, *p = 0.0173 for SMAD2: Sham vs. UUO + Mad, ^###^*p*  < 0.0001 for SMAD2: UUO vs. UUO + Mad; **p = 0.0018 for SMAD3: Sham vs. UUO, *p* = 0.2011 for SMAD3: Sham vs. UUO + Mad, ^#^*p* = 0.0135 for SMAD3: UUO vs. UUO^+^Mad. **L** Representative Western blot of Nrf2, keap1, 12-LOX, Rac-1. **M** Quantitative analysis of Fig. 6L (*n* = 3). *p = 0.0271 for NRF2: Sham vs. UUO, *p* = 0.7328 for NRF2: Sham vs. UUO + Mad, ^##^*p* = 0.0091 for NRF2: UUO vs. UUO + Mad; **p = 0.0083 for KEAP1: Sham vs. UUO, ^#^*p* = 0.0351 for KEAP1: UUO vs. UUO + Mad; ***p = 0.0001 for 12-LOX: Sham vs. UUO, *p* = 0.3690 for 12-LOX: Sham vs. UUO + Mad, ^###^*p* = 0.0003 for 12-LOX: UUO vs. UUO + Mad; **p = 0.0065 for RAC1: Sham vs. UUO, *p* = 0.4954 for RAC1^:^ Sham vs. UUO + Mad, ^#^*p* = 0.0300 for RAC1: UUO vs. UUO + Mad. **N** The relative abundance of *B. fragilis* in the sham, UUO and UUO + Mad groups measured by qPCR (*n* = 3). *p = 0.0309 for N: Sham vs. UUO, ^#^*p* = 0.0122 for N: UUO vs. UUO + Mad. Data are presented as mean ± SD. Comparison in N were performed with a two-tailed Student’s t test. Comparisons in **B**, **D**, **F**, **G**, **I**, **K**, **M** and **N** were compared using One-Way ANOVA followed by Sidak’s multiple comparisons test. ^*^P < 0.05, ^**^P < 0.01, ^***^P < 0.001 (compared with sham group), ^#^*P* < 0.05, ^##^*P* < 0.01, ^###^*P* < 0.001 (compared with UUO group). Individual data points are independent biological replicates unless otherwise stated.

Previous studies have shown that herbal medicines are able of maintaining intestinal flora homeostasis^30–32^. We have demonstrated that *B. fragilis* ameliorates renal fibrosis. Thus, we hypothesized that, active components from herbs could attenuate renal fibrosis via the action of *B. fragilis*. To this end, we assessed the growth-modulating effect of 14 active components associated with CKD on *B. fragilis* in vitro (Supplementary Fig. 12). The results indicated that only madecassoside (Mad) promoted the growth of *B. fragilis*. As shown in Fig. 6A, B, oral administration of Mad improved renal morphology and the renal index. Oral administration of Mad improved renal histology (Fig. 6C, D). Moreover, oral administration of Mad improved renal function in the UUO mice (Fig. 6G). However, Mad had little renoprotective effect in the UUO mice when administered by intraperitoneally (i.p.), which suggests that p.o. Mad does not exert its anti-renal fibrotic effect by direct absorption into the blood stream, but the intestinal tract might be its primary site of action (Fig. 6A–D). When Mad is administered p.o., it is eventually hydrolyzed to madecassic acid (MA) by intestinal flora^33^. We found that MA did not improve renal fibrosis, renal histology and renal function in the UUO-mice (Fig. 6E–I). To investigate whether the renoprotective effects of Mad are dependent on the presence of gut microbiota, we treated the UUO mice with a cocktail of antibiotics including ampicillin, gentamicin, neomycin, metronidazole and vancomycin. We found that when the gut microbiota was suppressed by the antibiotics cocktail, the renoprotective effects of Mad were abolished (Fig. 6E–I). Moreover, Mad inhibited oxidative stress and activation of the TGF-β/Smad signaling pathway in the UUO mice (Fig. 6J–M, Supplementary Fig. 5C). In addition, oral administration of Mad (80 mg/kg) did not affect the morphology of colon tissue (Supplementary Fig. 13A, B). Taken together, these results indicate that Mad protects mice against renal fibrosis in a gut microbiota–dependent manner. To confirm the growth-promoting effect of Mad on *B. fragilis* in vivo, we examined the abundance of *B. fragilis* in the sham, UUO- and Mad-treated mice by qPCR (Fig. 6N). The results indicated that treatment with Mad could restore the decreased *B. fragilis* level in the UUO mice.

### *B. fragilis*, 1,5-AG and Mad ameliorate renal fibrosis in adenine model

**Fig. 7:**
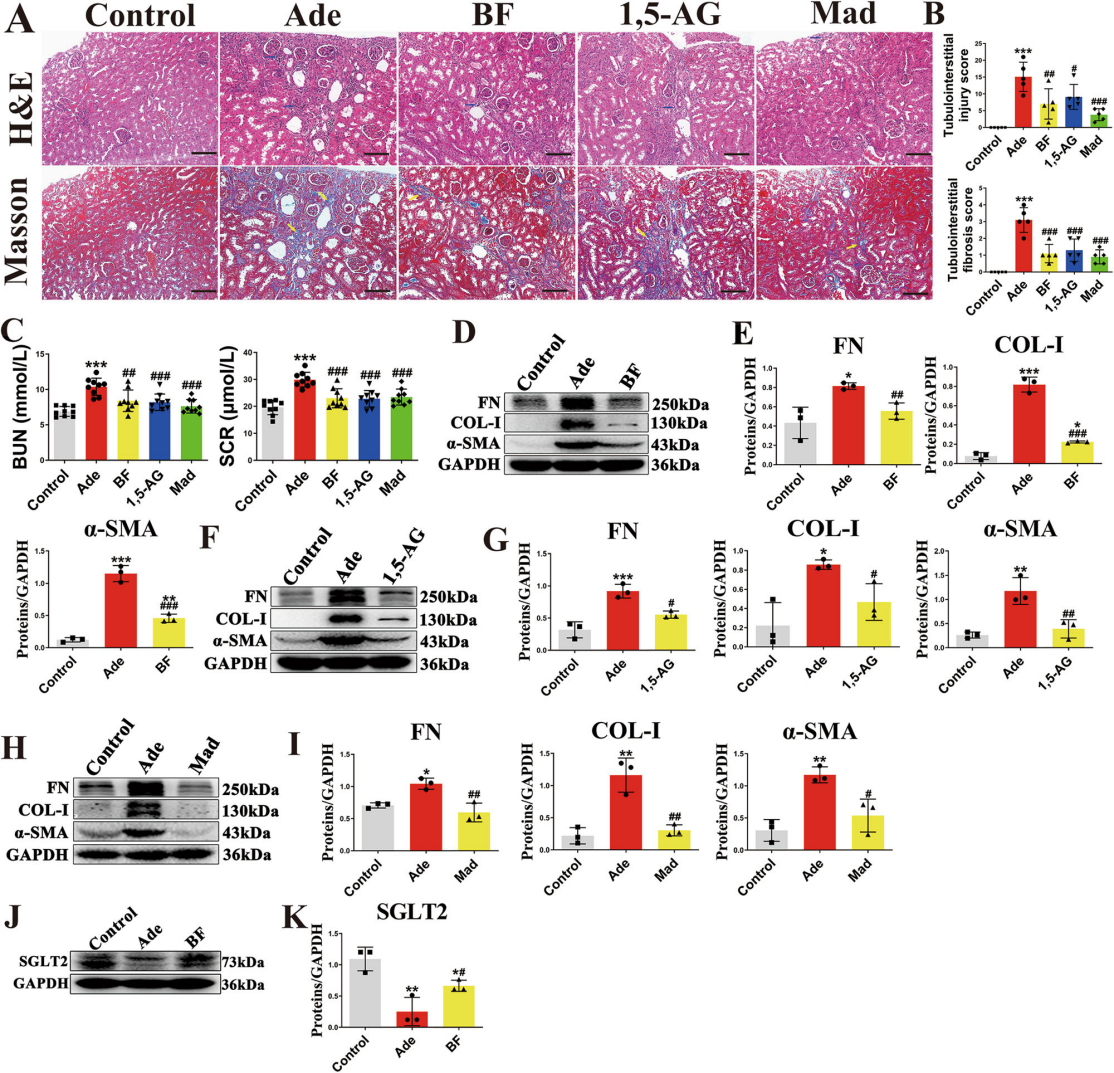
The anti-fibrotic effects of *B fragilis*, 1,5-AG and Mad in the adenine model. **A** Representative photomicrographs of the H&E staining and Masson’s trichrome staining from left kidneys of sham, adenine, *B. fragilis*-treated UUO mice, 1,5-AG-treated adenine mice and Mad-treated adenine mice (H&E and Masson’s staining; scale bar, 100 μm, magnification, ×200). **B** Bar graphs depict renal injury scores and renal interstitial fibrosis scores based on H&E staining or Masson’s trichrome staining (*n* = 5). ***p  < 0.0001 for injury score: Control vs. Ade, ^##^*p* = 0.0045 for injury score: Ade vs. BF, ^#^*p* = 0.0421 for injury score: Ade vs. 1,5-AG, ^###^*p* = 0.0001 for injury score: Ade vs. Mad; ***p  < 0.0001 for fibrosis score: Control vs. Ade, ^###^*p*  < 0.0001 for fibrosis score: Ade vs. BF, ^###^*p* = 0.0002 for fibrosis score: Ade vs. 1,5-AG, ^###^*p* < 0.0001 for fibrosis score: Ade vs. Mad. (C) Biochemical parameters including blood urea nitrogen (BUN), serum creatinine (Scr) (n=9). ***p  < 0.0001 for BUN: Control vs. Ade, ^##^*p* = 0.0029 for BUN: Ade vs. BF, ^###^*p* = 0.0009 for BUN^:^ Ade vs. 1,5-AG, ^###^*p* < 0.0001 for BUN: Ade vs. Mad; ***p  < 0.0001 for SCR: Control vs. Ade, ^###^*p*  < 0.0001 for SCR: Ade vs. BF, ^###^*p*  < 0.0001 for SCR: Ade vs. 1,5-AG, ^###^*p* ^=^ 0.0002 for SCR: Ade vs. Mad. (D) Kidney expression of FN, Col I and α-SMA from Sham, UUO and *B. fragilis*-treated adenine mice, assayed by Western blot. **E** Quantitative analysis of Fig. 7D (*n* = 3). *p = 0.0166 for FN: Control vs. Ade, ^##^*p* = 0.0080 for FN: Ade vs. BF, Comparison in FN were performed with a two-tailed Student’s t test^;^ ***p  < 0.0001 for COL-1: Control vs. Ade, *p = 0.0335 for COL-1: Control vs. BF, ^###^*p*  < 0.0001 for COL-1: Ade vs. BF; ***p  < 0.0001 for α-SMA: Control vs. Ade, **p = 0.0082 for α-SMA: Control vs. BF, ^###^*p* = 0.0002 for α-SMA: Ade vs. BF. **F** Kidney expression of FN, Col I and α-SMA from sham, adenine and 1,5-AG-treated adenine mice, assayed by Western blot. **G** Quantitative analysis of Fig. 7F (n = 3). ***p = 0.0010 for FN: Control vs. Ade, *p* = 0.0822 for FN: Control vs. 1,5-AG, ^#^*p* = 0.0128 for FN: Ade vs. 1,5-AG; *p = 0.0108 for COL-1: Control vs. Ade, ^#^*p* = 0.0266 for COL-1: Ade vs. 1,5-AG, Comparison in COL^-^1 were performed with a two-tailed Student’s t test; **p = 0.0040 for α-SMA^:^ Control vs. Ade, *p* = 0.8409 for α-SMA: Control vs. 1,5-AG, ^##^*p* = 0.0085 for α-SMA: Ade vs. 1,5-AG. (H) Kidney expression of FN, Col I and α-SMA from control, adenine and Mad-treated adenine mice, assayed by Western blot. **I** Quantitative analysis of Fig. 7H (n=3). *p = 0.0191 for FN: Control vs. Ade, *p* = 0.5363 for FN: Control vs. Mad, ^##^*p* = 0.0048 for FN: Ade vs. Mad; **p = 0.0018 for COL-1: Control vs. Ade, *p* = 0.9225 for COL-1: Control vs. Mad, ^##^*p* = 0.0030 for COL-1: Ade vs. Mad; **p = 0.0044 for α-SMA: Control vs. Ade, *p* = 0.4676 for α-SMA: Control vs. Mad, ^#^*p* = 0.0198 for ^α^-SMA: Ade vs. Mad. **J** Western blot shows SGLT2 protein expression in kidneys from control, adenine and adenine + BF groups. **K** Quantitative analysis of Fig. 7J (*n* = 3). **p = 0.0078 for K: Control vs. Ade, *p = 0.0428 for K: Control vs. BF, ^#^*p* = 0.0238 for K: Ade vs. BF, Comparison in K were performed with a two-tailed Student’s t test. Data are presented as mean ± SD. Com*p*arisons in **B**, **C**, **E**, **G** and **I** were compared using One-Way ANOVA followed by Sidak’s multiple comparisons test. ^*^P < 0.05, ^**^P < 0.01, ^***^P < 0.001 (compared with control group), ^#^*P* < 0.05, ^##^*P* < 0.01, ^###^*P* < 0.001 (compared with adenine group). Individual data points are independent biological replicates unless otherwise stated.

Here, we confirmed the renoprotective effects of *B. fragilis*, 1,5-AG and Mad using an adenine mouse model. H&E and Masson staining showed that tubular dilatation, tubular atrophy, and widening of the interstitial space with severe inflammatory cell infiltration were attenuated by administration of *B. fragilis*, 1,5-AG or Mad in adenine-treated mice (Fig. 7A, B). Treatment with *B. fragilis*, 1,5-AG or Mad significantly attenuated the increase in BUN and Scr levels, suggesting that *B. fragilis*, 1,5-AG or Mad improved renal function in the adenine-induced CKD mice (Fig. 7C). Adenine-treated mice that received *B. fragilis*, 1,5-AG or Mad showed significant reductions in the levels of several pro-fibrotic markers, including the pro-fibrotic proteins collagen I, fibronectin and α-SMA (Fig. 7D–I). However, MA (80 mg/kg) did not improve renal fibrosis, histology or function in the adenine-induced CKD mice (Supplementary Fig. 14). Moreover, the adenine-induced CKD mice showed significantly decreased SGLT2, while *B. fragilis* significantly upregulated the decreased SGLT2 level (Fig. 7J, K). The fecal and serum levels of LPS were assessed in adenine-treated mice. The results indicated that fecal and serum LPS levels were higher in the adenine-treated group in relation to the controls (Supplementary Fig. 15A, B). Oral administration of live *B. fragilis* or 1,5-AG dramatically decreased the fecal and serum LPS concentrations in the adenine-treated mice (Supplementary Fig. 15A, B). We then examined the intestinal tight junction expression of occludin and ZO-1 in the adenine-induced CKD mice. Adenine stimulation obviously decreased the expressions of occludin and ZO-1 in comparison with the control group (Supplementary Fig. 15C, D). *B. fragilis* treatment improved the protein level of occludin and ZO-1 compared to the adenine group (Supplementary Fig. 15C, D).

## Discussion

Increasing evidence suggests that the gut microbiota plays a key role in the development of CKD. Quantitative and qualitative alterations in the gut microbiota have been noticed in patients with CKD and ESRD^10,34^. Previous studies suggest that prebiotics and probiotics play pivotal roles in maintaining a metabolically balanced gut microbiota and reducing progression of CKD and uremia-associated complications^34^. Here, we found that the abundance of *B. fragilis* is lower in the gut microbiome of patients with CKD and UUO-mice. To our knowledge, this is the first study to report that oral administration of live *B. fragilis* can protect mice against renal fibrosis. The anti-fibrotic effect of *B. fragilis* may be associated with the downregulation of LPS and upregulation of 1,5-AG, which inhibits inflammation, oxidative stress, and the TGF-β/Smad signaling pathway. Additionally, we report a new function of SGLT2 as a 1,5-AG transporter in the kidney. 1,5-AG reabsorption in the kidney is improved by upregulation of SGLT2 expression. In addition, we demonstrate that Mad functions as *B. fragilis* growth modulator in vitro and in vivo, and show that the oral administration of Mad is effective in the upregulation of 1,5-AG to prevent development of renal fibrosis.

Bacterial species belonging to the phyla *Bacteroidetes* and *Firmicutes* dominate the gut microbiota. Among the Bacteroides species, *B. fragilis* is an important obligate anaerobe that colonizes the mammalian lower gastrointestinal tract^35^. *B. fragilis* has been extensively studied and shown to be equally effective in preventing colitis and experimental allergic encephalomyelitis (EAE) in murine models^17,36^. It has been reported that PSA produced by *B. fragilis* induces an anti-inflammatory milieu involving the stimulation of interleukin-10-producing CD4 + Foxp3 + T-regulatory cells in the intestine, thereby reducing pathological gastrointestinal symptoms in a mouse model of colitis^37,38^. Kasper et al.^39^ demonstrated that *B. fragilis* can protect against neuroinflammation in mouse models of multiple sclerosis. In general, these studies raised the possibility that *B. fragilis* might be important for the establishment of beneficial intestinal microbiota and could be developed into a probiotic therapy. However, whether or not *B. fragilis* could protect against renal fibrosis has not been studied. In this work, we found that the *B. fragilis* treatment effectively alleviated the disruption of serum biochemistry, renal histopathology and attenuated renal fibrosis in UUO/Adenine mice. In addition, treatment with *B. fragilis* could protect the kidneys by inhibiting inflammation, oxidative stress and the TGF-β/Smad signaling pathway.

Lipopolysaccharide (LPS), a component of the outer membrane of gram-negative bacteria, is a potent ligand for toll-like receptor 4 (TLR4), leading to the canonical activation of NF-κB and the associated expression of pro-inflammatory mediators, such as tumor necrosis factor α (TNFα) and interleukin-6 (IL-6)^40^. It is important to note that oral administration of *B. vulgatus* and *B. dorei* dramatically decreased colon LPS concentrations and offered protection against atherosclerosis. However, the effect of *B. fragilis* on LPS concentrations in UUO and adenine mice was not known until now. In the present study, we found that fecal and serum LPS levels were higher in CKD patients with a lower abundance of *B. fragilis*. Oral administration of live *B. fragilis* dramatically decreased the fecal and serum LPS concentrations and protected mice against inflammation. We examined the intestinal tight junction expression of occludin and ZO-1 in adenine-induced CKD mice. Adenine stimulation obviously decreased the production of occludin and ZO-1 in comparison to the control group. *B. fragilis* treatment improved the protein levels of occludin and ZO-1 compared to the levels in adenine-induced CKD mice. These results indicated that adenine-induced CKD mice showed breakdown of the gut epithelial barrier, which led to high levels of LPS in the serum. Moreover, the decreased LPS level in feces indicated that *B. fragilis* dramatically decreased the production of LPS

1,5-AG, a naturally occurring 1-deoxy form of glucose, is derived primarily from dietary sources^41^. Normally, in the kidneys, 1,5-AG is filtered and completely reabsorbed^42^. However, with elevated serum glucose concentrations, glucose is not completely reabsorbed by the kidney, and serum 1,5-AG falls due to competitive inhibition of renal tubular reabsorption. As such, low 1,5-AG is a marker of hyperglycemia over a period of approximately 1–2 weeks^43,44^. A global, untargeted metabolomics study discovered that the level of 1,5-AG, out of 204 metabolites examined, was an independent risk factor for chronic kidney disease in the Atherosclerosis Risk in Communities Study^45^. Rebholz et al. found that low 1,5-AG levels are associated with higher risk of incident ESRD independent of baseline kidney function^26^. In this study, we also confirmed the significantly decreased serum level of 1,5-AG in patients with CKD compared to controls. Since 1,5-AG is 1-deoxy form of glucose, we speculated that SGLT2 acts as a 1,5-AG transporter in the kidneys. It was reported that the expression of glucose transporters was decreased in nephrectomized rats^46^. As renal impairment or tubular damage progresses, it is possible that reabsorption of 1,5-AG would be reduced as a result of decreases in SGLT2 number and aggravating damage of glucose cotransporters, which might be responsible for the reduction of the serum level of 1,5-AG in patients with CKD. Thus, we examined the mRNA level of SGLT2 in kidneys from sham, and UUO-mice. The results indicated that SGLT2 mRNA level was significantly decreased in UUO mice, showing a trend similar to 1,5-AG. Furthermore, we demonstrated that administration of *B. fragilis* significantly upregulated the UUO-induced decrease in SGLT2 expression at the mRNA and protein levels. Molecular docking and MD simulation indicated the binding of 1,5-AG to SGLT2. Cellular uptake experiments suggested 1,5-AG as the substrate for SGLT2. Administration of empagliflozin significantly decreased the serum 1,5-AG levels. Our results demonstrated that *B. fragilis* treatment can upregulate the level of SGLT2 which contributes to renal reabsorption of 1,5-AG in UUO mice while replenishment of 1,5-AG improved renal dysfunction and attenuated inflammation and renal fibrosis.

TGR5, a cell membrane bile acid receptor, expression varied in multiple tissues^47^. Increasing evidence has reported the crucial role of TGR5 in many biological functions, including energy homeostasis and glucose metabolism. Activation of TGR5 was found to prevent kidney disease in obese and diabetic mice by inhibiting oxidative stress^48^ I In addition, recent studies have proved that TGR5 contributes significantly to ameliorating inflammation^49,50^. We have demonstrated here that, 1,5-AG is a TGR5 agonist and knockdown of TGR5 abolishes the anti-fibrotic effect of 1,5-AG in vitro, suggesting the anti-fibrotic effect of 1,5-AG might be dependent on TGR5.

In this study, we demonstrated that treatment with *B. fragilis* can ameliorate renal fibrosis. Thus, we speculated that certain small molecular compounds could attenuate renal fibrosis by remodeling the microbiota composition, especially *B. fragilis*. We assessed the growth-modulating effect of 14 active components from herbal medicines associated with CKD on *B. fragilis* in vitro. The results indicated that Mad significantly promotes the growth of *B. fragilis* in vitro. Mad is a pentacyclic triterpene isolated from *Centella asitica* (L.). A number of studies have suggested that this compound may exhibit anti‑inflammatory, antioxidant, anticancer and anti-pulmonary fibrotic effects^51–53^. As a triterpenoid saponin, orally administered Mad is extremely difficult to be absorbed^54^. When Mad is administered p.o. it is eventually hydrolyzed to MA which is the main product of intestinal metabolism. Thus, the level of Mad in plasma or tissue is much lower than the minimal effective concentration required for inhibition of lung fibroblasts^33,55^. We also examined whether Mad could promote the growth of *B. fragilis* and exert any anti-renal fibrotic effect in vivo. Our results suggest that oral administration of Mad, not MA, protected the mice against renal fibrosis in a gut microbiota–dependent manner. Moreover, treatment with Mad could restore the decreased *B. fragilis* abundance in the UUO mice, implying the in vivo growth-promoting effect of Mad on *B. fragilis*.

**Fig. 8:**
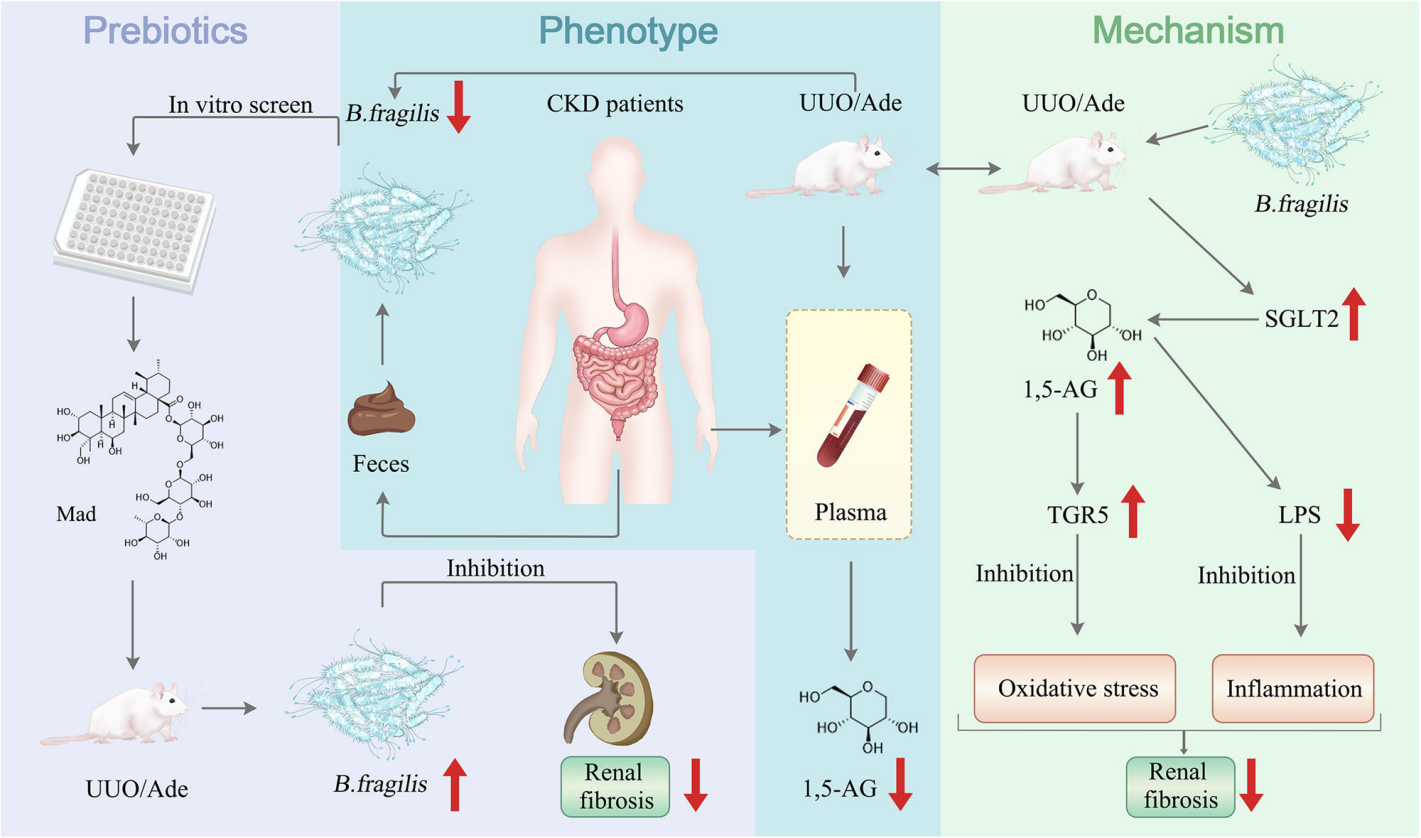
Proposed mechanism for the inhibition of renal fibrosis by *B. fragilis*. The relative abundance of *B. fragilis* is decreased in the feces of CKD patients and UUO mice. Oral administration of live *B. fragilis* attenuates renal fibrosis in UUO and adenine mice models. Increased LPS levels are decreased after *B. fragilis* administration. 1,5-AG, a substrate of SGLT2, increases after *B. fragilis* administration via enhancement of renal SGLT2 expression. 1,5-AG is an agonist of TGR5 that attenuates renal fibrosis by inhibiting oxidative stress and inflammation. Mad promotes *B. fragilis* growth and remarkably ameliorates renal fibrosis.

In summary, the results of our study show that oral administration of live *B. fragilis* could attenuate renal fibrosis and the anti-fibrotic effect of *B. fragilis* may be associated with the downregulation of LPS and upregulation of SGLT2 which contribute to renal reabsorption of 1,5-AG. 1,5-AG as an agonist of TGR5, attenuated renal fibrosis via inhibition of oxidative stress and inflammation. As a *B. fragilis* growth modulator, the small molecular compound Mad was effective in upregulating 1,5-AG to prevent the development of renal fibrosis. Our findings should be of value in modulating the microbiome as a useful therapeutic strategy for progressive renal fibrosis (Fig. 8).

## Methods

### Recruitment of patients with CKD and healthy controls

All procedures were approved by the medical ethics committee of the Affiliated Hospital of Nanjing University of Chinese Medicine and followed the tenets of the Declaration of Helsinki (2019NL-109-02). All subjects were informed of the use of their feces and blood, and written informed consent was obtained. The collection, preservation and processing of the fecal samples were conducted according to a previous protocol^56^. Feces from 10 CKD patients and 10 age- and sex-matched healthy subjects as microbiota discovery set were collected from the Department of Nephrology, Renmin Hospital of Wuhan University (Supplementary Table 3). Feces from 15 CKD patients and 15 age- and sex-matched healthy controls as microbiota validation set were enrolled from the Putuo People’s Hospital (Supplementary Table 4). Sera from 115 CKD patients and 113 age- and sex-matched healthy subjects for GC-MS based untargeted metabolomics were collected from the Affiliated Hospital of Nanjing University of Chinese Medicine (Supplementary Table 5). Sera from 110 CKD patients and 110 age- and sex-matched healthy subjects for external validation 1 using GC-MS based targeted metabolomics were collected from the Ningbo Hospital of Zhejiang University (Supplementary Table 6). Sera from 100 CKD patients and 100 age- and sex-matched healthy subjects for external validation 2 using LC-MS based targeted metabolomics were collected from the Putuo People’s Hospital (Supplementary Table 7). Slices of renal tissues from patients with category III immunoglobulin A nephropathy were provided by the Ningbo Hospital of Zhejiang University. Patients with acute kidney injury, liver disease, active vasculitis, gastrointestinal pathology or cancer were excluded from the study. As defined in international guidelines, CKD refers to a glomerular filtration rate (GFR) of less than 60 ml/min per 1.73 m^2^, or a marker of kidney damage, or both, for a duration of at least 3 months.

### Animal models

Animal experiments were conducted in accordance with the Guidelines for Animal Experimentation of China Pharmaceutical University (Nanjing, China), and the protocols were approved by the Animal Ethics Committee of this institution (No: 202002001). Mice were housed in pathogen-free and ventilated cages in a 12 h light/dark cycle, with room temperature at 25  ±  2 °C and humidity between 40 and 60%. All mice used in this work were male. Eight-week-old ICR male mice (18 to 22 g) were allowed free access to water and regular chow and their body weights taken every week. Mice models of renal fibrosis were established by known procedures for UUO^57^. After general anesthesia, complete UUO was carried out by double-ligation of the left ureter by 4–0 silk following a dorsal incision. The ureters of sham operated mice were exposed, but not ligated. For the adenine-induced mouse model, control group animals received saline daily (0.2 ml/100 g) by oral gavage for 6 weeks. The experimental groups received daily adenine injectable suspension of 80 mg/kg by oral gavage for 3 weeks^58^. Starting on 4^th^ week, the treatment groups received intervention for 3 weeks.

### Treatment strategies and antibiotic intervention

For UUO study, the experimental mice were randomly grouped as follows: (1) sham control; (2) UUO; (3) UUO + 1,5-AG (J&K Scientific, Bailingwei Technology, China; 100 mg/kg in saline administered i.p. for 7 days); (4) UUO + Mad (FeiYu Biotech Co Ltd, Nantong, China; 80 mg/kg in saline administered p.o. or i.p. daily for 14 days); (5) UUO + MA group (FeiYu Biotech Co Ltd, Nantong, China; 40 mg/kg given p.o. daily for 14 days); (6) UUO + Mad + antibiotics [YuanYe Bio-Technology Co Ltd (Shanghai, China); Ampicillin (5 mg/mL); Gentamicin (5 mg/mL); Neomycin (5 mg/mL); Metronidazole (5 mg/mL); Vancomycin (2.5 mg/mL); gavage mice with 200 μL of antibiotic mix].

For the adenine model, the experimental mice were randomly grouped as follows: (1) Control; (2) Adenine (J&K Scientific, Bailingwei Technology, China; 80 mg/kg by oral gavage); (3) Adenine + 1,5-AG (100 mg/kg in saline administered i.p. for 21 days); (4) Adenine + Mad (80 mg/kg in saline administered p.o. for 21 days); (5) Adenine + MA (80 mg/kg in saline administered p.o. for 21 days). (6) Control+ empagliflozin (GlpBio Technology, CA, USA; 10 mg/kg administered p.o. for 14 days). After the mice were sacrificed, their kidneys were harvested by surgical procedure and stored at −80 °C for further analysis. At least six animals were included in each group, and at least three independent experiments were performed. No animal was excluded from experiments unless for technical reasons.

### 16 S rDNA bacteria gene sequencing

DNA from human or mouse fecal samples was extracted using the E.Z.N.A. ®Stool DNA Kit (D4015, Omega, Inc., USA) according to manufacturer’s instructions. The V3-V4 region of the prokaryotic small-subunit (16 S) rRNA gene was amplified with primers 341 F (5′-CCTACGGGNGGCWGCAG-3′) and 805 R (5′-GACTACHVGGGTATCTAATCC-3′)^59^. The 5′ ends of the primers were tagged with specific barcodes per sample and sequencing universal primers. PCR amplification was performed in a total volume of 25 μL reaction mixture containing 25 ng of template DNA, 12.5 μL PCR Premix, 2.5 μL of each primer, and DEPC water to adjust the volume. The PCR products were confirmed with 2% agarose gel electrophoresis. The PCR products were purified by AMPure XT beads (Beckman Coulter Genomics, Danvers, MA, USA) and quantified by Qubit (Invitrogen, USA). The amplicon pools were prepared for sequencing and the size and quantity of the amplicon library were assessed on Agilent 2100 Bio-analyzer (Agilent, USA) and with the Library Quantification Kit for Illumina (Kapa Biosciences, Woburn, MA, USA), respectively.

The samples were sequenced on an Illumina NovaSeq platform by LC-Bio Technology Co., Ltd (Hang Zhou, Zhejiang Province, China) according to the manufacturer’s recommendations. Paired-end reads were merged using FLASH. Quality filtering on the raw reads were performed under specific filtering conditions to obtain the high-quality clean tags according to the Fqtrim (v0.94). Chimeric sequences were filtered using Vsearch software (v2.3.4). After de-replication using DADA2, we obtained feature table and feature sequence. Relative abundance was used in bacteria taxonomy, alpha diversity and beta diversity were analyzed by QIIME2 process, and figures were drawn by R (v4.1.0). The sequence alignment of species annotation was performed by QIIME2 plugin feature-classifier, and the alignment database was SILVA and NT-16S^60^. For 16 S rDNA bacteria gene sequencing of fecal samples in CKD patients and healthy subjects, the rarefaction number was 25134. Adjusted *p*-values less than 0.05 for phylum and genus were considered statistically significant in comparisons between CKD patients and healthy subjects.

### Fecal DNA extraction and quantification of the abundance of *B. fragilis*

Fecal DNA was extracted using the TIANamp Stool DNA Kit [TIANGEN Biotech (Beijing) Co. Ltd., DP328] according to the manufacturer’s protocol, and the concentration was measured by 24-well plate reader (BioTek, Winooski, VT, USA). qPCR assays were performed using the AceQ qPCR SYBR Green Master Mix (Vazyme Biotech Co. Ltd.) with primers that amplify the genes encoding 16S rRNA from *B. fragilis* and all bacteria (Supplementary Table 8) by the StepOne Real-Time PCR System (A&B, Waltham, MA, USA).

### Culture and preparation of *B. fragilis*

*B. fragilis* (NCTC 9343) was purchased from the National Collection of Type Cultures (NCTC) and cultivated in sterilized thioglycolate medium. An anaerobic chamber containing 10% CO_2_, 10% H_2_, and 80% N_2_ was used for all anaerobic microbiological works. Cultures were collected in log phase and diluted with sterile phosphate-buffered saline (PBS) to 2 × 10^8^ colony-forming units/ml for gavage. For the sham-controlled trials, *B. fragilis* were heat-killed at 121 °C (treatment duration, 15 min). The UUO-mice were gavaged daily with either live bacteria or sham (0.2 mL/10 g).

### *B. fragilis* growth modulators in vitro screening

The cultivated *B. fragilis* was seeded in 96-well plates under the initial OD_600_ value of approximately 0.1, and treated with 14 active natural products associated with CKD, including madecassoside (Mad), asiatic acid (AA), asiaticoside (Aad), madecassic acid (MA), ginsenoside Re, Rc, Rg1, Rh1, Rb1, artemisinin, emodin, astragaloside IV, dihydroartemisinin (DHA), and rhein at the final concentration of 100 µM. Then, the OD_600_ value was recorded after co-cultivation at different time-course of 0 h, 12 h, 24 h, 36 h, 48 h, 60 h, and 72 h using microplate reader. Modulators were screened through observation of the effects on the growth curve of *B. fragilis* in vitro.

### Cell culture and treatments

HK-2 cells (the Cell Resource Center, Peking Union Medical College) and HMC cells (FuHeng Biology, Shanghai) were cultured in DMEM/F-12 (C11330500BT, Gibco) and DMEM (01-052-1ACS, BI), respectively, supplemented with 10% FBS (04-001-1ACS, BI) and 1% penicillin/streptomycin (B540732, Sangon Biotech). The primary mouse renal tubular epithelial cells (PRTC cells) were isolated and cultured according to our previous report^61^. HK-2 cells were treated with 10 ng/mL recombinant human TGF-β1 protein (R&D system, USA), HMC cells were treated with 15 μg/ml LPS (L2630, Sigma, USA), while PRTC cells were treated with 10 ng/mL recombinant human TGF-β1 protein and 30 mM high glucose (A100188, Sangon Biotech). The concentration of 1,5-AG (BYOC-ALD-070-1g, Omicron Biochrmicals) for 24 h treatments of the HK-2, HMC and PRTC cells was 50 μM. The concentration of SBI-115 (TGR5 antagonist, HY-111534, MedChemExpress, Monmouth Junction, NJ, USA) for 48 h treatment of the PRTC cells was 10 μM.

### Blood parameter measurement, histology and Western blot analysis

Blood analyses were performed on an automatic biochemistry analyzer (Chemray 240, Redu Life Technology). Kidney tissue was fixed in 10% formaldehyde and then embedded in paraffin. Five µm-thick paraffin sections were cut. Sections were dewaxed and hydrated through graded alcohols and dipped in water, and then were stained with conventional H&E^62^. Western blot protocol^22^ is as follows: Protein concentration was measured by Enhanced BCA Protein Assay Kit (P0009, Beyotime, China). The 10–20 µl of total protein was fractionated by Polyacrylamide gel and transferred to a 0.45 μm hydrophobic PVDF transfer membrane (IPVH00010, Merk Millipore, Germany). After incubated for 2 h in 5% non-fat milk blocking buffer, the membranes were incubated overnight at 4 °C with primary antibody. The secondary antibodies of goat anti-rabbit (1:2000, #7074, CST, USA), horse anti-mouse (1:2000, #7076, CST, USA) were incubated with 2 h at room temperature.

The following antibodies were used: anti-collagen I (ab260043, Abcam, rabbit, dilution 1:1000 for WB), anti-fibronectin (ab2413, Abcam, rabbit, dilution 1:2000 for WB), anti-α-SMA (ab124964, Abcam, rabbit, dilution 1:5000 for WB), anti-GAPDH (HRP-60004, Proteintech, dilution 1:4000 for WB), anti-TGF-β1 (21898-1-AP, Proteintech, rabbit, dilution 1:1000 for WB), anti-Smad2 (#5339, CST, rabbit, dilution 1:1000 for WB), anti-Smad3 (#9523, CST, rabbit, dilution 1:1000 for WB), anti-12-LO (C-5) (sc-365194, Santa Cruz Biotechnology, mouse, dilution 1:100 for WB), anti-Rac1 (66122-1-Ig, Proteintech, mouse, dilution 1:1000 for WB), anti-Nrf2 (16396-1-AP, Proteintech, rabbit, dilution 1:1000 for WB), anti-Keap1 (10503-2-AP, Proteintech, rabbit, dilution 1:1000 for WB), anti-HO-1 (66743-1-Ig, Proteintech, mouse, dilution 1:1000 for WB), anti-SGLT2 (ab37296, Abcam, rabbit, dilution 1:1000 for WB), ZO-1(21773-1-AP, Proteintech, rabbit, dilution 1:2000 for WB), Occludin (66378-1-Ig, Proteintech, mouse, dilution 1:5000 for WB), anti-TGR5 (ab72608, Abcam, rabbit, dilution 1:1000 for WB and 1:100 for IHC), anti-IL-1β (ab254360, Abcam, rabbit, dilution 1:1000 for WB), anti-TNF-α (ab215188, Abcam, rabbit, diltion 1:1000 for WB), anti-IL-6 (ab259341, Abcam, rabbit, dilution 1:1000 for WB), anti-Vimetin (#5741, CST, rabbit, dilution 1:1000 for WB and 1:50 for IF), E-cadherin (#14472, CST, mouse, dilution 1:50 for IF), HRP-linked anti-rabbit IgG (#7074, CST, rabbit, dilution 1:2000 for WB), HRP-linked anti-mouse IgG (#7076, CST, mouse, dilution 1:2000 for WB).Semi-quantitative analysis of each protein was performed using ImageJ software (version 1.5), and the band densities were normalized to the band density of GAPDH.

### Small-interfering RNA (siRNA) and transient transfection

PRTC cells were plated in 6-well plates for 24 h before transfection. siRNA targeting TGR5 was acquired from Sangon Biotech. The sequences of TGR5-siRNA were as follows: sense: 5′-CUCUGUUAUCGCUCAUCUCAUTT-3′ and antisense: 5′-AUGAGAUGAGCGAUAACAGAGTT-3′. The siRNA was transfected using INTERFER transfection reagent according to the manufacturer’s protocol.

### Lentiviral and plasmid package and cell transfection

Plasmids encoding SLC5A2 vector (HBLV-h-SLC5A2-3xflag-PURO) and control vector (HBLV-PURO) were designed and lentivirus was provided by HANBIO (Shanghai, China). HEK293 cells (Stem Cell Bank, Chinese Academy of Sciences, GNHu 43) were transfected with SLC5A2 or control virus at a multiplicity of infection (MOI) of 2. The cells were then treated with 1 μg/ml puromycin for 14 days. The stably expressed SCL5A2 colonies were verified by Western blot. We found overexpression of SGLT2 in HEK293 cells was established (Supplementary Fig. 16).

### mRNA isolation and qRT-PCR

Total mRNA was extracted using a High Pure RNA Isolation Kit (RNAiso Plus, Takara Bio, Japan) according to the manufacturer’s instructions. Total RNA was reverse transcribed by a HiScript II QRT SuperMix for qPCR according to the manufacturer’s instructions (+gDNA wiper, R233-01, Vazyme, Nanjing, China). Quantitative real-time PCR (qRT-PCR) was carried out by the Step One System (A&B, Waltham, MA, USA) using AceQ qPCR SYBR Green Master Mix (High ROX Premixed, Q141-02, Vazyme Biotech Co. Ltd.). The mRNA levels of the genes were calculated by normalization to the levels of *Gapdh*. Primer sets for genes are presented in Supplementary Table 8.

### Immunofluorescence staining

For immunofluorescence of vimentin and E-Cadherin in the kidney tissues, the slides of the tissues were incubated with vimentin (#5741, CST, concentration, 1:50) or E-Cadherin (#14472, CST, concentration, 1:50) overnight at 4 °C in a humidified dark chamber, and then incubated with Alexa Fluor 555-labeled goat anti-rabbit IgG (H + L) (ab150078, Abcam, concentration, 1:200) or FITC-labeled goat anti-mouse IgG (H + L) (SA00003-1, Proteintech, concentration, 1:200) for 1 h at room temperature. Subsequently, they were washed with PBS and stained with DAPI for 10 min. The image was acquired by confocal laser scanning microscope (LSM700, Zeiss, Jena, Germany).

### Immunohistochemical staining

Sections (5 μm) of kidney tissues were obtained and deparaffinized in xylene, hydrated in graded ethanol solutions, and rinsed with tap water and distilled water. Then, endogenous peroxide activity was blocked by incubation in 0.3% hydrogen peroxide in methanol for 30 min. For antigen retrieval, the kidney tissue sections were incubated with 10 μmol/L citrate buffer solution (pH: 6.0) and boiled for 10–15 min. Subsequently, the sections were blocked with 10% normal goat serum for 1 h at room temperature and then incubated overnight at 4 °C with the TGR5 antibody (ab72608, Abcam, concentration, 1:75). After washing with PBS, the HRP-linked goat anti-rabbit IgG (AFIHC003, AiFang biology, no dilution) was added, and the sections were incubated at 37 °C for 1 h. Finally, the kidney tissue sections were exposed to diaminobenzidine peroxidase substrate for 5 min and counterstained with haematoxylin and eosin. Images of the sections were obtained using a Leica DMi8 fluorescence microscope (Leica, Germany).

### ELISA assay

Sera were collected from the blood of human and mice by centrifuging at 2000 × *g* for 10 min. For fecal samples preparation, feces were accurately weighed, and homogenized for 5 min in 9-fold volume of PBS. The supernatant was collected by centrifugation at 5000 × g for 10 min. For cell sample preparation, cells were collected using a sterile container, and diluted for 1 million/mL using PBS. Intracellular components were released by repeated freeze-thaw cycles. The supernatant was collected by centrifugation at 2000 × *g* for 10 min. For tissue sample preparation, the tissue samples were accurately weighted, and rapidly frozen with liquid nitrogen, then homogenized by grinders at 4 °C after melting. The supernatant was collected by centrifugation at 5000 × *g* for 10 min. After treatment, the levels of LPS, pro-inflammatory cytokines and cAMP were assayed. Human LPS (KT98561, MSKBIO, maximum concentration of standard: 960 ng/L) and mouse LPS (KT37561, MSKBIO, maximum concentration of standard: 640 pg/mL), IL-1β (KT21178, MSKBIO, maximum concentration of standard: 160 ng/L), IL-6 (KT99854, MSKBIO, maximum concentration of standard: 240 pg/mL), TNF-ɑ (KT99985, MSKBIO, maximum concentration of standard: 1600 ng/L) in the supernatants of serum and feces were quantified by ELISA. cAMP (E-EL-0056c, Elabscience, maximum concentration of standard:100 ng/L) in the supernatants of cells and renal samples were quantified by ELISA according to the manufacturer’s protocol.

### Mice serum collection

Mice were anesthetized with 10% urethane, and blood samples were obtained by carotid artery cannula on the 14th day. Blood was centrifuged at 2000 × *g* for 10 min and the sera were collected and stored at −80 °C.

### Human serum collection

All the blood samples were immediately centrifuged at 2000 × *g* for 10 min, and sera were transferred into clean Eppendorf tubes. The serum samples were stored at −80 °C.

### Serum sample preparation

Internal standard solutions (10 μL of myristic acid-1,2-^13^C_2_ in methanol, 1 mg/mL) were added to 200 μL of serum. The mixed solution was extracted with 600 μL of methanol and chloroform (3:1, *v*/*v*) and vortexed for 30 s. The mixture was stored at room temperature for 10 min and centrifuged at 15,000 × *g* for 10 min at 4 °C. The resulting supernatant (600 μL) was transferred to a sample vial for vacuum drying at room temperature. The residue was redissolved in 40 μL of a methoxyamine solution (15 mg/mL in pyridine) and vortexed for 1 min. An oximation reaction was performed at 37 °C for 1.5 h. Then, 80 μL of BSTFA (containing 1% TMCS) was added to the solution, and the solution was vortexed for 30 s. The sample was kept at 70 °C for 1 h and vortexed for 10 s. The supernatant was then transferred to a sample vial for GC-MS analysis^63^. Representative total ion chromatograms are presented in Supplementary Fig. 17.

### Reference standard (1, 5-AG) preparation

1, 5-AG standard solutions was diluted to different concentrations. The 600 μL solution was transferred to a sample vial for vacuum drying at room temperature. The residue was redissolved in 50 μL of a methoxyamine solution (15 mg/mL in pyridine) and vortexed for 1 min. An oximation reaction was performed at 37 °C for 1.5 h. Then, 50 μL of BSTFA (containing 1% TMCS) was added to the solution, and the solution was vortexed for 30 s. The sample was kept at 70 °C for 1 h and vortexed for 10 s. The supernatant was transferred to a sample vial for GC-MS analysis. The calculation curve is shown in Supplementary Fig. 18.

### GC-MS analysis

The samples were analyzed using an Agilent 7890 chromatograph coupled with a 5977B MS system (Agilent Technologies, Santa Clara, CA, USA). Separation was achieved on a DB-5 ms capillary column coated with 95% dimethyl 5% diphenyl polysiloxane (30 m× 0.25 mm i.d., 0.25-μm film). The initial GC oven temperature was set at 60 °C for 1 min, followed by a 10 °C/min oven temperature ramp to 325 °C, which was maintained for 10 min. The temperature of the inlet, transfer line, and ion source was set to 250, 290, and 250 °C, respectively. The injection volume for untargeted metabolomics was 1 μL with splitless. The injection volume for targeted metabolomics was 1 μL with splitless. Helium was used as the carrier gas with a constant flow rate of 0.87 mL/min. Measurements were made with electron impact ionization (70 eV) in full scan mode (m/z 50 − 650). Quantification analysis of 1,5-AG was performed with SIM mode with *m/z* 259.0^64^.

### GC-MS method assessment

For analytical method assessment, 50 µl each of all the samples were pooled to get a quality control sample (QC) that would be tested during the analysis. QC samples were analyzed five times at the beginning of the run and injected once after every 10 injections of the random sequenced samples. The precision and repeatability were validated by the duplicate analysis of six injections of the same QC sample and six parallel QC samples prepared using the same preparation and method, respectively. From this QC sample, extracted ion chromatographic peaks of five ions (7.72_72.1, 11.91_234.1, 17.07_259.1, 19.39_267.1, 27.68_369.1) were selected for method validation. The RSD of peak area and retention time were below 3.1% and 0.04% respectively and the reproducibility and precision were satisfactory for metabolomic analysis. The stability of samples was tested by analyzing two QC samples kept in autosampler at room temperature for 12, 24 and 72 h. The RSD of peak areas of the serum sample for metabolomic analyses was 2.0% to 5.1%.

### Data analysis

The raw mass spectrometry data were exported to data format (mzdata) files by Mass Hunter Workstation Software (version B.06.00, Agilent Technologies). Data pre-treatment procedures, such as nonlinear retention time alignment, peak discrimination, filtering, alignment, matching, and identification, were performed in XCMS package (Scripps Center for Metabolomics and Mass Spectrometry, La Jolla, California). The matrix result was reduced by replacing the missing values, and data with more than 20% missing values were removed. The resulting data set, including retention time, sample names and peak areas were introduced into the SIMCA-P 14.0 Software package (Umetrics, Umea, Sweden) for multivariate statistical analysis. The significance of each metabolite was analyzed by the Mann-Whitney-Wilcoxon test with false discovery rate (FDR) correction via Benjamini–Hochberg method. The discrimination of variables was identified by Orthogonal partial least-squared discriminant analysis (OPLS-DA). Adjusted *p*-values less than 0.05 were considered statistically significant. Differential metabolites were screened by those with variable importance in the projection (VIP) ≥ 1.0 obtained from OPLS-DA and adjusted *p*-values less than 0.05, where VIP indicates the contribution of each variable to group differences. Differential metabolites were identified by a library search (NIST and Fiehn) and confirmed by available references.

### Targeted metabolomics analysis of human serum samples using LC-MS

UltiMate® 3000 ultra-performance liquid chromatography system (DIONEX, Sunnyvale, CA, USA) equipped with an ACQUITY UPLC® BEH Amide (2.1 × 100 mm, 1.7 μm, Waters Co., Milford, MA, USA) was used to perform the chromatographic separation of 1, 5-AG. Mobile phase A was water containing10 mM ammonium acetate, while mobile phase B was acetonitrile and water (95: 5), containing 10 mM ammonium acetate. The mobile phase gradient was as follows: 0–1 min, 85 % B, 1–6 min, 85–10% B, 6–8 min, 10–85% B, 8–12 min, 85% B. The flow rate was 0.3 mL/min. The column oven temperature was set to 50 °C. TSQ VantageTM triple quadrupole mass spectrometer (Thermo Fisher Scientific Inc.) was used for the determination of 1, 5-AG. The ESI source was set as negative ion mode with the following parameters: spray voltage, 2.8 kV; capillary temperature, 300 °C; sheath gas flow rate, 45 arb; aux gas flow rate, 15 arb; vaporizer temperature, 300 °C. The MRM parameters of 1, 5-AG were as follows: Parent ion, 163; Product ion, 101; CE, 15; S-lens, 55. The MRM parameters of 1,5-AG-^13^C_6_ were as follows: Parent ion, 169; Product ion, 105; CE, 15; S-lens, 55. For sample preparation, an aliquot of 50 μL serum was mixed with 200 μL MeOH containing 5 μg/mL of 1,5-AG-^13^C_6_ in 1.5 mL polypropylene test tube. The mixture was then vortexed for 3 min and centrifuged at 13500 × *g* for 10 min to remove the precipitated protein. The 200 μL supernatant was transferred to another tube and evaporated to dryness by a vacuum drier. The dried residue was redissolved in 80 μL 50% MeOH and centrifuged at 13500 × *g* again for 10 min, and an aliquot of 2 μL supernatant was subjected to LC-MS system. The quantitation of 1,5-AG in the validation of center 2 was performed with reference to the corresponding isotope-labeled internal standard (20 μg/mL of 1,5-AG-^13^C_6_) according to previous report^65^.

### Label-free quantitative proteomics

Each mouse kidney tissue was ground into powder in nitrogen., then lysis buffer (0.1 M Tris-HCL, pH 7.5, 4% SDS, 0.1 M DTT) was added, and the mixture was sonicated with 20 cycles of pulses (30 s each on/off, 80% power; CosmoSonic II Ultra Sonicator). After heating for 10 min at 95 °C, the lysate was centrifuged at 16,000 × *g* for 20 min at room temperature. The protein concentrations were determined by measuring tryptophan fluorescence^66^. 100 μg protein was digested by the FASP method^67^. Each sample peptides were loaded onto a 20-cm column packed in-house with C_18_ 3 μM ReproSil particles (Dr. Maisch GmbH), with an EASY-nLC 1200 system (Thermo Fisher Scientific) coupled to the mass spectrometer (Q Exactive Plus, Thermo Fisher Scientific). Column temperature was maintained at 50 °C. Peptides were separated with a 120 min gradient at a flow rate of 300 nL/min. Each sample was detected twice. The raw data files were processed using software MaxQuant (http://www.maxquant.org.) version 1.6.2.10 with an FDR < 0.01 at the levels of proteins and peptides. The MS/MS spectra were searched against the Homo sapiens protein database in UniProt (January 2021). Bioinformatics analyses were carried out with R version 4.1.0 (https://www.r-project.org/) statistical computing software.

### Molecular docking and molecular dynamics (MD) simulations

The crystal structure of human SGLT2 was predicted by AlphaFold2 (PDB: AF-P31639-F1-model_v1)^68^. The crystal structure of human TGR5 was downloaded from the Protein Data Bank (PDB: 7BW0). Then, the binding of 1,5-AG to SGLT2 and TGR5, was performed using the AutoDock program^69^. The genetic algorithm was applied for conformational analysis. To assess the conformational space of 1,5-AG as completely as possible, we performed 100 individual genetic algorithm runs to generate 100 docked conformations. The size of the docking box was properly set to enclose the possible binding pocket. The protein structure was fixed during molecular docking. The docked protein-ligand complex structure was further relaxed through molecular dynamics (MD) simulation by using the Amber program^70^. The most populated structure during the 500 ns MD simulation was obtained via cluster analysis.

### Uptake study for 1,5-AG

Stably transfected HEK293 cells and control vector-transfected HEK293 cells were cultured in high glucose DMEM with 10% FBS, 1% penicillin-streptomycin, and seeded onto 10 cm dish and incubated at 37 °C, 5% CO_2_, and 95% humidity. Daily changes of medium were performed. At 48 h after plating, uptake experiments were conducted. Cells were first washed 3 times with prewarmed PBS (pH 7.4) and then were incubated with 4 mL of PBS containing 1,5-AG-^13^C_6_ at concentrations of 100 μM. Cells were incubated for 10 min at 37 °C. After incubation, uptake was stopped by aspirating the incubation solution and washing each well 3 times with ice PBS. The cells were lysed in 1 mL of 0.1 M NaOH for 30 min, and then neutralized by adding equal volumes of 0.1 M HCl. Some of the cell lysates were used as protein concentration determination by BCA method and the others were used for quantification by GC-MS above. The following Eqs. (1 and 2) were used to calculate the uptake rates (U) and the SGLT2 uptake ratio (UR). *A*_(analyte)lysate_ is the intensity of 1,5-AG-^13^C_6_ in cell lysates. *A*_(IS)lysate_ is the intensity of internal standard (myristic acid-1,2-^13^C_2_) in cell lysates. *P* is the protein concentration in cell lysates. *T* is the incubation time. U_HEK293-SGLT2_ is the uptake rate obtained in HEK293 stably transfected SGLT2. U_HEK293-MOCK_ is the uptake rate obtained in control vector-transfected HEK293. The uptake experiment was conducted according to our previous study^71^.

1 $$U=\frac{{A}_{({{{{{\rm{analyte}}}}}}){lysate}}}{{A}_{({{{{{\rm{IS}}}}}}){lysate}}\times P\times T}$$

2 $${UR}=\frac{{U}_{{{{{{\rm{HEK}}}}}}293-{{{{{\rm{SGLT}}}}}}2}}{{U}_{{{{{{\rm{HEK}}}}}}293-{{{{{\rm{MOCK}}}}}}}}$$

### Statistical analysis

Data are shown as means ± SD. Multigroup comparisons were performed using one-way ANOVA. Student’s t-test or Mann-Whitney-Wilcoxon test was used for comparisons between two groups. At least three independent experiments were performed. Analyses were performed with Prism Software (GraphPad Software 9.0). All results were considered statistically significant at *p*-value < 0.05.

### Reporting summary

Further information on research design is available in the Nature Research Reporting Summary linked to this article.

## Supplementary information

Supplementary information

Reporting Summary

## Data Availability

The mass spectrometry proteomics data have been deposited to the ProteomeXchange Consortium under accession code PXD029310. The data of 16S rDNA bacteria gene sequencing have been deposited to the SRA database in NCBI under accession code PRJNA772031. All the data supporting this study are available within the article, the Supplementary file, the Supplementary data file, and the Source data file, as indicated in the Reporting summary for this article. Source data for all quantifications are provided as a Source data file. Source data are provided in this paper.

## Source data

Source Data
