# Supplementary material for: The gut microbe Bacteroides fragilis ameliorates renal fibrosis in mice
Source: Nat Commun. 2022 Oct 14;13:6081. doi: 10.1038/s41467-022-33824-6 (PMC9568537; doi:10.1038/s41467-022-33824-6)
Supplement: Supplementary file 1 — Supplementary information [file 41467_2022_33824_MOESM1_ESM.doc]

**The gut microbe *Bacteroides fragilis* ameliorates renal fibrosis in mice**

Wei Zhou^a,1^, Wen-hui Wu^a,1^, Zi-lin Si ^a,1^, Hui-ling Liu^a^, Hanyu Wang^a^, Hong Jiang^a^, Ya-fang Liu^a^, Raphael N. Alolga^a^, Cheng Chen^b^, Shi-jia Liu^c^, Xue-yan Bian^d^, Jin-jun Shan^e^, Jing Li^f^, Ning-hua Tan^a,*^, Zhi-hao Zhang^a,*^

^a^ State Key Laboratory of Natural Medicines, Department of TCMs Pharmaceuticals, School of Traditional Chinese Pharmacy, China Pharmaceutical University, Nanjing 211198, China;

^b^ Department of Nephrology, Renmin Hospital of Wuhan University, Wuhan 430060, China;

^c^ Affiliated Hospital of Nanjing University of Chinese Medicine, Nanjing 210029, China;

^d^ Ningbo Hospital of Zhejiang University, Ningbo 315000, China;

^e^ Medical Metabolomics Center, Nanjing University of Chinese Medicine, Nanjing, 210023 China;

^f^ School of Life Science and Technology, China Pharmaceutical University, Nanjing 211198 China.

^1^ Wei Zhou, Wen-hui Wu and Zi-lin Si are the co-first authors.

Corresponding author:

Ning-hua Tan, PhD, Professor

School of Traditional Chinese Pharmacy, China Pharmaceutical University, Nanjing 211198, China, Tel: +86 25 86185772; E-mail: nhtan@cpu.edu.cn

Zhi-hao Zhang, PhD, Associate Professor

School of Traditional Chinese Pharmacy, China Pharmaceutical University, Nanjing 211198, China, Tel: +86 25 86185772; E-mail: zzh-198518@163.com

**I. Supplementary tables**

**Supplementary Table 1：**Fourteen differential metabolites identified from Sham and UUO mice using GC-MS based untargeted metabolomics.

**Supplementary Table 2：**Twenty-three differential metabolites identified from healthy subjects *vs* CKD patients using GC-MS based untargeted metabolomics.

**Supplementary Table 3:** Baseline characteristics of feces from 10 CKD patients and 10 age- and sex-matched healthy subjects.

**Supplementary Table 4:** Baseline characteristics of feces from 15 CKD patients and 15 age- and sex-matched healthy subjects.

**Supplementary Table 5:** Baseline characteristics of sera from 115 CKD patients and 113 age- and sex-matched healthy subjects.

**Supplementary Table 6:** Baseline characteristics of sera from 110 CKD patients and 110 age- and sex-matched healthy subjects.

**Supplementary Table 7:** Baseline characteristics of sera from 100 CKD patients and 100 age- and sex-matched healthy subjects.

**Supplementary Table 8.** Primer pairs for real-time qPCR.

**II. Supplementary figures**

Supplementary Fig. 1. 16S rDNA bacteria gene sequencing of feces in human.

Supplementary Fig. 2. Relative abundance of *B. fragilis* in UUO model.

Supplementary Fig. 3. The anti-inflammatory effects of *B fragilis*.

Supplementary Fig. 4. qPCR analysis of TGF-β/Smad pathway in UUO model.

Supplementary Fig. 5. qPCR analysis of oxidative stress in UUO model.

**Supplementary Fig. 6.** PCA in the indicated groups.

Supplementary Fig. 7. GC-MS based metabolomics analysis of serum samples from healthy control and CKD groups.

**Supplementary Fig. 8.** The anti-inflammatory effects of 1,5-AG in UUO and Adenine models.

**Supplementary Fig. 9.** The anti-fibrotic and anti-inflammatory effects of 1,5-AG *in vitro*.

**Supplementary Fig. 10.** Effect of different siRNAs on expression of TGR5 in primary mouse renal tubular epithelial cells.

**Supplementary Fig. 11.** The proteomics analyses of kidney tissues in UUO model.

**Supplementary Fig. 12.** Growth-modulating effect of 14 active components associated with CKD on *B. fragilis* *in vitro* (n=4)*.*

**Supplementary Fig. 13.** Representative photomicrographs of the H&E staining from colon tissue of all groups in UUO model.

**Supplementary Fig. 14.** The effect of madecassic acid (MA) on renal fibrosis using adenine model.

**Supplementary Fig. 15.** The anti-inflammatory effects of 1,5-AG using adenine model.

**Supplementary Fig. 16.** Expression of SGLT2 in HEK-293 cells stably transfected SCL5A2 colony.

Supplementary Fig. 17. Representative chromatograms of GC-MS.

Supplementary Fig. 18. Calibration curve and representative chromatograms of 1,5-AG by GC-MS.

**Supplementary Table 1：**Fourteen differential metabolites identified from Sham and UUO mice using GC-MS based untargeted metabolomics

| **Primary ID** | **UUO vs Sham** | | | | | |
| --- | --- | --- | --- | --- | --- | --- |
|  | **m/z** | **Rt(min)** | **^a^ FC (UUO/Sham)** | **^b^ VIP** | **^c^ U test** | **^d^ FDR adjusted U test** |
| Lactic acid ***** | 67 | 7.09 | 1.41 | 1.04 | 1.5×10^-4^ | 2.4×10^-3^ |
| Valine ***** | 156 | 7.61 | 1.51 | 1.07 | 4.7×10^-2^ | 4.8×10^-2^ |
| Phosphoric acid ***** | 214 | 9.56 | 2.39 | 1.01 | 3.3×10^-2^ | 3.8×10^-2^ |
| Urea ***^i^** | 189 | 9.88 | 0.60 | 1.27 | 4.1×10^-2^ | 4.4×10^-2^ |
| Glycerol ***** | 129 | 10.12 | 0.68 | 1.18 | 1.6×10^-2^ | 2.3×10^-2^ |
| Glutamic acid ***** | 158 | 13.43 | 1.59 | 1.25 | 2.9×10^-2^ | 3.6×10^-2^ |
| α-Ketoglutaric acid ***** | 198 | 14.05 | 2.08 | 1.33 | 5.5×10^-3^ | 1.5×10^-2^ |
| Mannose **^#^** | 189 | 17.06 | 1.43 | 1.34 | 9.9×10^-3^ | 2.0×10^-2^ |
| 1,5-Anhydroglucitol (1,5-AG) ***^i^** | 259 | 17.10 | 1.41 | 1.45 | 1.5×10^-4^ | 1.2×10^-3^ |
| Glucose **^#^** | 81 | 17.51 | 1.31 | 1.33 | 1.7×10^-2^ | 2.3×10^-2^ |
| Palmitic acid ***** | 187 | 19.10 | 1.14 | 1.35 | 3.7×10^-3^ | 1.5×10^-2^ |
| Allo-inositol ***** | 279 | 19.44 | 0.73 | 1.38 | 1.9×10^-3^ | 9.9×10^-3^ |
| Arachidonic acid **^#^** | 80 | 22.00 | 1.28 | 1.22 | 1.1×10^-2^ | 2.0×10^-2^ |
| Cholesterol ***** | 253 | 27.65 | 1.17 | 1.31 | 9.3×10^-3^ | 2.1×10^-3^ |
| ^a^ FC: fold change value for each metabolite was calculated by comparing UUO group vs Sham  group.  ^b^ VIP was obtained from OPLS-DA model with a threshold of 1.0.  ^c^ *P* value calculated by Mann-Whitney-Wilcoxon test.  ^d^ Adjusted *P* value calculated by false discovery rate method  ^*^Represents metabolites identified by the FIEHN library.  ^#^ Represents metabolites identified by the NIST library.  ^i^ Represents metabolites identified by the reference compounds. | | | | | | |

**Supplementary Table 2：**Twenty-three differential metabolites identified from healthy subjects *vs* CKD patients using GC-MS based untargeted metabolomics.

| **Primary ID** | **CKD vs Control** | | | | | |
| --- | --- | --- | --- | --- | --- | --- |
|  | **m/z** | **Rt(min)** | **^a^ FC** | **^b^ VIP** | **^c^ U test** | **^d^ FDR adjusted U test** |
| Lactic acid ***** | 158 | 7.16 | 0.30 | 1.25 | 4.12e-22 | 5.57e-22 |
| 2,3-Butanediol ***** | 176 | 7.17 | 0.35 | 1.19 | 2.79e-18 | 3.38e-18 |
| Valine ***** | 72 | 7.71 | 3.84 | 1.37 | 3.58e-29 | 8.24e-29 |
| 2-Hydroxybutyric acid ***** | 131 | 8.13 | 0.33 | 1.30 | 7.51e-29 | 1.44e-28 |
| Pentanoic acid **^#^** | 281 | 8.62 | 2.38 | 1.13 | 1.43e-26 | 2.34e-26 |
| 3-Hydroxybutyric acid **^#^** | 188 | 8.62 | 3.49 | 1.27 | 1.95e-26 | 2.99e-26 |
| Isoleucine ***** | 86 | 8.63 | 6.73 | 1.38 | 2.57e-31 | 6.57e-31 |
| 2-ketoisocaproic acid ***** | 110 | 9.32 | 0.26 | 1.45 | 4.47e-35 | 2.57e-34 |
| Hexanoic acid **^#^** | 200 | 9.33 | 0.25 | 1.49 | 3.51e-36 | 2.69e-35 |
| Urea ***^i^** | 189 | 10.18 | 8.68 | 1.02 | 5.68e-17 | 6.53e-17 |
| Allothreonine ***** | 57 | 10.45 | 3.04 | 1.10 | 3.16e-27 | 5.60e-27 |
| Threonine ***** | 155 | 10.47 | 8.22 | 1.34 | 1.34e-32 | 4.41e-32 |
| Pyroglutamic acid ***** | 158 | 13.39 | 9.51 | 1.18 | 9.06e-33 | 3.47e-32 |
| Pipecolic acid **^#^** | 156 | 13.40 | 11.60 | 1.05 | 2.81e-32 | 8.08e-32 |
| Threonic acid ***** | 102 | 13.56 | 12.00 | 1.63 | 1.29e-37 | 1.48e-36 |
| Ribonic acid **^#^** | 234 | 14.92 | 26.06 | 1.56 | 1.41e-38 | 3.27e-37 |
| Glucose **^#^** | 104 | 17.02 | 0.66 | 1.06 | 4.75e-13 | 4.97e-13 |
| 1,5-Anhydroglucitol  (1,5-AG) ***^i^** | 259 | 17.05 | 0.17 | 1.45 | 1.03e-33 | 4.74e-33 |
| Oleic Acid ***** | 122 | 20.00 | 0.23 | 1.23 | 4.17e-29 | 8.73e-29 |
| 9-Octadecenoic acid **^#^** | 174 | 20.01 | 0.30 | 1.11 | 2.39e-20 | 3.06e-20 |
| Stearic acid ***** | 341 | 20.82 | 0.53 | 1.19 | 2.57e-23 | 3.69e-23 |
| Tocopherol **^#^** | 267 | 24.95 | 0.66 | 1.04 | 1.66e-12 | 1.66e-12 |
| Cholesterol ***** | 165 | 27.53 | 0.58 | 1.20 | 2.48e-16 | 2.72e-16 |
| ^a^ FC: fold change value for each metabolite was calculated by comparing CKD group vs healthy control  group.  ^b^ VIP was obtained from OPLS-DA model with a threshold of 1.0.  ^c^ *P* value calculated by Mann-Whitney-Wilcoxon test.  ^d^ Adjusted *P* value calculated by false discovery rate method.  **^*^** Represents metabolites identified by the FIEHN library.  **^#^** Represents metabolites identified by the NIST library. | | | | | | |

^i^ Represents metabolites identified by the reference compounds.

**Supplementary Table 3:** Baseline characteristics of feces from 10 CKD patients and 10 age- and sex-matched healthy subjects.

The clinical samples were collected from the Renmin Hospital of Wuhan University. Data are presented as mean ± SD. BMI: body mass index; BUN: b1ood urea nitrogen.^***^P < 0.0001 for Creatinine, ^***^P < 0.0001 for BUN. A Mann-Whitney test test was used to determine significant difference between control subjects and CKD patients.

**Supplementary Table 4:** Baseline characteristics of feces from 15 CKD patients and 15 age- and sex-matched healthy subjects

The clinical samples were collected from Putuo People’s Hospital. Data are presented as mean ± SD. BMI: body mass index; BUN: b1ood urea nitrogen.^***^P < 0.0001 for Creatinine, ^***^P < 0.0001 for BUN. A Mann-Whitney test test was used to determine significant difference between control subjects and CKD patients.

**Supplementary Table 5:** Baseline characteristics of sera from 115 CKD patients and 113 age- and sex-matched healthy subjects.

The clinical samples were collected from the Affiliated Hospital of Nanjing University of Chinese Medicine. Data are presented as mean ± SD. BMI: body mass index; BUN: b1ood urea nitrogen.^***^P < 0.0001 for Creatinine, ^***^P < 0.0001 for BUN. A Mann-Whitney test test was used to determine significant difference between control subjects and CKD patients.

**Supplementary Table 6:** Baseline characteristics of sera from 110 CKD patients and 110 age- and sex-matched healthy subjects.

The clinical samples were collected from the Ningbo Hospital of Zhejiang University. Data are presented as mean ± SD. BMI: body mass index; BUN: b1ood urea nitrogen.^***^P < 0.0001 for Creatinine, ^***^P < 0.0001 for BUN. A Mann-Whitney test test was used to determine significant difference between control subjects and CKD patients.

**Supplementary Table 7:** **Baseline characteristics of sera from 100 CKD patients and 100 age- and sex-matched healthy subjects.**

| **Variables** | **Control (n=100)** | **CKD (n=100)** |
| --- | --- | --- |
| Age (years) | 55±19 | 60±16 |
| Sex (male/female) | 65/35 | 63/37 |
| BMI (Kg/m^2^) | 23.3±2.75 | 23.3±2.7 |
| Creatinine (μmol/L) | 81±13.2 | 486.8±169.3^***^ |
| BUN (mmol/L) | 5.2±1.3 | 26.7±13.0^***^ |
| Blood glucose (mmol/L) | 5.4±0.4 | 5.5±0.6 |
| eGFR (mL/min*1.73 cm^2^) | / | 20.8±14.8 |

The clinical samples were collected from the Putuo People’s Hospital. Data are presented as mean ± SD. BMI: body mass index; BUN: b1ood urea nitrogen.^***^P < 0.0001 for Creatinine, ^***^P < 0.0001 for BUN. A Mann-Whitney test test was used to determine significant difference between control subjects and CKD patients

**Supplementary** **Table 8. Primer pairs for real-time qPCR.**

| **Gene Sequence** |
| --- |

*Tgfb1* F: 5’- CCAGATCCTGTCCAAACTAAGG-3’

R: 5’- CTCTTTAGCATAGTAGTCCGCT-3’

*Smad2* F: 5’- CTCTCCAACGTTAACCGAAATG-3’

R: 5’- CACCTATGTAATACAAGCGCAC-3’

*Smad3*  F: 5’- ATTCCATTCCCGAGAACACTAA-3’

R: 5’- TAGGTCCAAGTTATTGTGTGCT-3’

*Rac1* F: 5’- CCACTGTCCCAATACTCCTATC-3’

R: 5’- CTTCTTCTCCTTCAGCTTCTCA-3’

*Keap1*  F: 5’-GACTGGGTCAAATACGACTGC-3’

R: 5’-GAATATCTGCACCAGGTAGTCC-3’

*Ncf2*  F: 5’-GAAGATACCTCTCCAGAATCCG-3’

R: 5’-TTCTTAGACACCATGTTCCGAA-3’

*Nsfl1c*  F: 5’- ATTCACCGAGATCTACGAGTTC-3’

R: 5’-TGAAGTATTCAGTGAGAGTGCC-3’

*Nos2* F: 5’- ATCTTGGAGCGAGTTGTGGATTGTC-3’

R: 5’-TCGTAATGTCCAGGAAGTAGGTGAGG-3’

*Il1b*  F: 5’-CACTACAGGCTCCGAGATGAACAAC-3’

R: 5’-TGTCGTTGCTTGGTTCTCCTTGTAC-3’

*Il6* F: 5’-CTCCCAACAGACCTGTCTATAC-3’

R: 5’-CCATTGCACAACTCTTTTCTCA-3’

*Tnf*  F: 5’-ATGTCTCAGCCTCTTCTCATTC-3’

R: 5’-GCTTGTCACTCGAATTTTGAGA-3’

*Tlr4*  F: 5’-GCCATCATTATGAGTGCCAATT-3’

R: 5’-AGGGATAAGAACGCTGAGAATT-3’

*Slc5a2* F: 5’- CTTGGCGGTGGCTGGATTTGAG-3’

R: 5’- GCGGAGGTACTGAGGCATTGTG-3’

| *B.fragilis*  F: 5’-TCRGGAAGAAAGCTTGCT-3’  R: 5’-CATCCTTTACCGGAATCCT-3’  All bacteria  F: 5’-TGSTGCAYGGYTGTCGTCA-3’  R: 5’-ACGTCRTCCMCACCTTCCTC-3’ |
| --- |

**II. Supplementary figures**

**
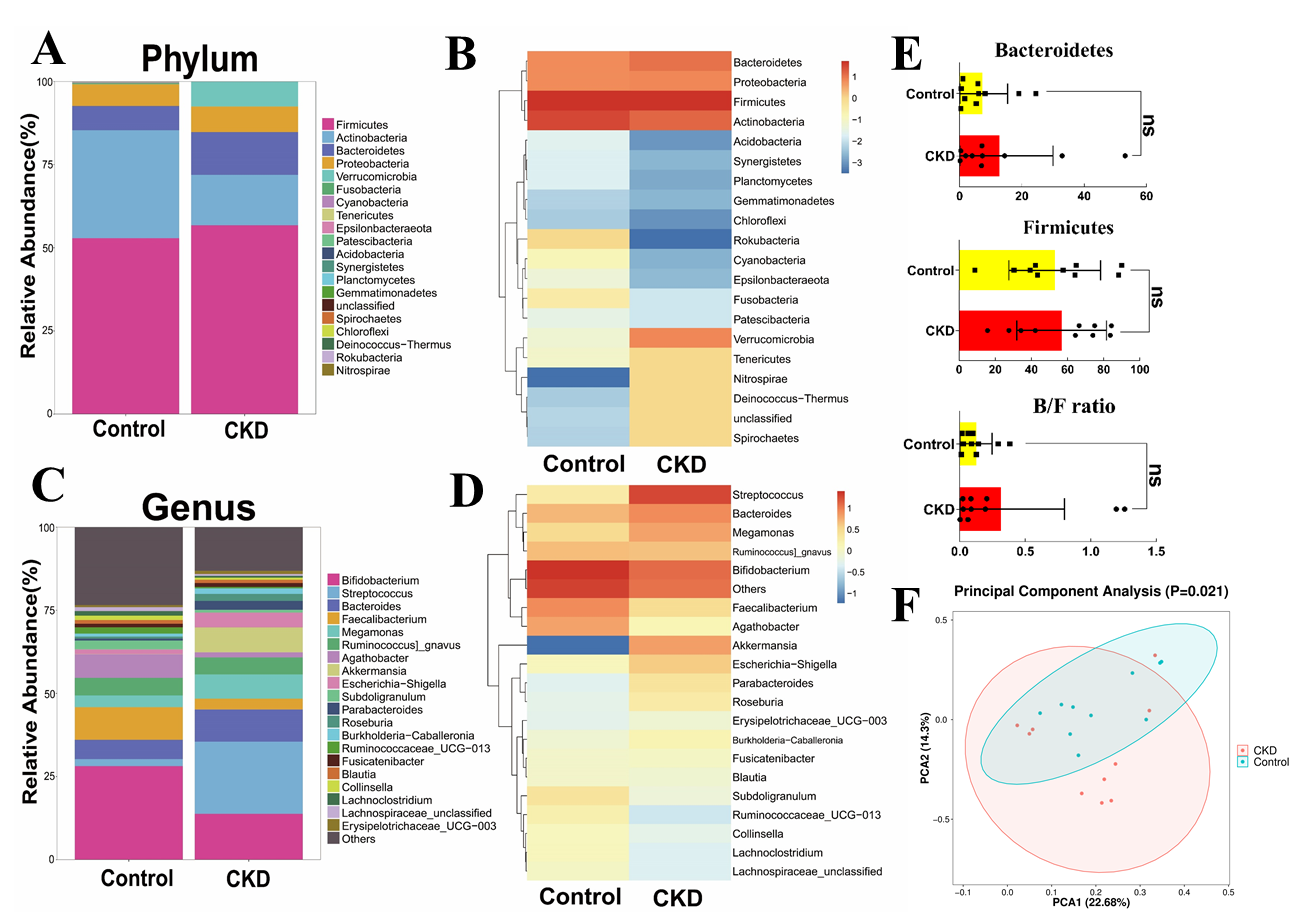
**

**Supplementary** **Fig. 1.** Microbiota composition of CKD and Control were analyzed by 16S rDNA bacteria gene sequencing (n = 10 for each group). (A) At the phylum level, relative abundance of bacteria (percentage of total bacteria) in CKD and Control. Different colors represent different bacteria at the same level. The bar from bottom to top corresponds to the relative abundance from high to low (only top 20 displayed). (B) At the phylum level, the heatmap shows the difference in bacteria between CKD and Control. The color gradient from blue to red is used to reflect the abundance from low to high. (C-D) At the genus level, the stacked bar charts and heatmap reflect the difference between CKD and Control (only top 20 displayed). (E) Relative abundance of phylum Bacteroidetes or Firmicutes in CKD and control, and Bacteroidetes to Firmicutes (B/F) ratio. *p* = 0.6305 for *Bacteroidetes*: Control vs. CKD; *p* = 0.7959 for *Firmicutes*: Control vs. CKD; *p* = 0.9118 for *B/F ratio*: Control vs. CKD. (F) PCA plot of CKD patients and controls. Data are presented as mean ± SD. Comparison in E and F were performed with a two-tailed Mann-Whitney U test. Adjusted *p*-values less than 0.05 were considered statistically significant for phylum and genus between CKD patients and healthy subjects. Individual data points are independent biological replicates unless otherwise stated.

**
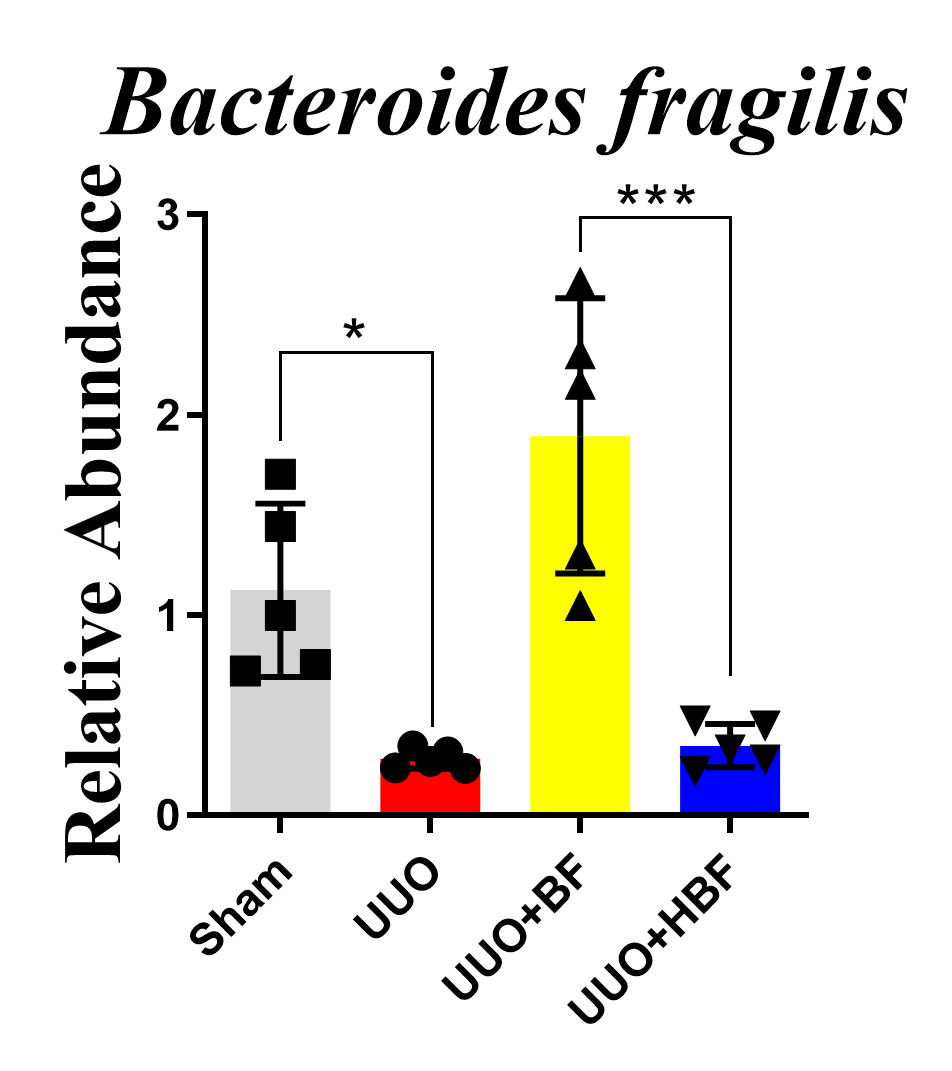
**

**Supplementary Fig. 2.** The relative abundance of *B. fragilis* in the sham, UUO, UUO + BF and UUO + HBF groups measured by qPCR (n=5). **P* = 0.0101 for *Bacteroides fragilis*: Sham vs. UUO, ****P* ＜0.0001 for *Bacteroides fragilis*: UUO+BF vs. UUO+HBF. Data are presented as mean ± SD. Comparisons in Fig. 2 were compared using One-Way ANOVA followed by Sidak’s multiple comparisons test. *P<0.05, **P<0.01, ***P<0.001. Individual data points are independent biological replicates unless otherwise stated.

**
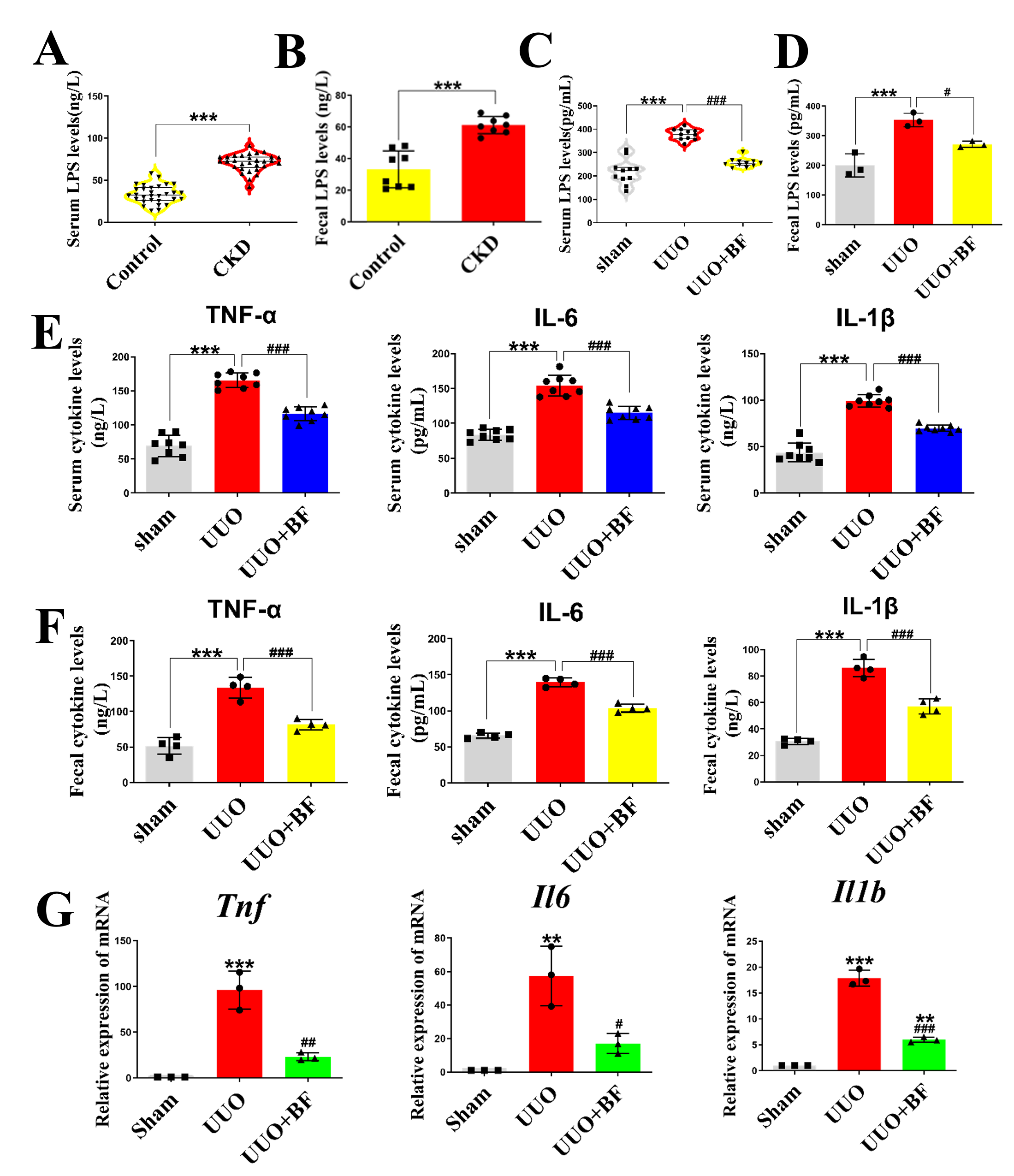
**

**Supplementary Fig. 3.** The anti-inflammatory effects of *B fragilis* UUO mice. (A) The LPS level in serum in the healthy control and CKD groups measured by ELISA (n=30), from the Affiliated Hospital of Nanjing University of Chinese Medicine (clinical information seen in Supplementary Table 6). ****P* ＜ 0.0001 for LPS: Control vs. CKD. (B) The LPS level in feces in the healthy control and CKD groups measured by ELISA (n=8), from the Department of Nephrology, Renmin Hospital of Wuhan University (clinical information seen in Supplementary Table 4). ****P* = 0.0002 for LPS: Control vs. CKD. (C) The LPS level in serum in the indicated groups measured by ELISA (n=11). ****P* ＜ 0.0001 for LPS: Sham vs. UUO, ^###^*P* ＜ 0.0001 for LPS: UUO vs. UUO+BF. (D) The LPS level in feces in the indicated groups measured by ELISA (n=3). ****P* = 0.0008 for LPS: Sham vs. UUO, ^#^*P* = 0.0187 for LPS: UUO vs. UUO+BF. (E) The levels of pro-inflammatory cytokine in serum measured by ELISA (IL, interleukin; TNF, tumor necrosis factor. n=8). ****P* ＜ 0.0001 for TNF-α: Sham vs. UUO, ^###^*P* ＜ 0.0001 for TNF-α: UUO vs. UUO+BF; ****P* ＜ 0.0001 for IL-6: Sham vs. UUO, ^###^*P* ＜ 0.0001 for IL-6: UUO vs. UUO+BF; ****P* ＜ 0.0001 for IL-1β: Sham vs. UUO, ^###^*P* ＜ 0.0001 for IL-1β: UUO vs. UUO+BF. (F) The levels of pro-inflammatory cytokine in feces measured by ELISA (n=4). ****P* ＜ 0.0001 for TNF-α: Sham vs. UUO, ^###^*P* = 0.0003 for TNF-α: UUO vs. UUO+BF; ****P* ＜ 0.0001 for IL-6: Sham vs. UUO, ^###^*P* ＜ 0.0001 for IL-6: UUO vs. UUO+BF; ****P* ＜ 0.0001 for IL-1β: Sham vs. UUO, ^###^*P* ＜ 0.0001 for IL-1β: UUO vs. UUO+BF. (G) qPCR showed the expression of inflammatory related mRNA in UUO model (n=3). ****P* = 0.0002 for *Tnf*: Sham vs. UUO, *P* = 0.1909 for *Tnf*: Sham vs. UUO+BF, ^##^*P* = 0.0010 for *Tnf*: UUO vs. UUO+BF; ***P* = 0.0021 for *Il6*: Sham vs. UUO, *P* = 0.3189 for *Il6*: Sham vs. UUO+BF, ^#^*P* = 0.0116 for *Il6*: UUO vs. UUO+BF; ****P* ＜ 0.0001 for *Il-1b*: Sham vs. UUO, ***P* = 0.0019 for *Il-1b*: Sham vs. UUO+BF, ^###^*P* ＜ 0.0001 for *Il-1b*: UUO vs. UUO+BF. GAPDH was used as an internal control. Data are presented as mean ± SD. Comparison in A, B were performed with a two-tailed Ttest. Comparisons in C-G were compared using One-Way ANOVA followed by Sidak’s multiple comparisons test. ^*^P<0.05, ^**^P<0.01, ^***^P<0.001 (compared with sham or healthy control group), ^#^P<0.05, ^##^P<0.01, ^###^P<0.001 (compared with UUO group). Individual data points are independent biological replicates unless otherwise stated.

**
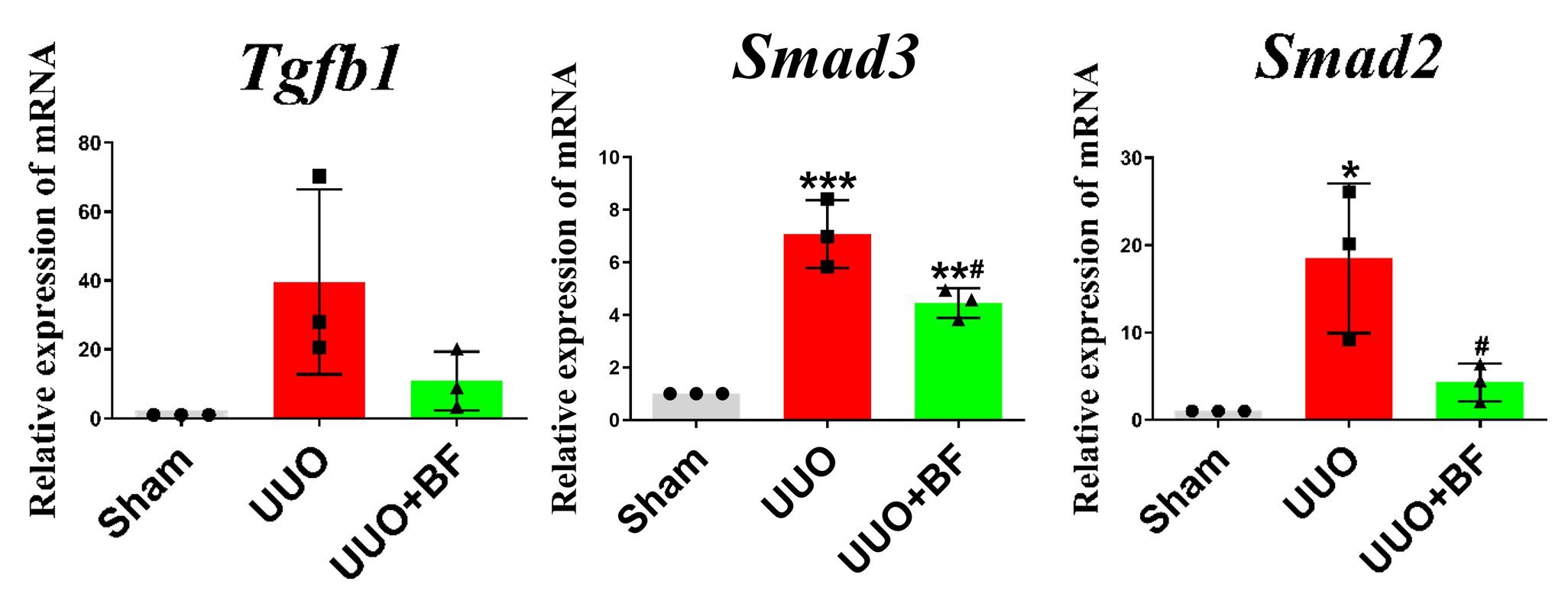
**

**Supplementary Fig. 4.** qPCR analysis of TGF-β/Smad pathways in UUO model. qPCR showed the expression of TGF-β/Smad signaling pathway related mRNA in UUO model (n=3). *P* = 0.0787 for *Tgfb1* : Sham vs. UUO, *P* = 0.8654 for *Tgfb1* : Sham vs. UUO+BF, *P* = 0.2032 for *Tgfb1* : UUO vs. UUO+BF; ****P* = 0.0003 for *Smad3* : Sham vs. UUO, ***P* = 0.0060 for *Smad3* : Sham vs. UUO+BF; ^#^*P* = 0.0223 for *Smad3* : UUO vs. UUO+BF; **P* = 0.0171 for *Smad2* : Sham vs. UUO, *P* = 0.8418 for *Smad2*: Sham vs. UUO+BF, ^#^*P* = 0.0428 for *Smad2* : UUO vs. UUO+BF. GAPDH was used as an internal control. Data are presented as mean ± SD. Comparisons in Fig. 4 were compared using One-Way ANOVA followed by Sidak’s multiple comparisons test. ^*^P<0.05, ^**^P<0.01, ^***^P<0.001 (compared with sham group). ^#^P<0.05, ^##^P<0.01, ^###^P<0.001(compared with UUO group). Individual data points are independent biological replicates unless otherwise stated.

**
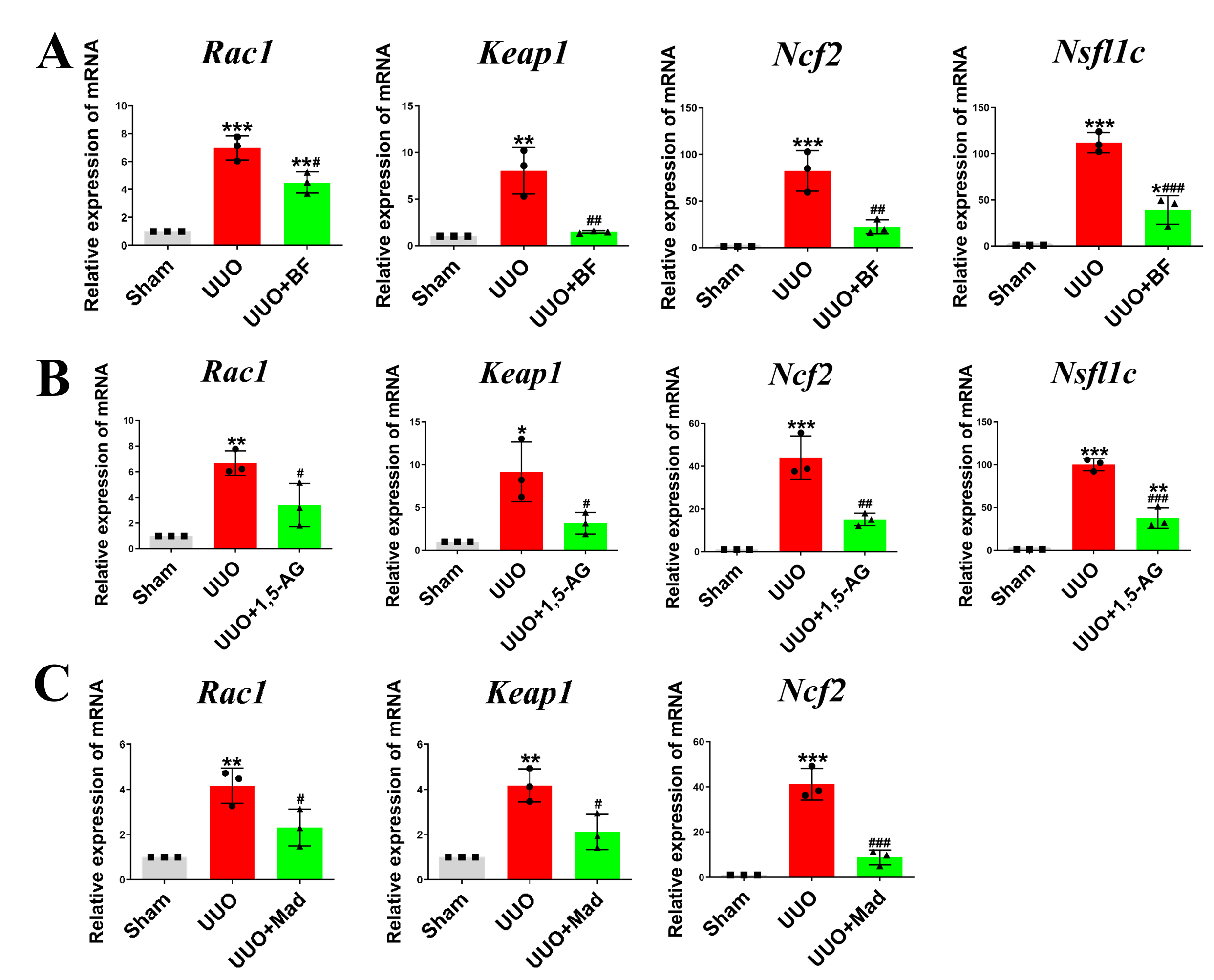
**

**Supplementary Fig. 5.** qPCR analysis of oxidative stress pathways in UUO model. (A-C) qPCR showed the expression of oxidative stress related mRNA in UUO model (n=3). (A) ****p* = 0.0001 for *Rac1*: Sham vs. UUO, ***p* = 0.002 for *Rac1*: Sham vs. UUO+BF, ^#^*p* = 0.0115 for *Rac1*: UUO vs. UUO+BF; ***p* = 0.0029 for *Keap1*: Sham vs. UUO, *p* = 0.9751 for *Keap1*: Sham vs. UUO+BF, ^##^*p* = 0.0042 for *Keap1*: UUO vs. UUO+BF; ****p* = 0.0009 for *Ncf2*: Sham vs. UUO, *p* = 0.2642 for *Ncf2*: Sham vs. UUO+BF, ^##^*p* = 0.0043 for *Ncf2*: UUO vs. UUO+BF; ****p* ＜ 0.0001 for *Nsfl1c*: Sham vs. UUO, **p* = 0.0158 for *Nsfl1c*: Sham vs. UUO+BF, ^###^*p* = 0.0005 for *Nsfl1c*: UUO vs. UUO+BF. (B) ***p* = 0.0024 for *Rac1*: Sham vs. UUO, *p* = 0.1112 for *Rac1*: Sham vs. UUO+1,5-AG, ^#^*p* = 0.0338 for *Rac1*: UUO vs. UUO+1,5-AG; **p* = 0.0103 for *Keap1*: Sham vs. UUO, *p* = 0.5982 for *Keap1*: Sham vs. UUO+1,5-AG, ^#^*p* = 0.0415 for *Keap1*: UUO vs. UUO+1,5-AG; ****p* = 0.0004 for *Ncf2*: Sham vs. UUO, *p* = 0.0847 for *Ncf2*: Sham vs. UUO+1,5-AG, ^##^*p* = 0.0033 for *Ncf2*: UUO vs. UUO+1,5-AG; ****p* ＜ 0.0001 for *Nsfl1c*: Sham vs. UUO, ***p* = 0.0041 for *Nsfl1c*: Sham vs. UUO+1,5-AG, ^###^*p* = 0.0002 for *Nsfl1c*: UUO vs. UUO+1,5-AG. (C) ***p* = 0.0031 for *Rac1*: Sham vs. UUO, *p* = 0.1413 for *Rac1*: Sham vs. UUO+Mad, ^#^*p* = 0.0391 for *Rac1*: UUO vs. UUO+ Mad; ***p* = 0.0022 for *Keap1*: Sham vs. UUO, *p* = 0.1956 for *Keap1*: Sham vs. UUO+ Mad, ^#^*p* = 0.0186 for *Keap1*: UUO vs. UUO+ Mad; ****p* ＜ 0.0001 for *Ncf2*: Sham vs. UUO, *p* = 0.2098 for *Ncf2*: Sham vs. UUO+ Mad, ^###^*p* = 0.0003 for *Ncf2*: UUO vs. UUO+ Mad. GAPDH was used as an internal control. Data are presented as mean ± SD. Comparisons in Fig. 5 were compared using One-Way ANOVA followed by Sidak’s multiple comparisons test. ^*^P<0.05, ^**^P<0.01, ^***^P<0.001 (compared with sham group). ^#^P<0.05, ^##^P<0.01, ^###^P<0.001(compared with UUO group). Individual data points are independent biological replicates unless otherwise stated.


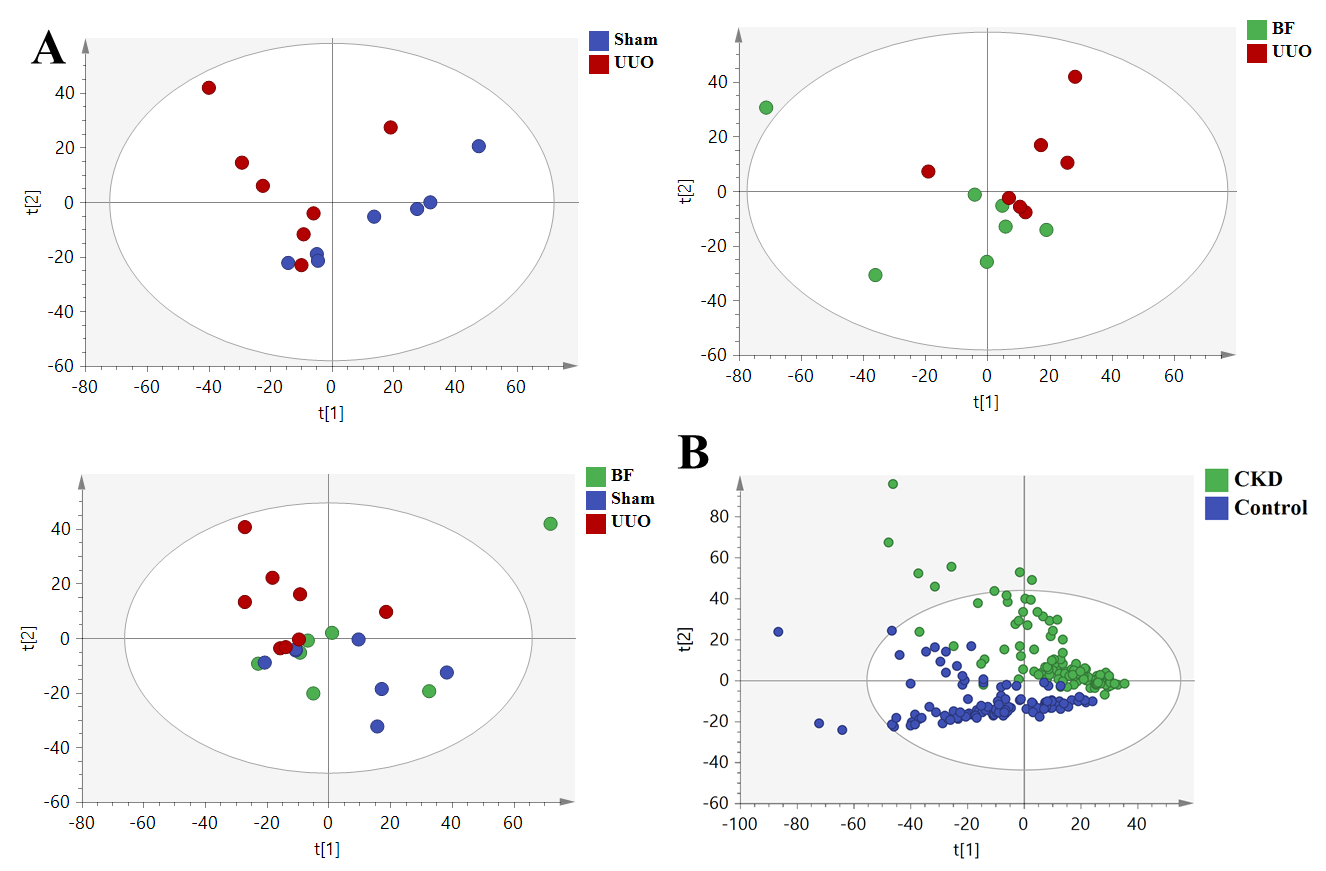


**Supplementary Fig. 6.** PCA plots in the indicated groups. (A) An unsupervised PCA was performed in the Sham, UUO and BF groups (n=7). (B) An unsupervised PCA was performed in the CKD and healthy control groups (CKD: n=115, Control: n=113). PCA principal component analysis. Individual data points are independent biological replicates unless otherwise stated.

**
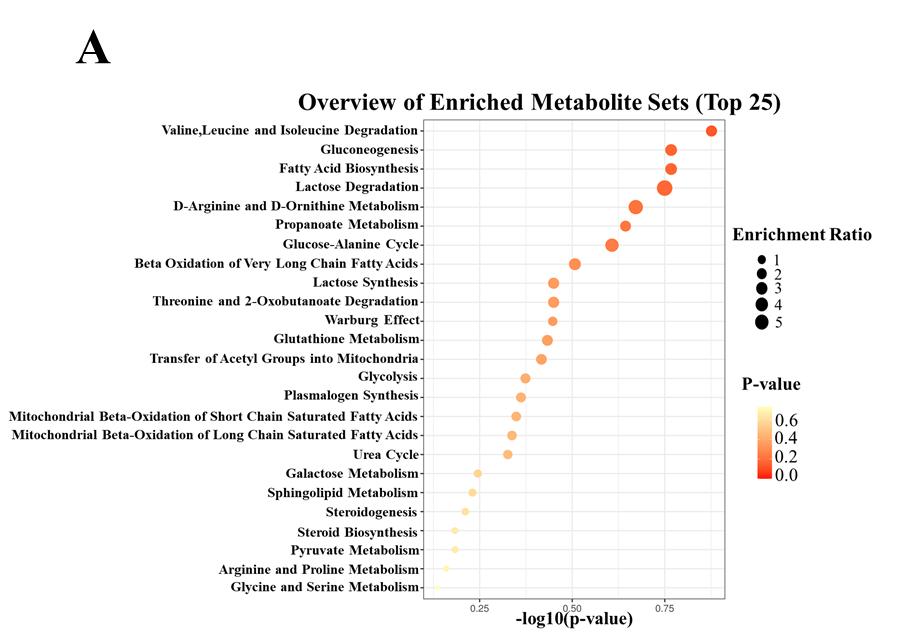
**

**
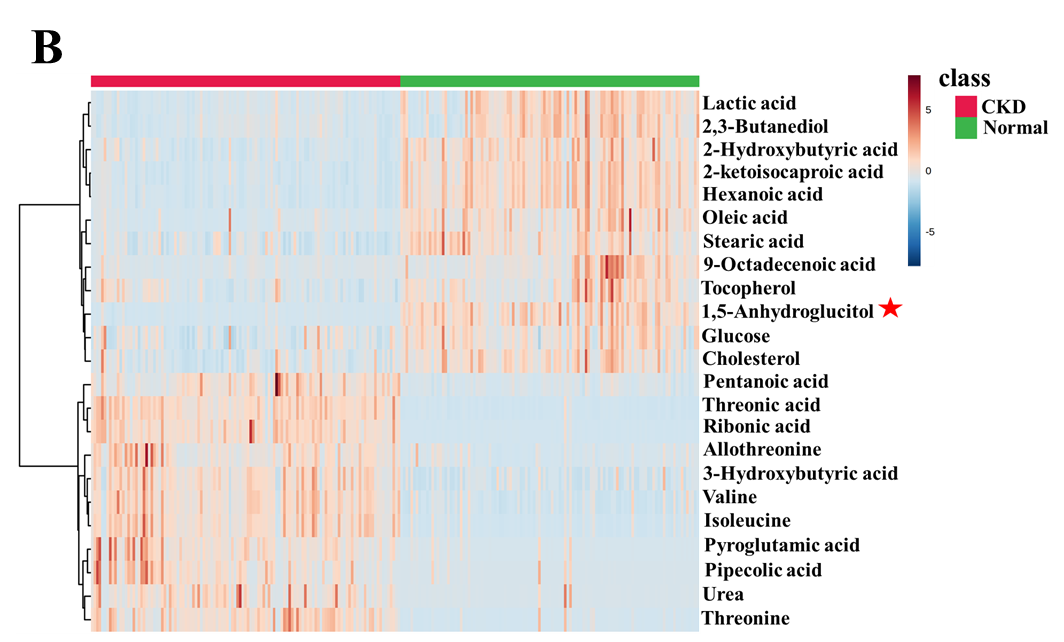
**

**Supplementary Fig. 7.** GC-MS based metabolomics analysis of serum samples from healthy control and CKD groups. (A) Disturbed metabolic pathways in the CKD and healthy subjects. (B) Heatmap of the differential metabolites (CKD: n=115, Control: n=113).

**
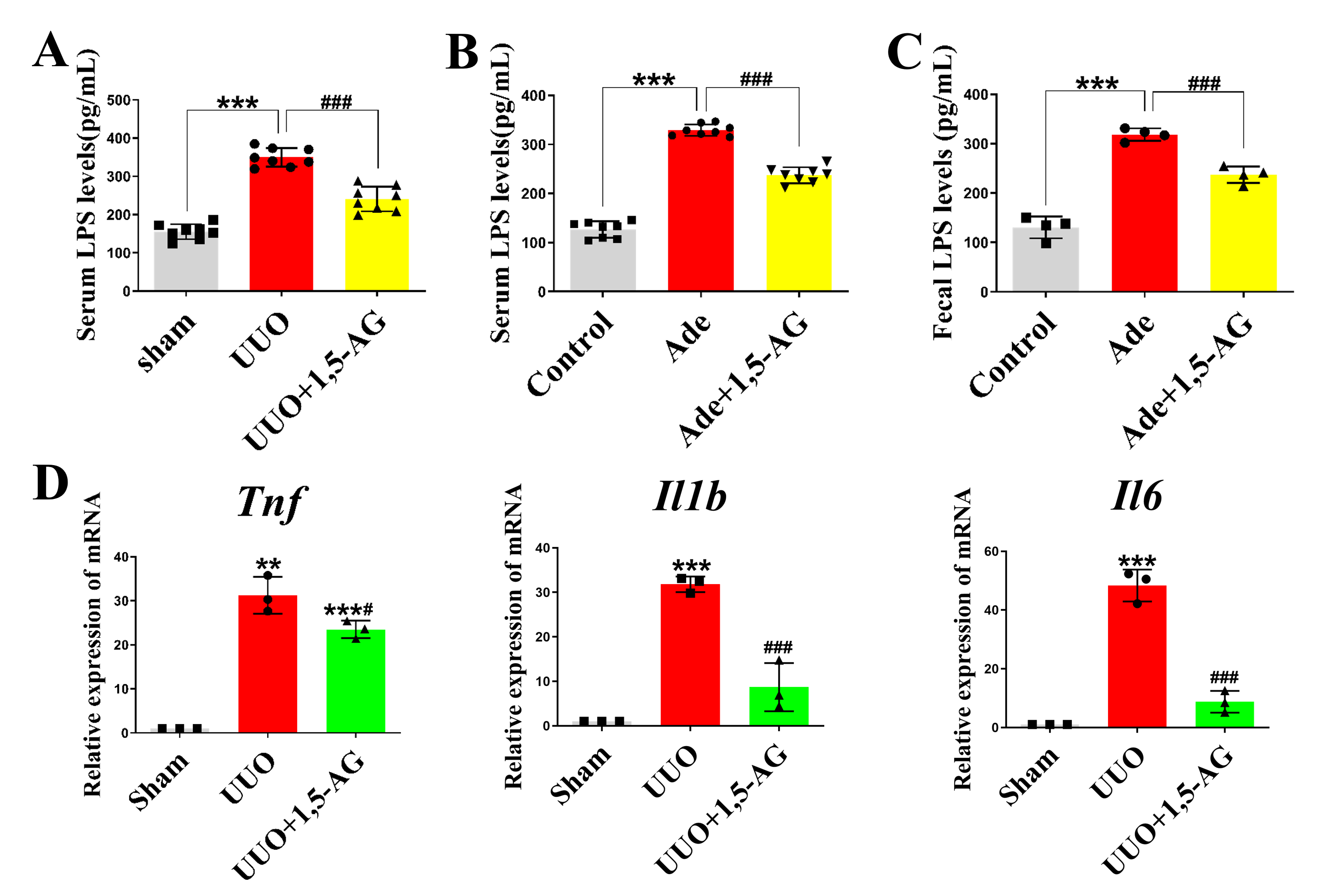
**

**Supplementary Fig. 8.** The anti-inflammatory effects of 1,5-AG in UUO and adenine models. (A) 1,5-AG decreased serum LPS level in UUO group (n=8). ****p* ＜ 0.0001 for LPS: Sham vs. UUO, ^###^*P* ＜ 0.0001 for LPS: UUO VS. UUO+1,5-AG. (B) 1, 5-AG decreased serum LPS level in adenine group (n=8). ****p* ＜ 0.0001 for LPS: Control vs. Ade, ^###^*P* ＜ 0.0001 for LPS: Ade VS. Ade+1,5-AG. (C) 1,5-AG decreased fecal LPS level in adenine group (n=4). ****p* ＜ 0.0001 for LPS: Control vs. Ade, ^###^*P* = 0.0002 for LPS: Ade VS. Ade+1,5-AG. (D) qPCR showed the expression of inflammatory related mRNA in UUO model (n=3). ***p*＜0.0001 for *Tnf*: Sham vs. UUO, ****p*=0.0001 for *Tnf*: Sham vs. UUO+1,5-AG, ^#^*p*=0.0350 for *Tnf*: UUO vs. UUO+1,5-AG; ****p*＜0.0001 for *Il1b*: Sham vs. UUO, *p*=0.0825 for *Il1b*: Sham vs. UUO+1,5-AG, ^###^*p*=0.0004 for *Il1b*: UUO vs. UUO+1,5-AG; ****p*＜0.0001 for *Il6*: Sham vs. UUO, *p*=0.1297 for *Il6*: Sham vs. UUO+1,5-AG, ^###^*p*＜0.0001 for *Il6*: UUO vs. UUO+1,5-AG. Data are presented as mean ± SD. Comparisons in Fig. 8 were compared using One-Way ANOVA followed by Sidak’s multiple comparisons test. ^*^P<0.05, ^**^P<0.01, ^***^P<0.001 (compared with sham or control group). ^#^P<0.05, ^##^P<0.01, ^###^P<0.001(compared with UUO or adenine group). Individual data points are independent biological replicates unless otherwise stated.


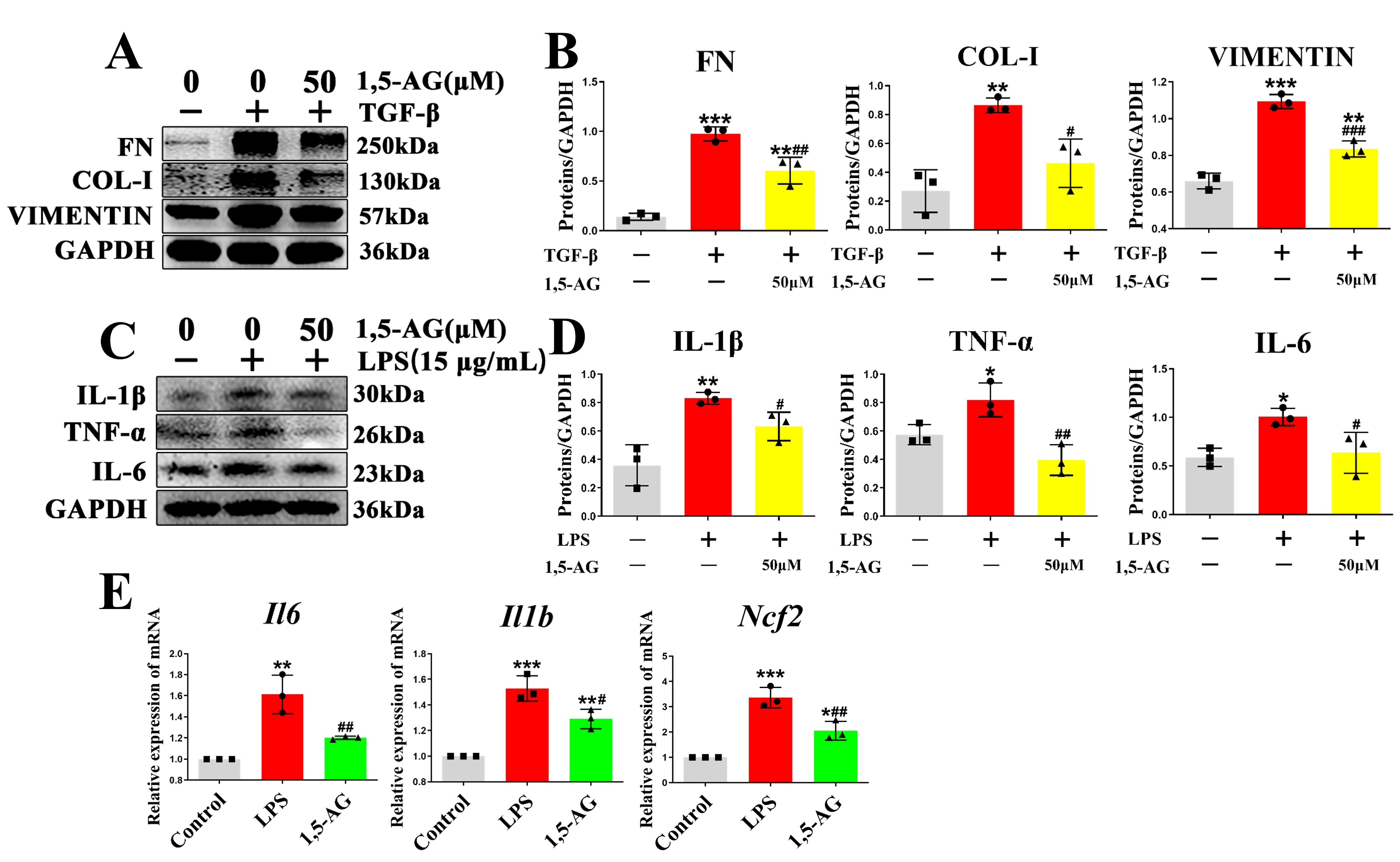


**Supplementary Fig. 9.** The anti-fibrotic and anti-inflammatory effects of 1,5-AG *in vitro*. (A) Fibrosis protein expressions in HK-2 cells treated with TGF-β (10 ng/mL) in presence or absence of 1,5-AG for 48h as assayed by Western blotting (n=3). (B) Quantification analysis of Supplementary Fig. 8A. ****P* ＜0.0001 for FN: Control vs. model, ***p* =0.0022 for FN: Control vs. 1,5-AG, ^##^*p* =0.0070 for FN: model vs. 1,5-AG; ***P* =0.0045 for COL-Ⅰ: Control vs. model, *p* =0.3289 for COL-Ⅰ: Control vs. 1,5-AG, ^#^*p* =0.0293 for COL-Ⅰ: model vs. 1,5-AG; ****P* ＜0.0001 for VIMENTIN: Control vs. model, ***p* =0.0062 for VIMENTIN: Control vs. 1,5-AG, ^##^*p* = 0.0008 for VIMENTIN: model vs. 1,5-AG. (C) Inflammatory factor expressions in HMC cells treated with LPS (15 μg/mL) in presence or absence of 1,5-AG for 48h as assayed by Western blotting. (D) Quantification analysis of Supplementary Fig. 8C. ***p* = 0.0056 for IL-1β：Control vs. model, p = 0.0549 for IL-1β: Control vs. 1,5-AG, ^#^p = 0.0340 for IL-1β: model vs. 1,5-AG, Comparison in IL-1β were performed with a two-tailed T test; **p* = 0.0494 for TNF-α: Control vs. model, ^##^*p* = 0.0043 for TNF-α: model vs. 1,5-AG; **p* = 0.0236 for IL-6: Control vs. model, ^#^*p* = 0.0387 for TNF-α: model vs. 1,5-AG. (E) qPCR showed the expression of inflammatory related mRNA in HMC cells (n=3).***P* = 0.0012 for *Il6*: Control vs. model, *P* = 0.1635 for *Il6*: Control vs. 1,5-AG, ^##^*P* = 0.0093 for *Il6*: model vs. 1,5-AG; ****P* = 0.0003 for *Il1b*: Control vs. model, ***P* = 0.0080 for *Il1b*: Control vs. 1,5-AG, ^#^*P* = 0.0209 for *Il1b*: model vs. 1,5-AG; ****P* = 0.0003 for *Ncf2*: Control vs. model, **P* = 0.0197 for *Ncf2*: Control vs. 1,5-AG, ^##^*P* = 0.0069 for *Ncf2*: model vs. 1,5-AG. Data are presented as mean ± SD. Comparisons in B, D and E were compared using One-Way ANOVA followed by Sidak’s multiple comparisons test. ^*^P<0.05, ^**^P<0.01, ^***^P<0.001 (compared with control group). ^#^P<0.05, ^##^P<0.01, ^###^P<0.001(compared with model group). Individual data points are independent biological replicates unless otherwise stated.


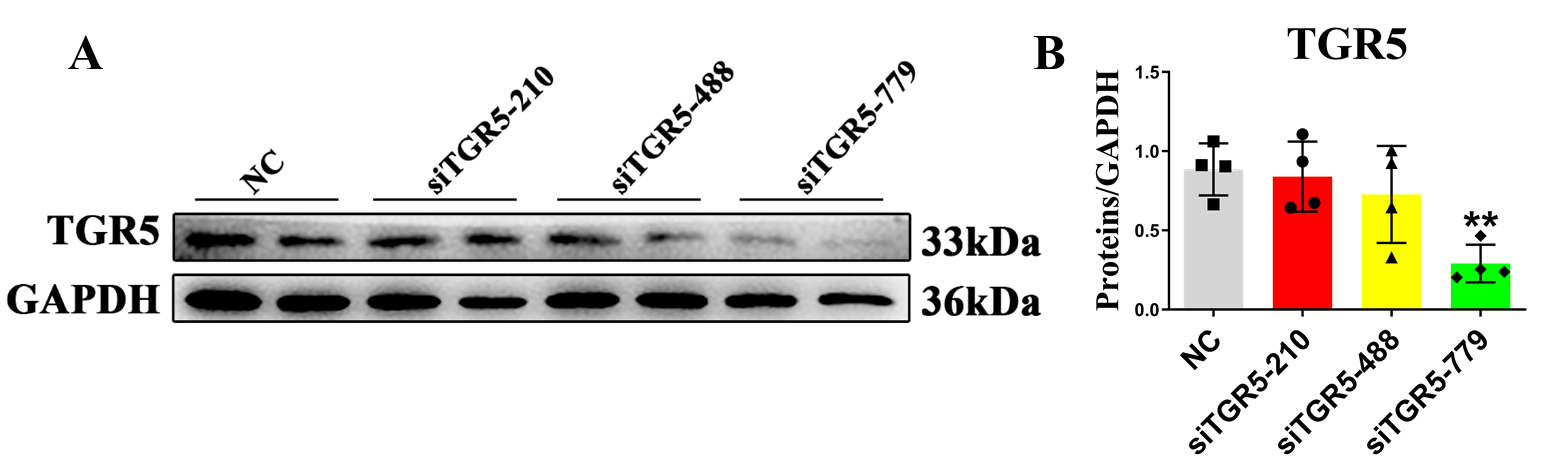


**Supplementary Fig. 10.** Effect of different siRNAs on expression of TGR5 in primary mouse renal tubular epithelial cells. (A) TGR5 expression assayed by Western blot (n=4). (B) Quantification analyses of Supplementary Fig. 10A. *p* = 0.9875 for TGR5: NC vs. siTGR5-210, *p* = 0.6794 for TGR5: NC vs. siTGR5-448, ***p* = 0.0060 for TGR5: NC vs. siTGR5-779. Data are presented as mean ± SD. Comparisons in B were compared using One-Way ANOVA followed by Sidak’s multiple comparisons test. ^*^P<0.05, ^**^P<0.01, ^***^P<0.001 (compared with NC group). NC represents the negative control. Individual data points are independent biological replicates unless otherwise stated.

**
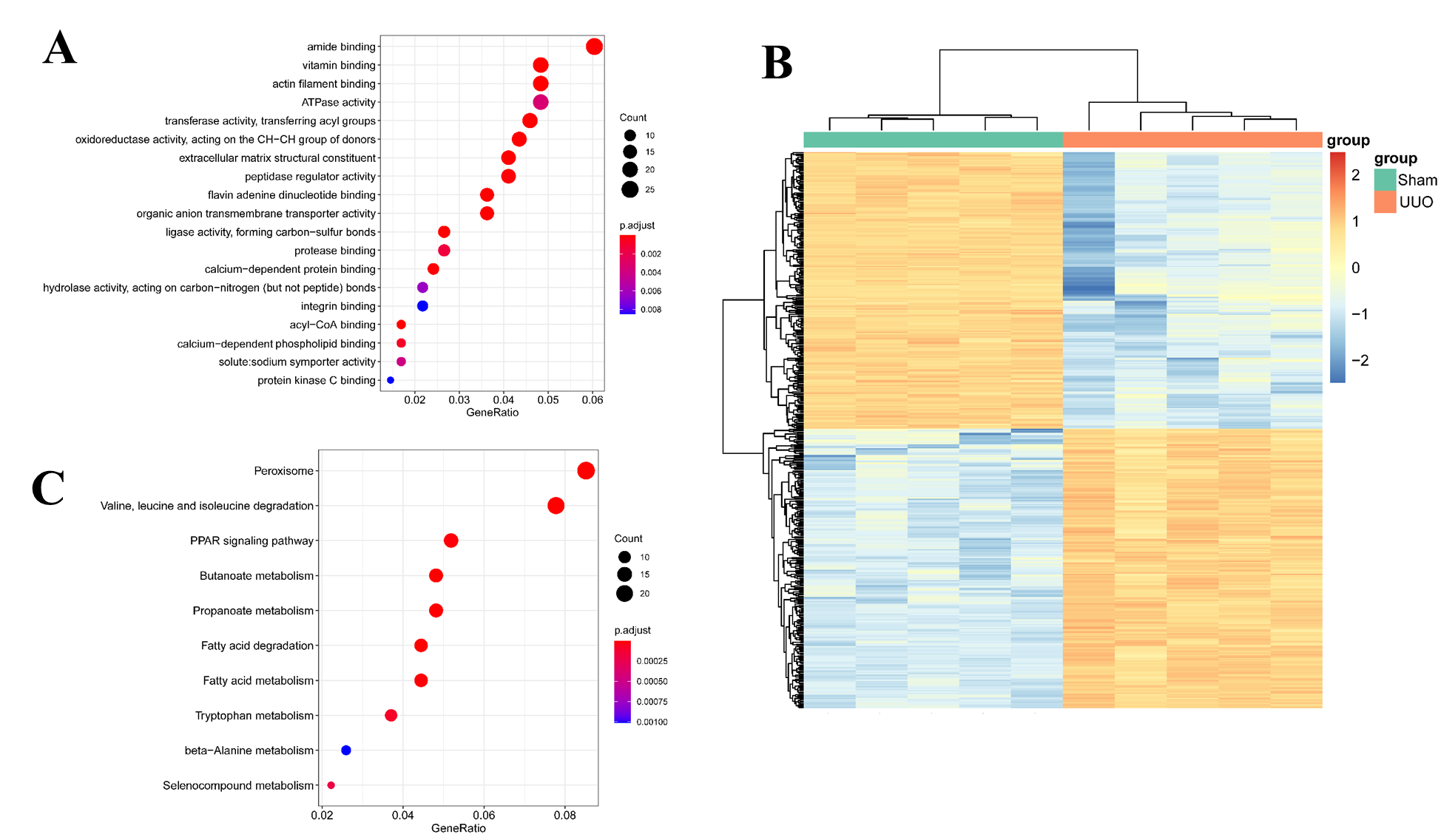
**

**Supplementary Fig. 11.** The proteomics analyses of kidney tissues in UUO model. (A) GO analyses in the Sham and UUO groups. (B) Heatmap of the differential proteins. (C) KEGG pathway analyses of the differential protein.


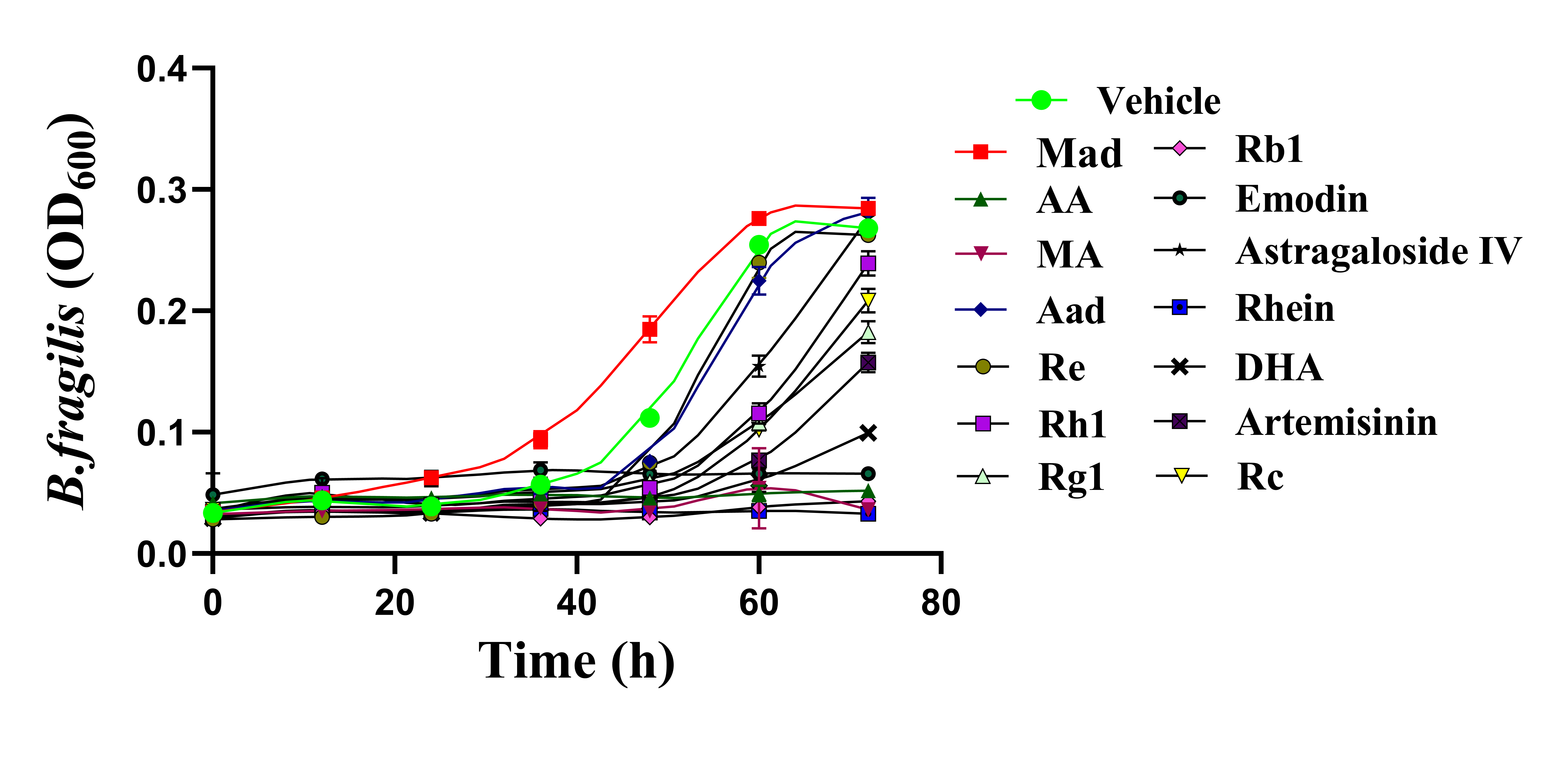


**Supplementary Fig. 12.** Growth-modulating effect of 14 active components associated with CKD on *B. fragilis* *in vitro* (n=4)*.* 14 active components include madecassoside (Mad), asiatic acid (AA), asiaticoside (Aad), madecassic acid (MA), ginsenoside Re, Rc, Rg1, Rh1, Rb1, artemisinin, emodin, astragaloside IV, dihydroartemisinin (DHA), and rhein. Data are presented as mean ± SD.

**
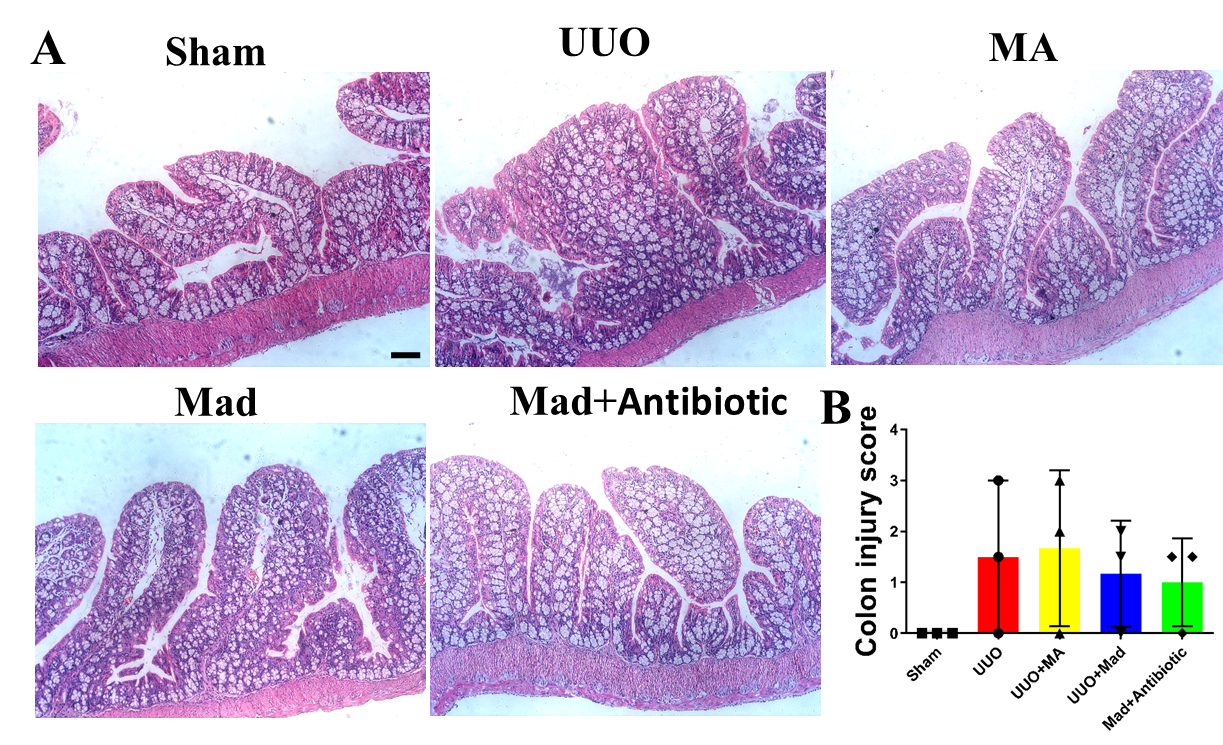
**

**Supplementary Fig. 13.** Representative photomicrographs of the H&E staining from colon tissue of all groups in UUO model. (A) Representative photomicrographs of the H&E staining from colon tissue of Sham, UUO, UUO + MA, UUO + Mad, UUO + Mad + Antibiotic mice (H&E staining; scale bar, 100 μm; magnification, ×100). (B) Bar graph depicts colon injury scores based on H&E staining (n=3). *P* = 0.6404 for Colon injury score: Sham vs. UUO, *P* = 0.5281 for Colon injury score: Sham vs. UUO+MA, *P* = 0.8478 for Colon injury score: Sham vs. UUO+Mad, *P* = 0.9217 for Colon injury score: Sham vs. Mad+Antibiotic, *P* ＞ 0.9999 for Colon injury score: UUO vs. UUO+MA, *P* = 0.9999 for Colon injury score: UUO vs. UUO+Mad, *P* = 0.9984 for Colon injury score: UUO vs. Mad+Antibiotic. Data are presented as mean ± SD. Individual data points are independent biological replicates unless otherwise stated.

**
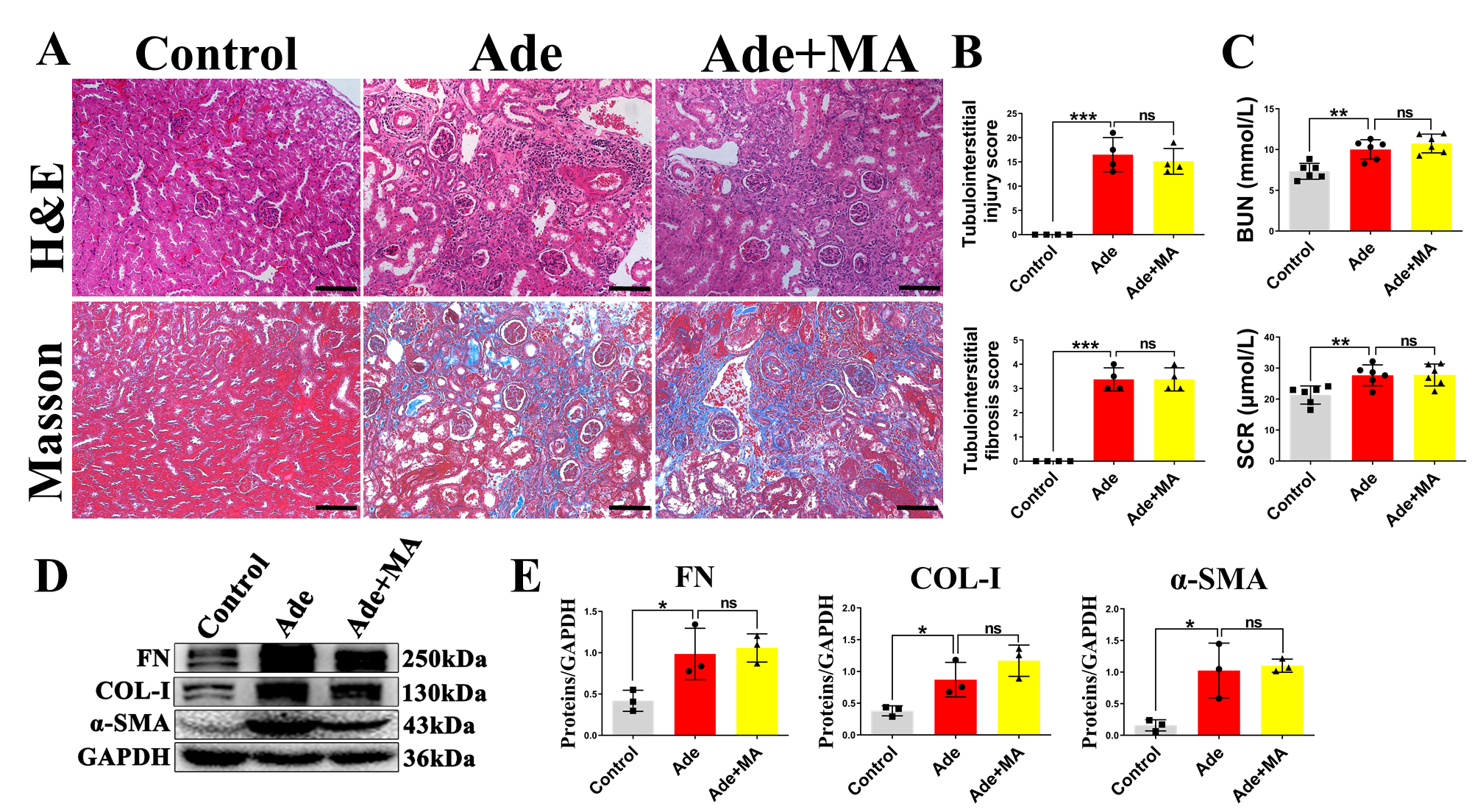
**

**Supplementary Fig. 14.** The effect of madecassic acid (MA) on renal fibrosis using adenine model. (A) Representative photomicrographs of the H&E staining and Masson’s trichrome staining from left kidneys of Control, Ade, Ade + MA mice (H&E and Masson’s staining; scale bar, 100 μm; magnification, ×200). (B) Bar graph depicts renal injury scores and renal interstitial fibrosis scores based on H&E staining or Masson’s trichrome staining (n=4). ****p* ＜ 0.0001 for Tubulointerstitial injury score: Control vs. Ade, *p* = 0.7147 for Tubulointerstitial injury score: Ade vs. Ade+MA; ****p* ＜ 0.0001 for Tubulointerstitial fibrosis score: Control vs. Ade, *p* ＞ 0.9999 for Tubulointerstitial fibrosis score: Ade vs. Ade+MA. (C) Biochemical parameters including BUN and Scr in each of mice (n=6). ***p* = 0.0017 for BUN: Control vs. Ade, p=0.4680 for BUN: Ade vs. Ade+MA; ***p* = 0.0087 for SCR: Control vs. Ade, *p* = 0.9978 for SCR: Ade vs. Ade+MA. (D) Kidney expression of FN, Col Ⅰ, and α-SMA from all groups, assayed by Western blot. (E) Quantification analyses of Supplementary Fig. 14D (n=3).**p* = 0.0372 for FN: Control vs. Ade, *p*=0.9086 for FN: Ade vs. Ade+MA; **p* = 0.0396 for COL-Ⅰ: Control vs. Ade, *p*=0.2314 for COL-Ⅰ: Ade vs. Ade+MA, Comparison in COL-1 were performed with a two-tailed T test; **p* = 0.0132 for α-SMA: Control vs. Ade, *p*=0.9301 for α-SMA: Ade vs. Ade+MA. Data are presented as mean ± SD. Comparisons in B, C and E were compared using One-Way ANOVA followed by Sidak’s multiple comparisons test. ^*^P<0.05, ^**^P<0.01, ^***^P<0.001 (compared with control group), ^#^P<0.05, ^##^P<0.01, ^###^P<0.001 (compared with adenine group). Individual data points are independent biological replicates unless otherwise stated.

**
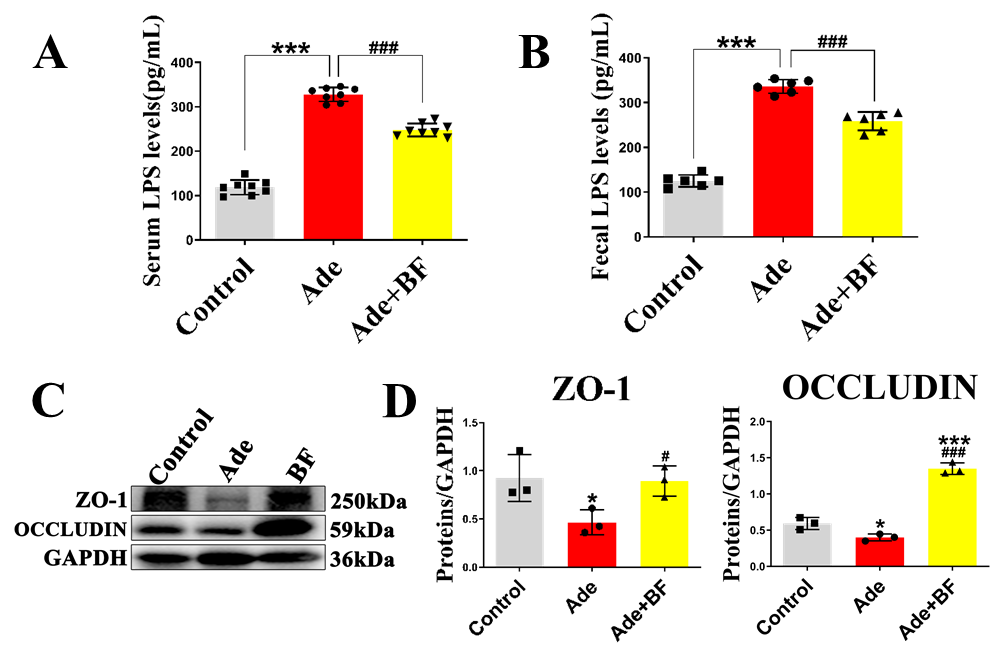
**

**Supplementary Fig. 15.** The anti-inflammatory effects of 1,5-AG using adenine model. (A) The LPS level in serum in the control, Ade, Ade + BF groups measured by ELISA (n=8). ****p* ＜ 0.0001 for LPS: Control vs. Ade, ^###^*p* ＜ 0.0001 for LPS: Ade vs. Ade+BF. (B) The LPS level in feces in the control, Ade, Ade + BF groups measured by ELISA (n=6). ****p* ＜ 0.0001 for LPS: Control vs. Ade, ^###^*p* ＜ 0.0001 for LPS: Ade vs. Ade+BF. (C) Kidney expression of ZO-1 and Occludin from all groups, assayed by Western blot (n=3). (D) Quantification analyses of Supplementary Fig. 15C. **P* = 0.0442 for ZO-1: Control vs. Ade, ^#^*p* = 0.0222 for ZO-1: Ade vs. Ade+BF; **p* = 0.0253 for OCCLUDIN: Control vs. Ade, ****p* = 0.0003 for OCCLUDIN: Control vs. Ade+BF, ^###^*p* ＜ 0.0001 for OCLUDIN: Ade vs. Ade+BF. Comparison in OCCLUDIN were performed with a two-tailed T test . Data are presented as mean ± SD. Comparison in D were performed with a two-tailed T test. Comparisons in A and B were compared using One-Way ANOVA followed by Sidak’s multiple comparisons test. ^*^P<0.05, ^**^P<0.01, ^***^P<0.001 (compared with control group). ^#^P<0.05, ^##^P<0.01, ^###^P<0.001(compared with adenine group). Individual data points are independent biological replicates unless otherwise stated.


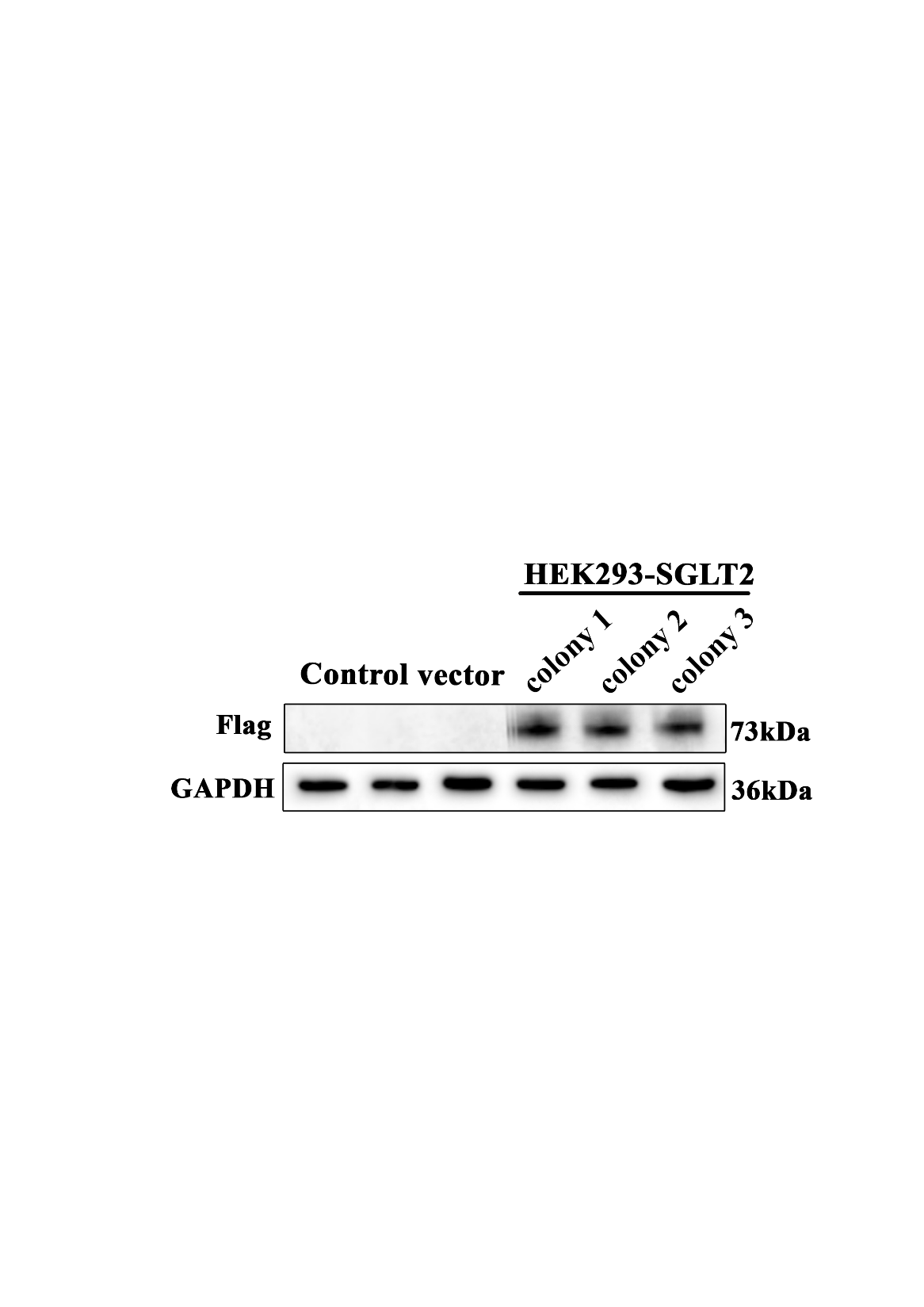


**Supplementary Fig. 16.** Expression of SGLT2 in three HEK-293 cells stably transfected SCL5A2 colonies. Individual data bands are independent biological replicates.


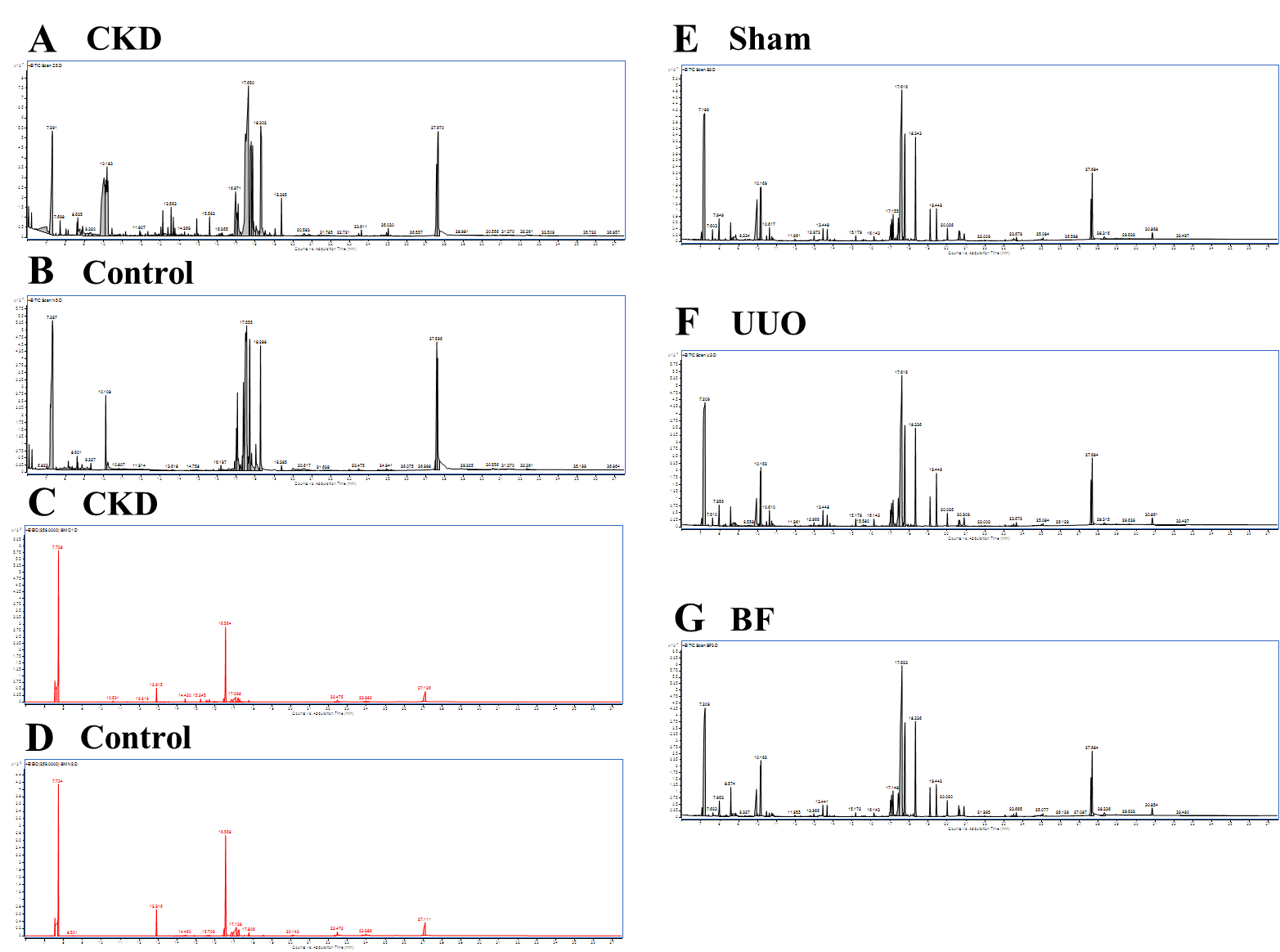


**Supplementary Fig. 17.** Representative chromatograms of GC-MS. (A-B) Representative total ion chromatogram (TIC) of human serum by GC-MS based untargeted metabolomics. (C-D) Representative chromatogram of human serum by GC-MS based targeted metabolomics using SIM mode at m/z 259. (E-G) Representative TIC of serum in mice among sham, UUO and BF groups by GC-MS based untargeted metabolomics.

**
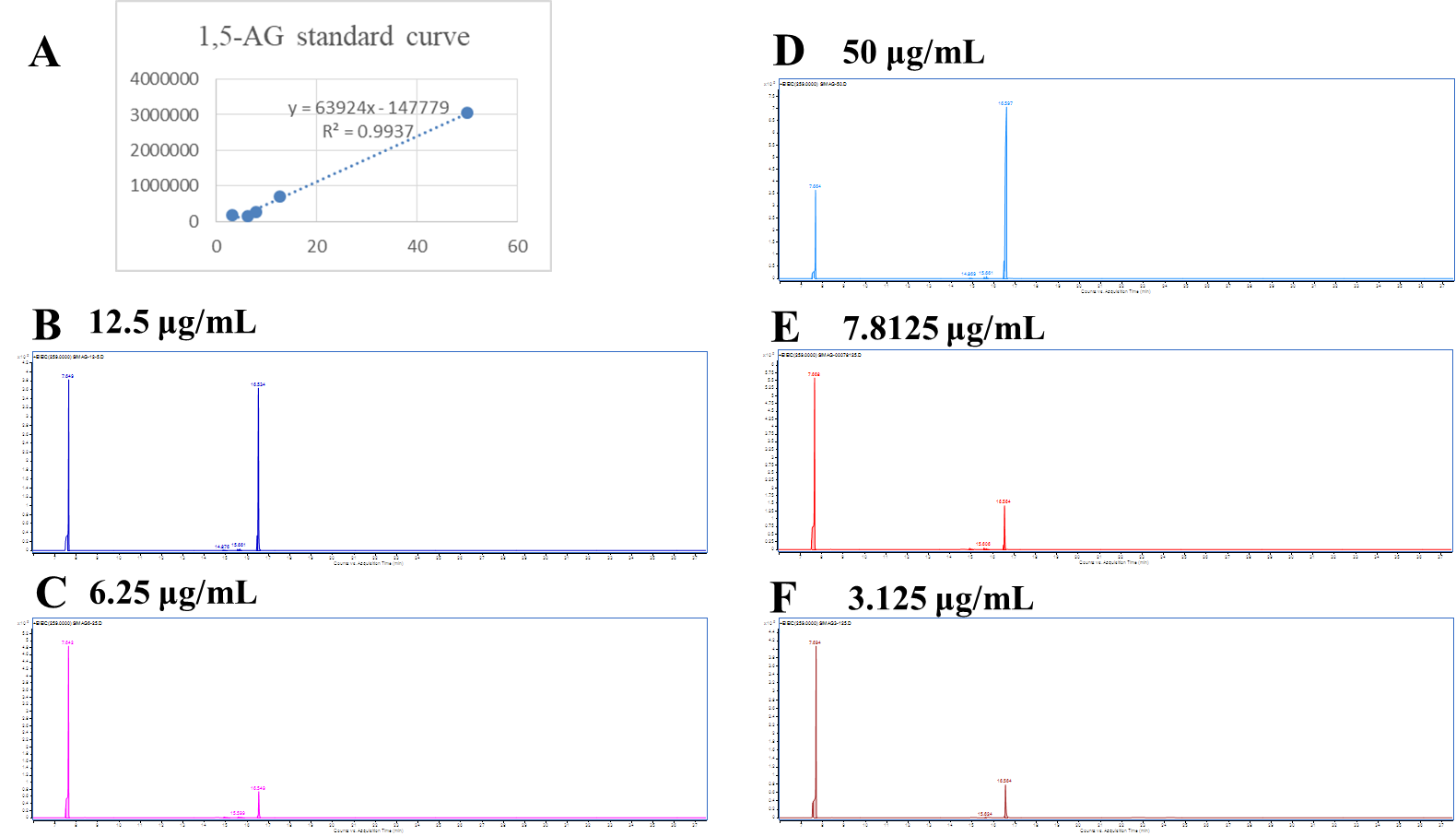
**

**Supplementary Fig. 18.** Calibration curve and representative chromatograms of 1,5-AG by GC-MS. (A) Concentration curve of 1,5-AG. (B-F) Representative chromatograms of 1,5-AG at different concentrations using SIM mode at m/z 259.
